# Supplementary material for: Hydrogen Bond Assisted Three-Component Tandem Reactions to Access N-Alkyl-4-Quinolones
Source: Molecules. 2023 Mar 2;28(5):2304. doi: 10.3390/molecules28052304 (PMC10005641; doi:10.3390/molecules28052304)
Supplement: Supplementary file 1 [file molecules-28-02304-s001.zip › Supporting Information.pdf]

*Supporting Information for*

# Hydrogen Bond Assisted Three-component Tandem Reactions to Access *N*-Alkyl-4-quinolones

**Huanhuan Liu[1,2,‡] Huadan Liu[1,2,‡] Enhua Wang[3], Liangqun Li[1,2], Zhongsheng Luo[1,2], Jiafu Cao[1,2], Jialin Chen[1,2], Lishou Yang[1,2,*] and Xiaosheng Yang[1,2,*]**

[1]*State Key Laboratory of Functions and Applications of Medicinal Plants, Guizhou Medical University, Guiyang 550014, China*

[2]*The Key Laboratory of Chemistry for Natural Products of Guizhou Province and Chinese Academy of Sciences, Guiyang 550014, China*

[3]*Department of Food and Medicine, Guizhou Vocational College of Agriculture, Guiyang 550014, China*

*Authors for correspondence: gzcnp@sina.cn (X.Yang); 1039160204@qq.com (L. Yang).

Corresponding authors at: State Key Laboratory of Functions and Applications of Medicinal Plants, Guizhou Medical University, Guiyang 550014, China

1. Optimization Studies for the Synthesis of 4-Quinolone **4a**........................................................2
2. $^{31}$P NMR Control Experiments..………….....……………………………………..………3
3. Optimization Studies for the Synthesis of 1-Phenyl-4-quinolone **4ap**……………………...…4
4. Protective Effects of 4-Quinolones on NMDA Toxicity…………………………………………4
5. Molecular Docking Study………………………………………………………………...…5
6. $^{1}$H NMR & $^{13}$C NMR Spectra of the Products………………………………………….....….6
7. DFT Calculation………………………………......…………………………………….…50
8. References……………………………………………………………………………………54

## 1. Optimization Studies for the Synthesis of 4-Quinolone 4a

**Table S1.** Evaluation of PPA amount, time and temperature [a].

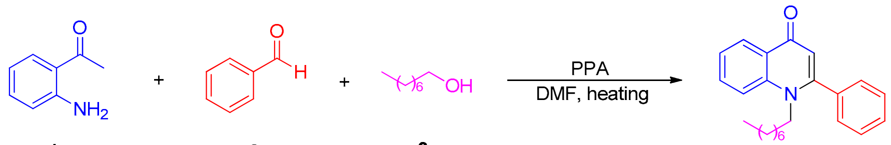


1a 2a 3a 4a

| Entry | PPA (equiv.) | Time (h) | *T* (°C) | 4a (%)[b] |
|---|---|---|---|---|
| 1 | 5.0 | 1 | reflux | 38 |
| 2 | 3.0 | 1 | reflux | 45 |
| 3 | 1.5 | 1 | reflux | 51 |
| 4 | 1.0 | 1 | reflux | 50 |
| 5 | 0.5 | 1 | reflux | 37 |
| 6 | 1.0 | 2 | reflux | 67 |
| 7 | 1.0 | 3 | reflux | 81 |
| 8 | 1.0 | 4 | reflux | 70 |
| 9 | 1.0 | 3 | 80 | 21 |
| 10[c] | 1.0 | 3 | 100 | 17 |
| 11[d] | 1.0 | 3 | 100 | 41 |
| 12[e] | 1.0 | 3 | reflux | 49 |
| 13[f] | 1.0 | 3 | reflux | 34 |
| 14[g] | 1.0 | 3 | reflux | 32 |

[a] Reaction conditions: **1a** (0.37 mmol), **2a** (0.44 mmol), **3a** (1 mL), DMF (0.5 mL). [b] Isolated yield. [c] Reaction carried out in a pressure-resistant reaction bottle. [d] Under microwave irradiation. [e] Reaction conditions: **1a** (0.37 mmol), **2a** (0.44 mmol), **3a** (1 mL), Pd/C (5 mol%), DMF (0.5 mL). [f] Reaction conditions: **1a** (0.37 mmol), **2a** (0.44 mmol), **3a** (1 mL), $PdCl_2$ (5 mol%), DMF (0.5 mL). [g] Reaction conditions: **1a** (0.37 mmol), **2a** (0.44 mmol), ethanol **3c** (1 mL), Pd/C (5 mol%), DMF (0.5 mL).

## 2. $^{31}$P NMR Control Experiments

In order to detect the PPE-**7** complex, we performed $^{31}$P NMR experiments. The results shown that PPA-**7** complex was not detected (Figure S1d *vs.* S1c), and that PPA reacted with alcohol **3c** to afford PPE **6** (Figure S1d *vs.* S1b). As shown in Figure S1a, a new peak was observed, which might be identified to the PPE-**7** complex **8**.

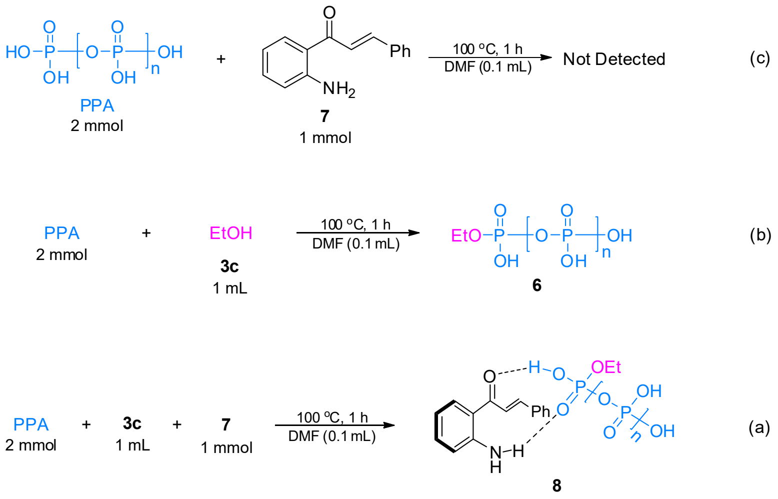


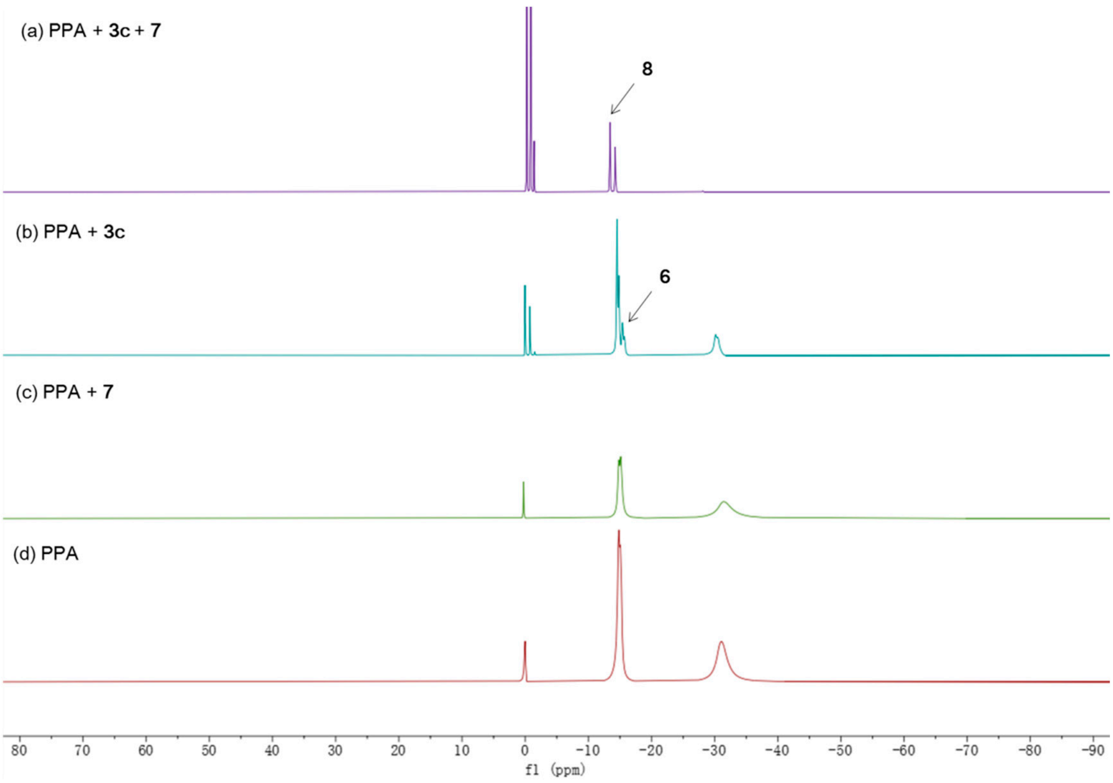


**Figure S1.** $^{31}$P NMR analysis of the PPE-**7** complex.

## 3. Optimization Studies for the Synthesis of 1-Phenyl-4-quinolone 4ap

**Table S2.** Optimization of reaction conditions [a].

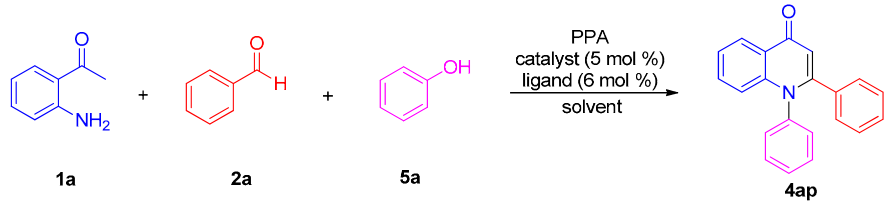


| Entry | PPA (equiv.) | Catalyst | Ligand | *T* (°C) | Time (h) | Solvent | 4ap (%)[b] |
|---|---|---|---|---|---|---|---|
| 1 | 1.0 | $PdCl_2$ | | 120 | 3 | DCE | 59 |
| 2 | 0.5 | $PdCl_2$ | | 120 | 3 | DCE | 43 |
| 3 | 1.5 | $PdCl_2$ | | 120 | 3 | DCE | 56 |
| 4 | 1.0 | Pd/C | | 120 | 3 | DCE | 70 |
| 5 | 1.0 | $Pd(OAc)_2$ | | 120 | 3 | DCE | 55 |
| 6 | 1.0 | $Pd(TFA)_2$ | | 120 | 3 | DCE | 51 |
| 7 | 1.0 | $PdCl_2(CH_3CN)_2$ | | 120 | 3 | DCE | 37 |
| 8 | 1.0 | Pd/C | DPPB | 120 | 3 | DCE | 2 |
| 9 | 1.0 | Pd/C | | 100 | 3 | DCE | 19 |
| 10 | 1.0 | Pd/C | | 140 | 3 | DCE | 57 |
| 11 | 1.0 | Pd/C | | 120 | 1.5 | DCE | 28 |
| 12 | 1.0 | Pd/C | | 120 | 4.5 | DCE | 62 |
| 13 | 1.0 | Pd/C | | 120 | 3 | DMF | ND |
| 14 | 1.0 | Pd/C | | 120 | 3 | DCM | 52 |
| 15 | 1.0 | Pd/C | | 120 | 3 | toluene | 35 |
| 16 | 1.0 | Pd/C | | 120 | 3 | 1,4-dioxane | ND |
| 17 | 1.0 | Pd/C | | 120 | 3 | acetone | ND |
| 18[c] | 1.0 | Pd/C | | 120 | 3 | DCE | 3 |

[a] Reaction conditions: **1a** (0.37 mmol), **2a** (0.44 mmol), **5a** (0.37 mmol), solvent (1.5 mL). [b] Isolated yield. [c] Reaction conditions: **1a** (0.37 mmol), **2a** (0.44 mmol), **3a** (1 mL), DCE (0.5 mL). ND referred to "not detected".

## 4. Protective Effects of 4-Quinolones on NMDA Toxicity

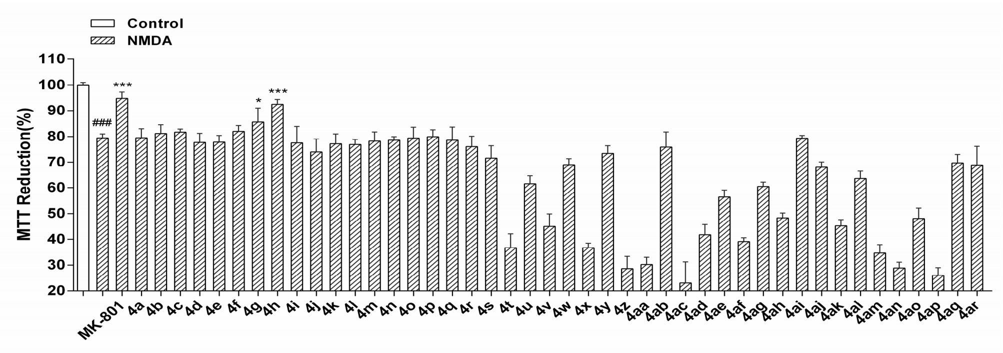


**Figure S2.** Protective effects of 4-quinolones at 20 μM on NMDA-induced injury in PC12 cells using the MTT assay. The injury was determined by MTT assay. Data are presented as mean ± SD (n = 3). MK-801 was used as positive control. ###p < 0.001 as compared to control group, *p < 0.05, ***p < 0.001 as compared to NMDA group.

## 5. Molecular Docking Study

Docking study of the most active compound **4h** was performed using AutoDock 4 software package. Crystal structure of NMDA receptor in complex with DCKA and glutamate (PDB: 4NF4) was used. For active site docking, a grid box with size 40 × 40 × 40 Å centered at the center of co-crystalized ligand (DCKA) was selected. The pose with the best AutoDock score was chosen for further analysis. Images were rendered using The PyMOL Molecular Graphics System 2.4.

As shown in Figure S3, Compound **4h** being stabilized by Pi-stacking interaction with PHE-92 and cation-Pi interaction between the nitrogen atom and PHE-92. Compound **4h** formed hydrogen bonds with the amino acid residues ARG-131 and THR-126. Additionally, the S moiety of thiazole ring could generate a polar interaction with residue GLN-13.

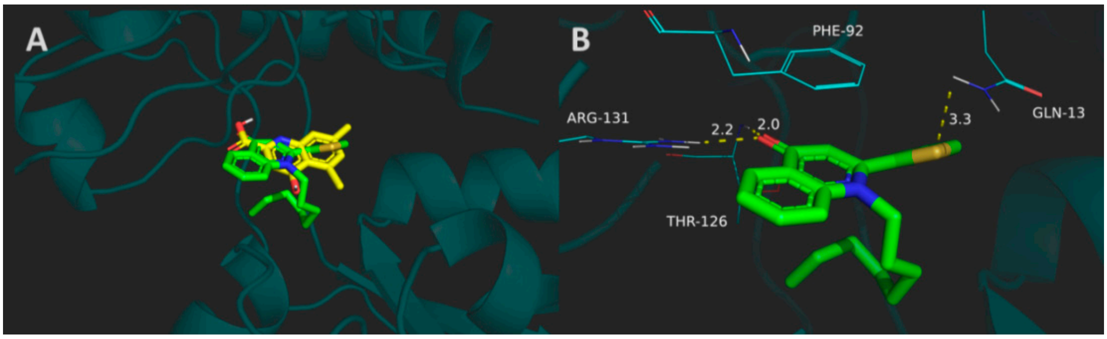


**Figure S3.** Docking of compound **4h** with glycine binding site of NMDA receptor (PDB: 4NF4). (A) Alignment of **4h** (green) and co-crystallized ligand DCKA (yellow) in the active site. (B) The interactions of **4h** with the active site residues. Hydrogen bond interactions were shown in yellow dotted lines.

## 6. $^{1}$H NMR & $^{13}$C NMR Spectra of the Products

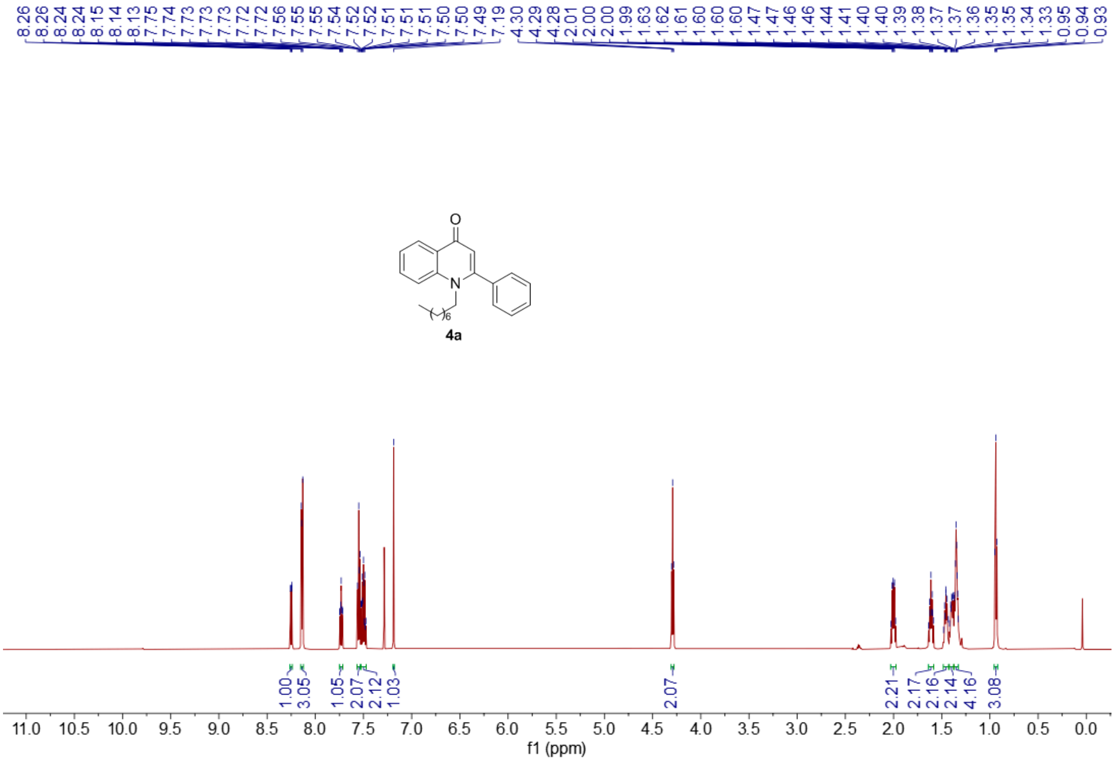


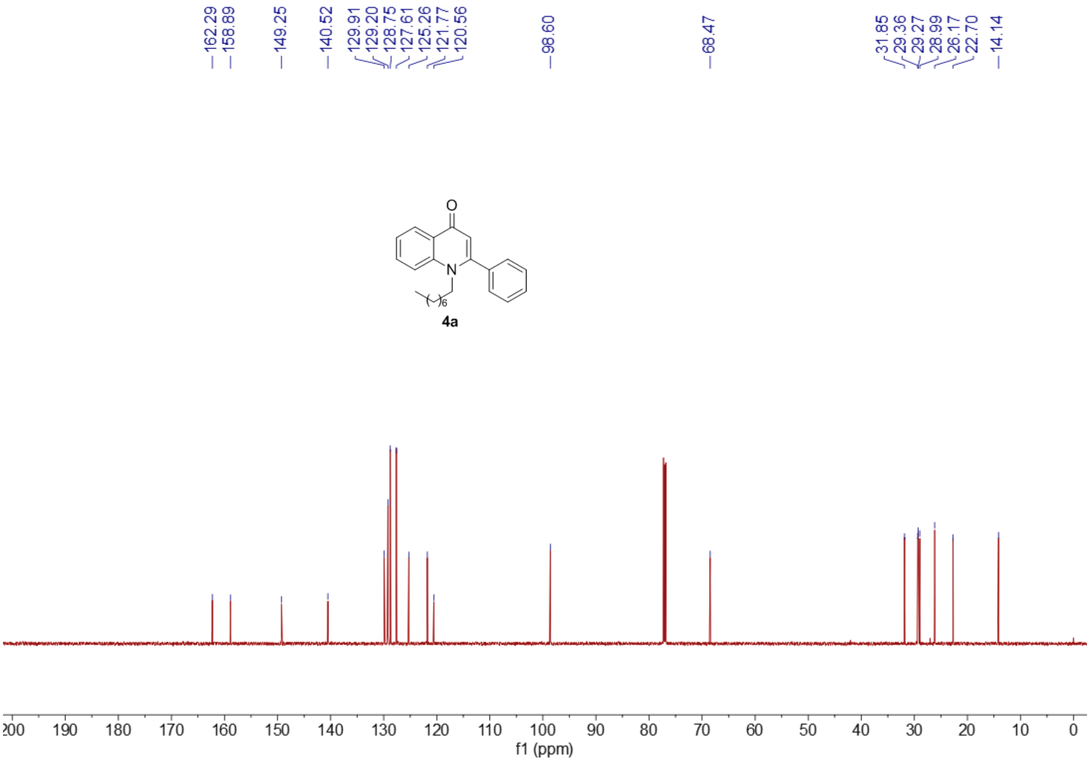

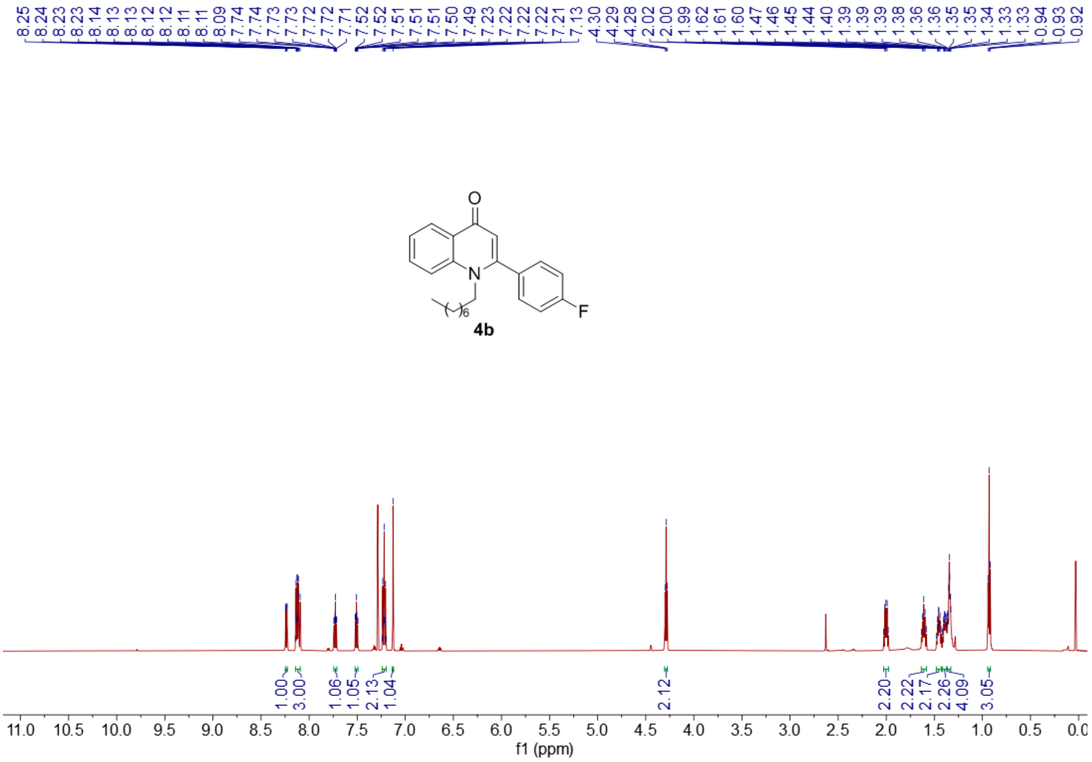
8.25 8.24 8.23 8.23 8.14 8.13 8.13 8.12 8.12 8.11 8.11 8.09 7.74 7.74 7.73 7.73 7.72 7.72 7.71 7.52 7.52 7.51 7.51 7.50 7.49 7.23 7.22 7.22 7.22 7.21 7.13 4.30 4.29 4.28 2.02 2.00 1.99 1.62 1.61 1.60 1.47 1.46 1.45 1.44 1.40 1.39 1.39 1.39 1.38 1.36 1.36 1.35 1.35 1.34 1.33 1.33 0.94 0.93 0.92
4b
1.00 3.00 1.06 1.05 2.13 1.04 2.12 2.20 2.22 2.17 2.26 4.09 3.05
f1 (ppm)

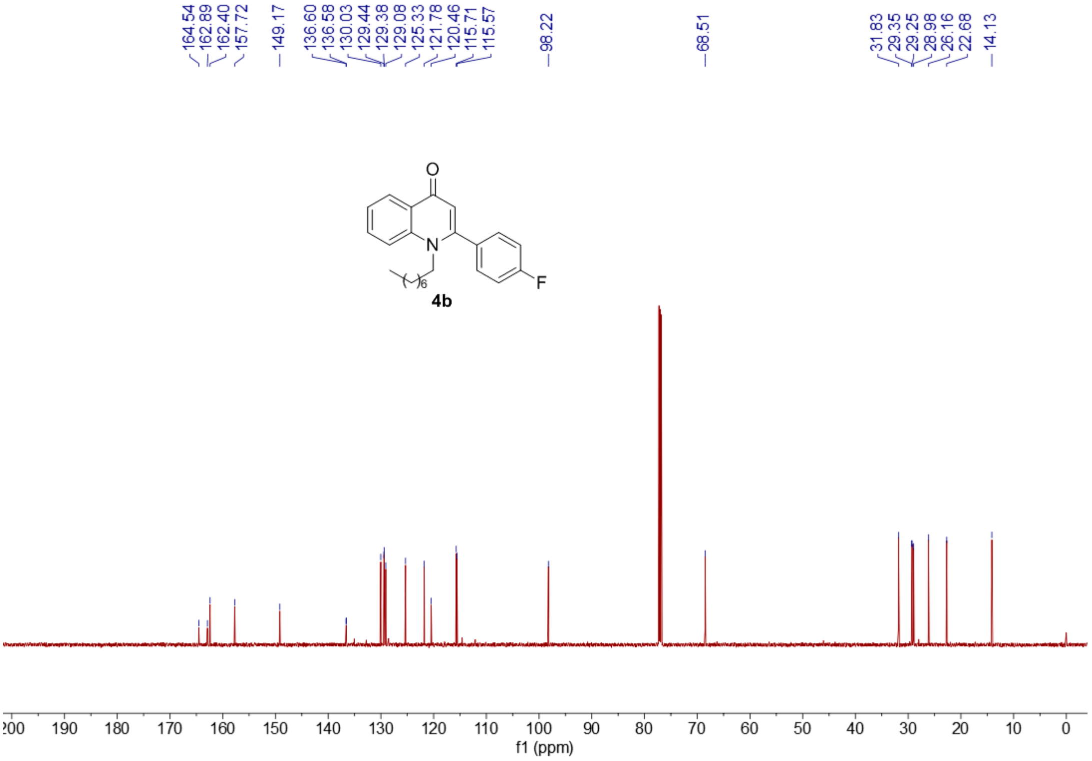
164.54 162.89 162.40 157.72 149.17 136.60 136.58 130.03 129.44 129.38 129.08 125.33 121.78 120.46 115.71 115.57 98.22 68.51 31.83 29.35 29.25 28.98 26.16 22.68 14.13
4b
f1 (ppm)

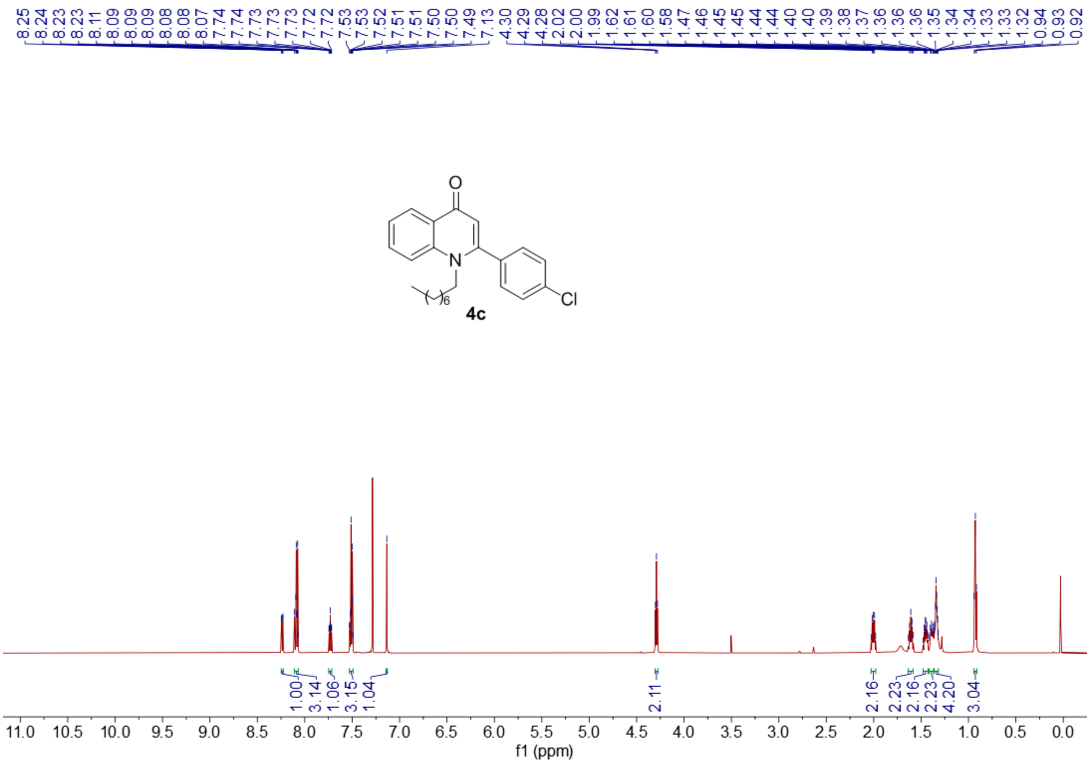
8.25 8.24 8.23 8.23 8.11 8.09 8.09 8.09 8.08 8.08 8.07 7.74 7.74 7.73 7.73 7.73 7.72 7.72 7.53 7.53 7.52 7.51 7.51 7.50 7.50 7.49 7.13 4.30 4.29 4.28 2.02 2.00 1.99 1.62 1.61 1.60 1.58 1.47 1.46 1.45 1.45 1.44 1.44 1.40 1.40 1.39 1.38 1.37 1.36 1.36 1.36 1.35 1.34 1.34 1.33 1.33 1.32 0.94 0.93 0.92
O
N
Cl
6
4c
1.00 3.14 1.06 3.15 1.04 2.11 2.16 2.23 2.16 2.23 4.20 3.04
11.0 10.5 10.0 9.5 9.0 8.5 8.0 7.5 7.0 6.5 6.0 5.5 5.0 4.5 4.0 3.5 3.0 2.5 2.0 1.5 1.0 0.5 0.0
f1 (ppm)

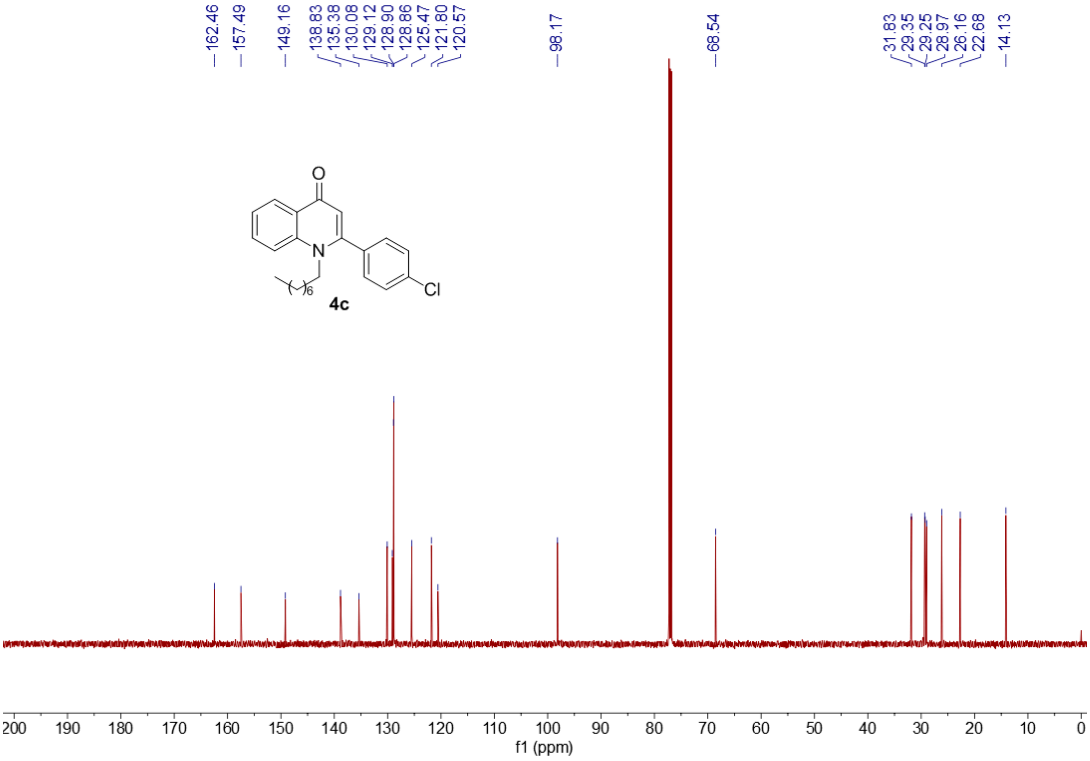
162.46 157.49 149.16 138.83 135.38 130.08 129.12 128.90 128.86 125.47 121.80 120.57 98.17 68.54 31.83 29.35 29.25 28.97 26.16 22.68 14.13
O
N
Cl
6
4c
200 190 180 170 160 150 140 130 120 110 100 90 80 70 60 50 40 30 20 10 0
f1 (ppm)

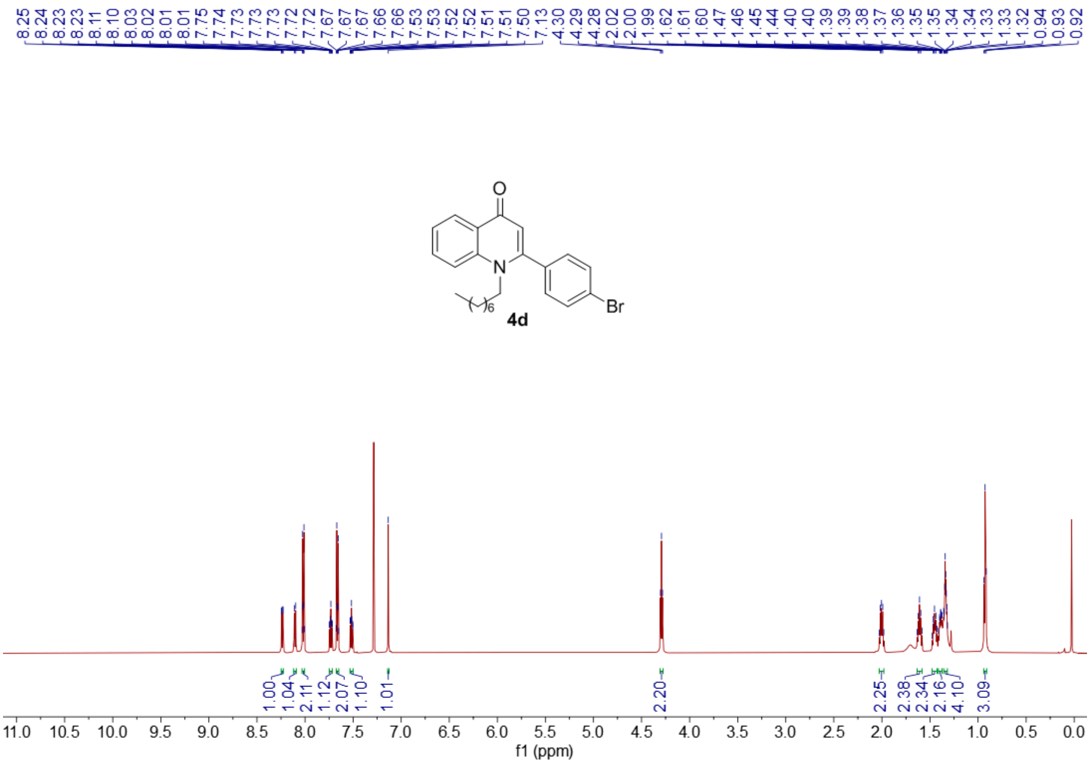

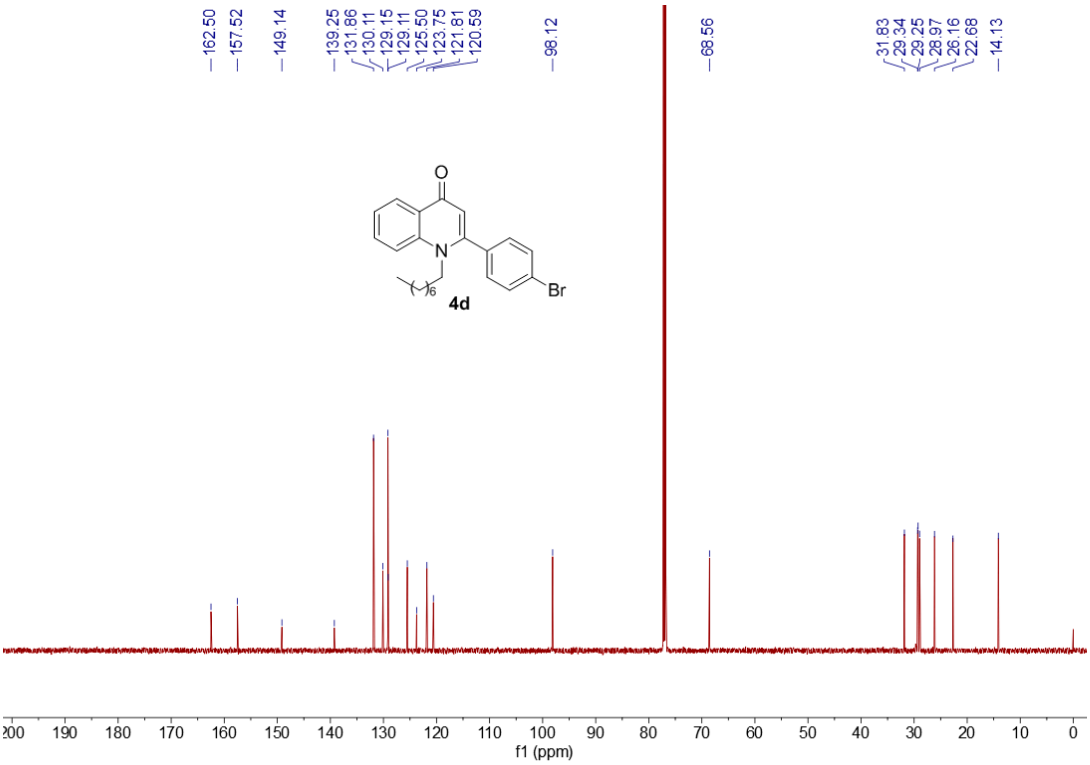

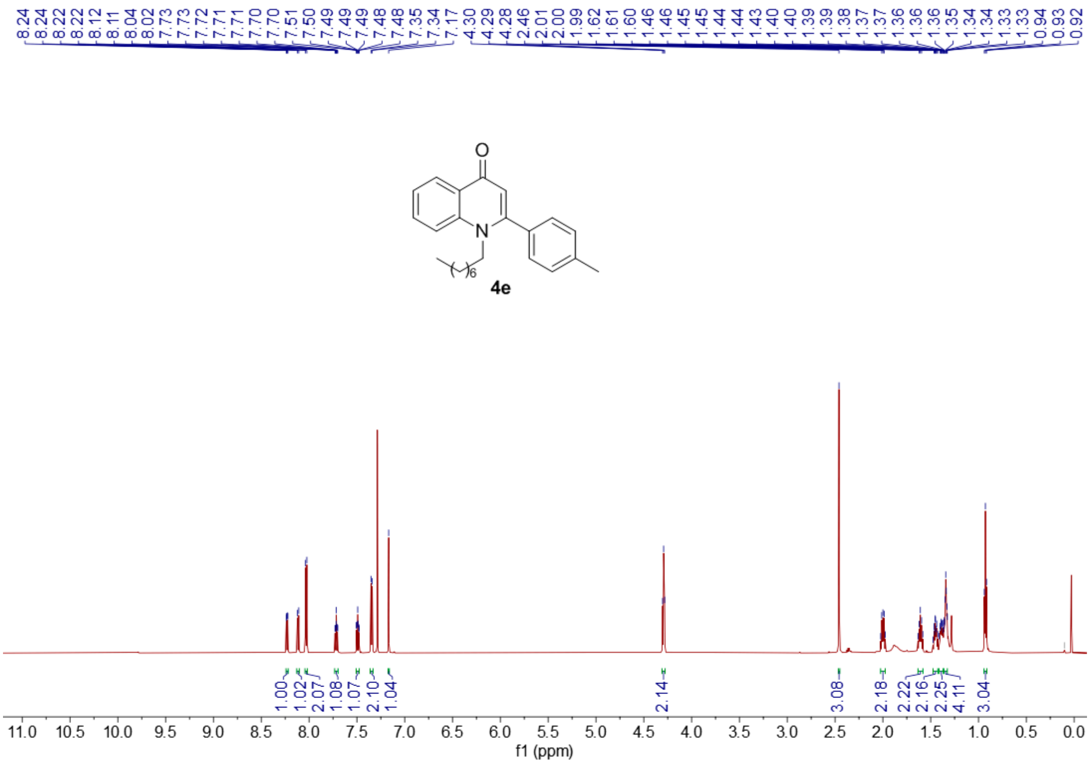

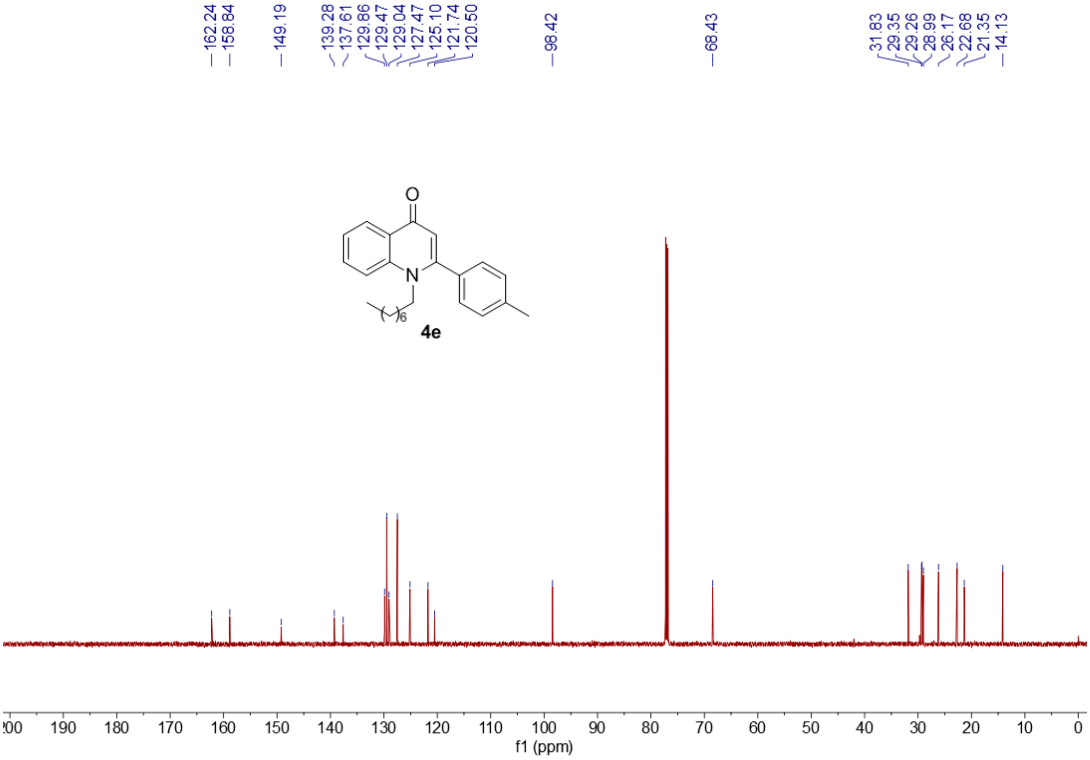

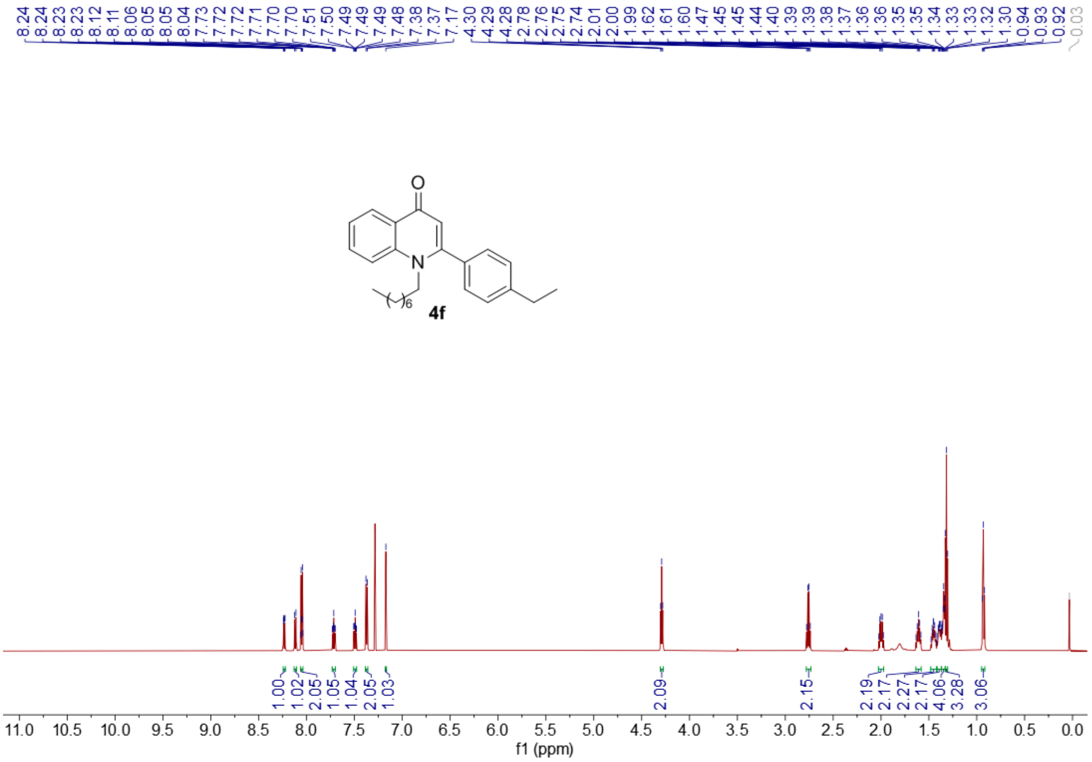
8.24 8.24 8.23 8.23 8.12 8.11 8.06 8.05 8.04 7.73 7.72 7.72 7.71 7.70 7.70 7.51 7.50 7.49 7.49 7.49 7.48 7.38 7.37 7.17 4.30 4.29 4.28 2.78 2.76 2.75 2.74 2.01 2.00 1.99 1.62 1.61 1.60 1.47 1.45 1.45 1.44 1.40 1.39 1.39 1.38 1.37 1.36 1.36 1.35 1.34 1.33 1.33 1.32 1.30 0.94 0.93 0.92 0.03
O
N
6
4f
1.00 1.02 2.05 1.05 1.04 2.05 1.03 2.09 2.15 2.19 2.17 2.27 2.17 4.06 3.28 3.06
11.0 10.5 10.0 9.5 9.0 8.5 8.0 7.5 7.0 6.5 6.0 5.5 5.0 4.5 4.0 3.5 3.0 2.5 2.0 1.5 1.0 0.5 0.0
f1 (ppm)

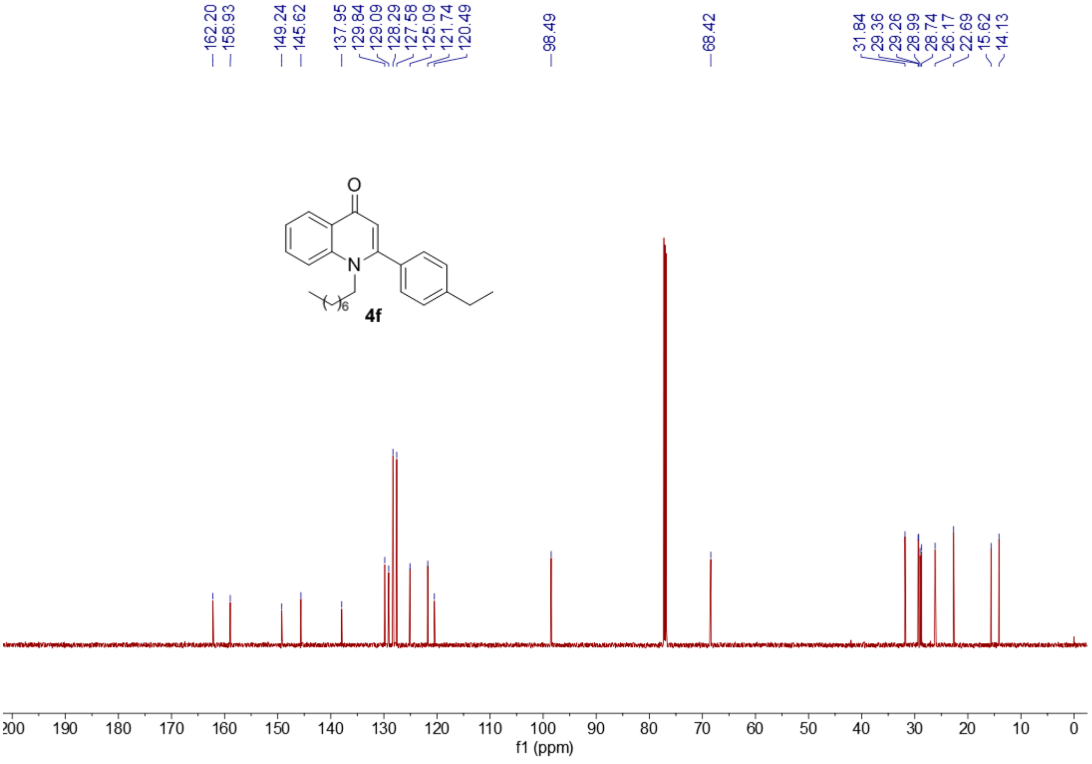
162.20 158.93 149.24 145.62 137.95 129.84 129.09 128.29 127.58 125.09 121.74 120.49 98.49 68.42 31.84 29.36 29.26 28.99 28.74 26.17 22.69 15.62 14.13
O
N
6
4f
200 190 180 170 160 150 140 130 120 110 100 90 80 70 60 50 40 30 20 10 0
f1 (ppm)

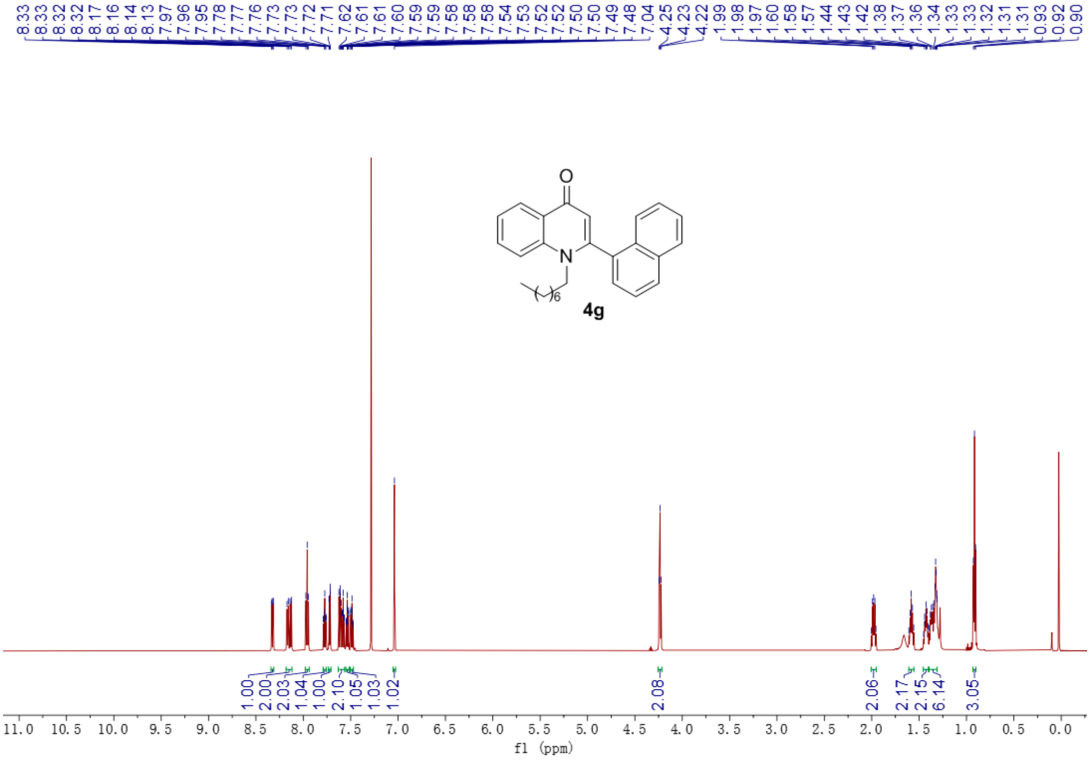
8.33
8.33
8.32
8.32
8.17
8.16
8.14
8.13
7.97
7.96
7.95
7.78
7.77
7.76
7.73
7.73
7.72
7.71
7.62
7.61
7.61
7.60
7.59
7.59
7.58
7.58
7.54
7.53
7.52
7.52
7.50
7.49
7.48
7.04
4.25
4.23
4.22
1.99
1.98
1.97
1.60
1.58
1.57
1.44
1.43
1.42
1.38
1.37
1.36
1.34
1.33
1.33
1.32
1.31
0.93
0.92
0.90
O
N
6
4g
1.00
2.00
2.03
1.04
1.00
2.10
1.05
1.03
1.02
2.08
2.06
2.17
2.15
6.14
3.05
fl (ppm)

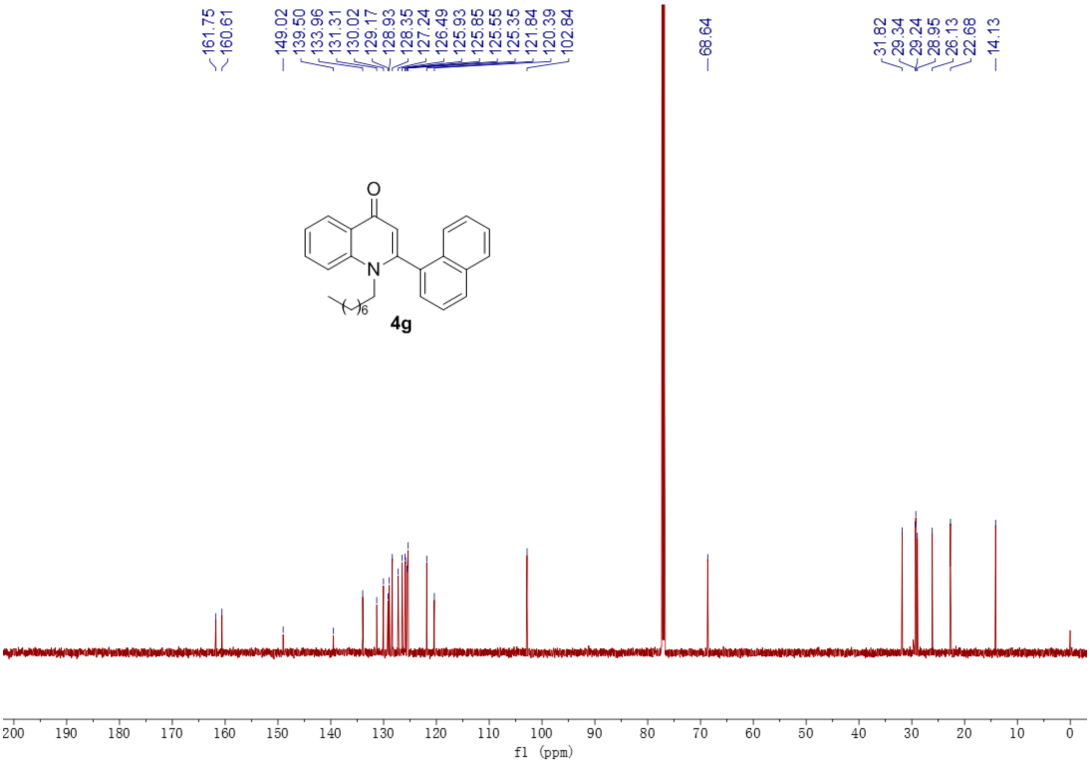
161.75
160.61
149.02
139.50
133.96
131.31
130.02
129.17
128.93
128.35
127.24
126.49
125.93
125.85
125.55
125.35
121.84
120.39
102.84
68.64
31.82
29.34
29.24
28.95
26.13
22.68
14.13
O
N
6
4g
fl (ppm)

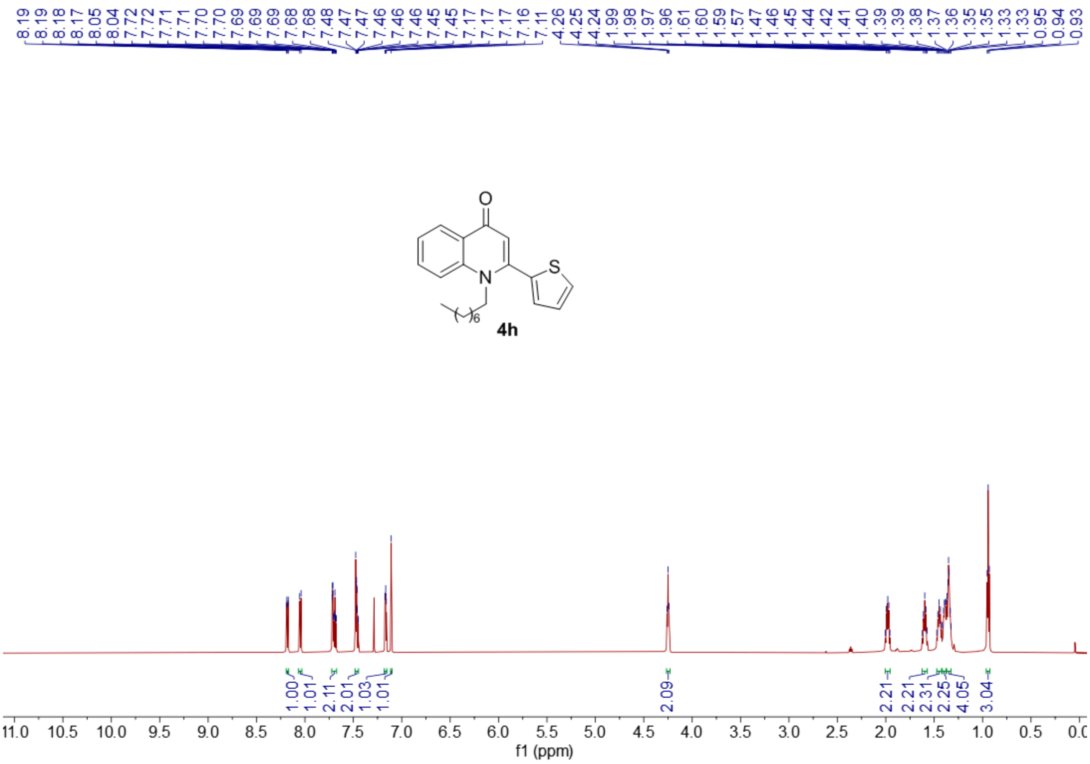

8.19 8.19 8.18 8.17 8.05 8.04 7.72 7.72 7.71 7.71 7.70 7.70 7.69 7.69 7.69 7.68 7.68 7.48 7.47 7.47 7.46 7.46 7.46 7.45 7.45 7.17 7.17 7.17 7.16 7.11 4.26 4.25 4.24 1.99 1.98 1.97 1.96 1.61 1.60 1.59 1.57 1.47 1.46 1.45 1.44 1.42 1.41 1.40 1.39 1.39 1.38 1.37 1.36 1.35 1.35 1.33 0.95 0.94 0.93
O
N
S
6
4h
1.00 1.01 2.11 2.01 1.03 1.01
2.09
2.21 2.21 2.31 2.25 4.05 3.04
f1 (ppm)


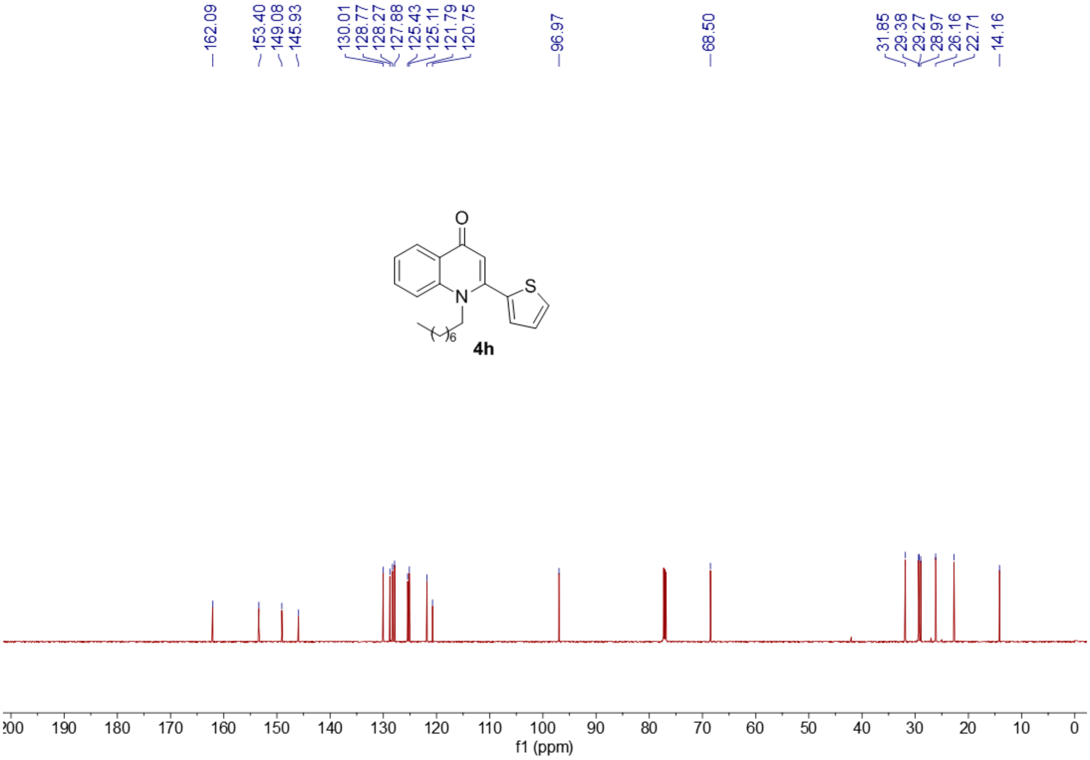

162.09 153.40 149.08 145.93 130.01 128.77 128.27 127.88 125.43 125.11 121.79 120.75 96.97 68.50 31.85 29.38 29.27 28.97 26.16 22.71 14.16
O
N
S
6
4h
f1 (ppm)

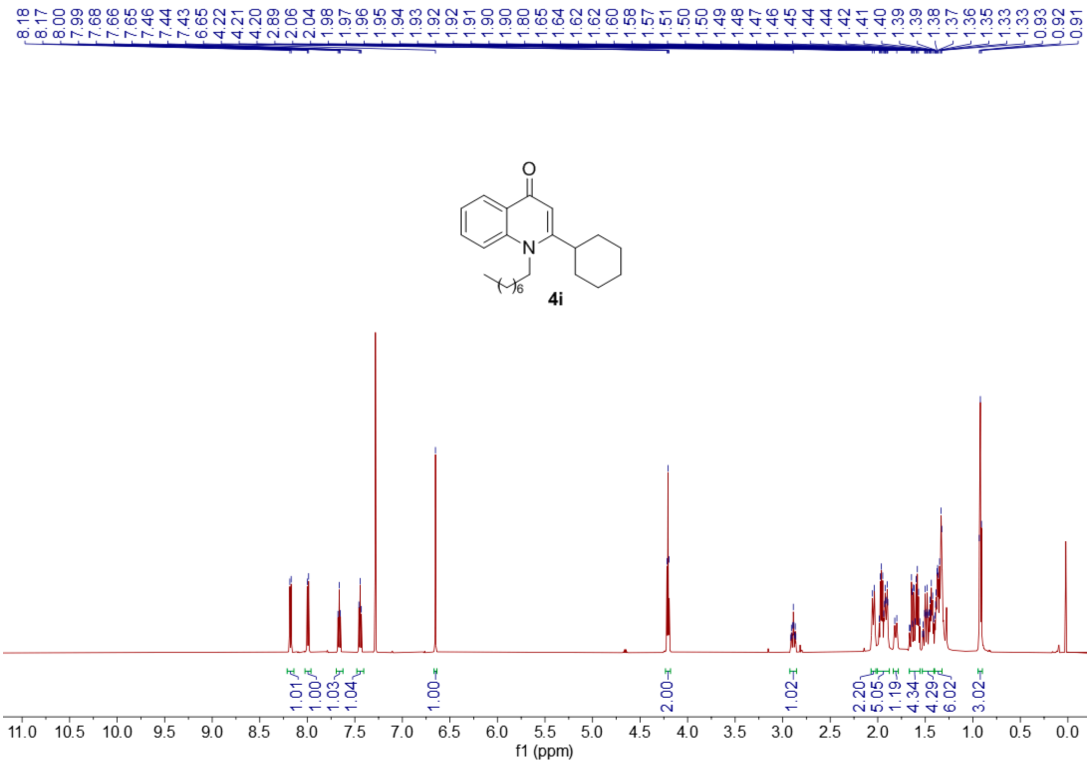
8.18
8.17
8.00
7.99
7.68
7.66
7.65
7.46
7.44
7.43
6.65
4.22
4.21
4.20
2.89
2.06
2.04
1.98
1.97
1.96
1.95
1.94
1.93
1.92
1.92
1.91
1.90
1.90
1.80
1.65
1.64
1.62
1.60
1.58
1.57
1.51
1.50
1.50
1.49
1.48
1.47
1.46
1.45
1.44
1.44
1.42
1.41
1.40
1.39
1.39
1.38
1.37
1.36
1.35
1.33
0.93
0.92
0.91
O
N
6
4i
1.01
1.00
1.03
1.04
1.00
2.00
1.02
2.20
5.05
1.19
4.34
4.29
6.02
3.02
11.0 10.5 10.0 9.5 9.0 8.5 8.0 7.5 7.0 6.5 6.0 5.5 5.0 4.5 4.0 3.5 3.0 2.5 2.0 1.5 1.0 0.5 0.0
f1 (ppm)

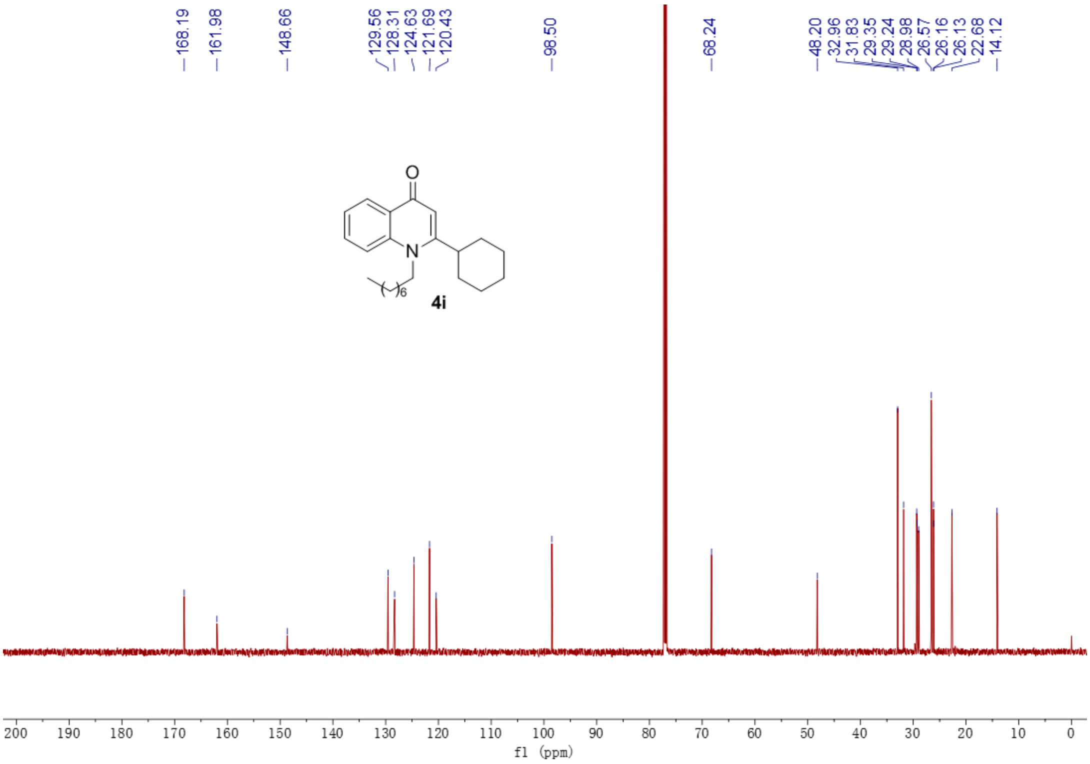
168.19
161.98
148.66
129.56
128.31
124.63
121.69
120.43
98.50
68.24
48.20
32.96
31.83
29.35
29.24
28.98
26.57
26.16
26.13
22.68
14.12
O
N
6
4i
200 190 180 170 160 150 140 130 120 110 100 90 80 70 60 50 40 30 20 10 0
f1 (ppm)

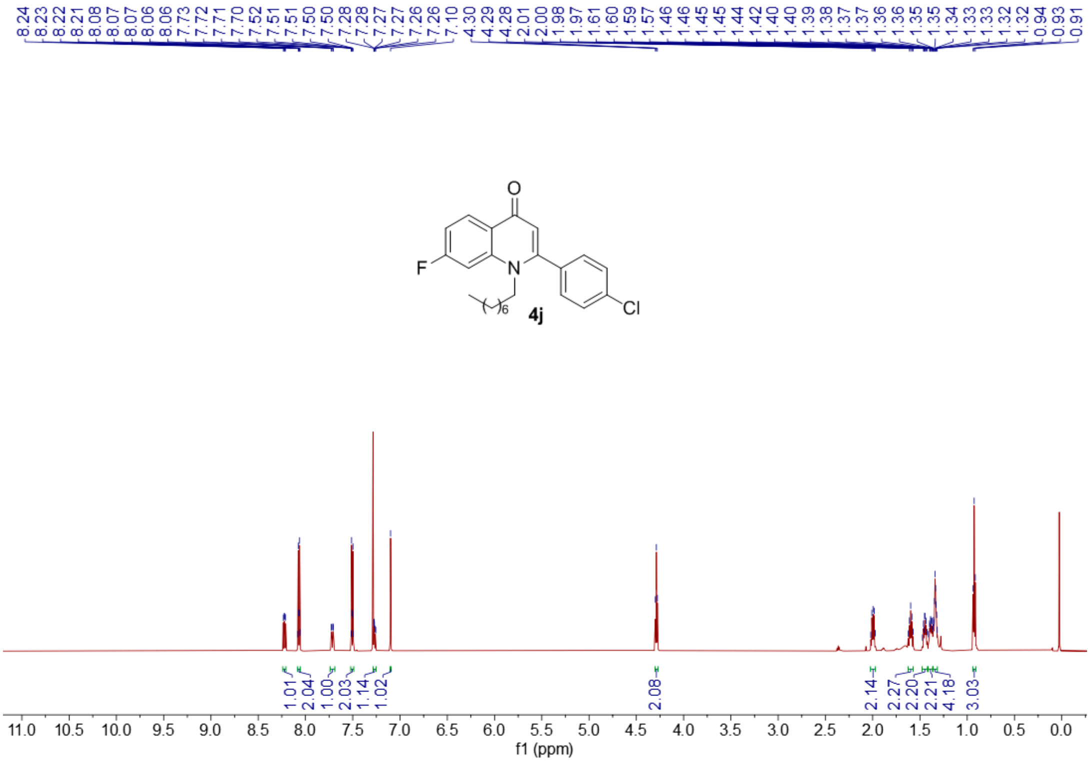
8.24 8.23 8.22 8.21 8.08 8.07 8.07 8.06 8.06 7.73 7.72 7.71 7.70 7.52 7.51 7.51 7.50 7.50 7.28 7.28 7.27 7.27 7.26 7.26 7.10 4.30 4.29 4.28 2.01 2.00 1.98 1.97 1.61 1.60 1.59 1.57 1.46 1.46 1.45 1.45 1.44 1.42 1.40 1.39 1.38 1.37 1.37 1.36 1.36 1.35 1.35 1.34 1.33 1.33 1.32 1.32 0.94 0.93 0.91
O
F
N
Cl
6
4j
1.01 2.04 1.00 2.03 1.14 1.02 2.08 2.14 2.27 2.20 2.21 4.18 3.03
11.0 10.5 10.0 9.5 9.0 8.5 8.0 7.5 7.0 6.5 6.0 5.5 5.0 4.5 4.0 3.5 3.0 2.5 2.0 1.5 1.0 0.5 0.0
f1 (ppm)

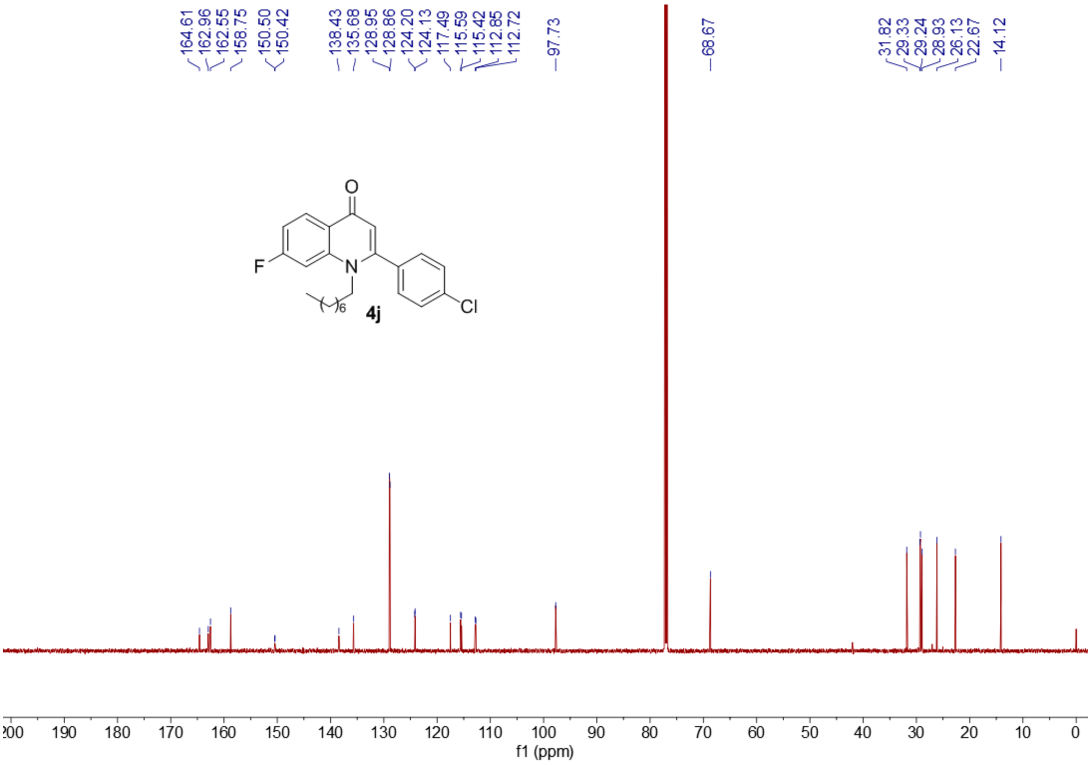
164.61 162.96 162.55 158.75 150.50 150.42 138.43 135.68 128.95 128.86 124.20 124.13 117.49 115.59 115.42 112.85 112.72 97.73 68.67 31.82 29.33 29.24 28.93 26.13 22.67 14.12
O
F
N
Cl
6
4j
200 190 180 170 160 150 140 130 120 110 100 90 80 70 60 50 40 30 20 10 0
f1 (ppm)

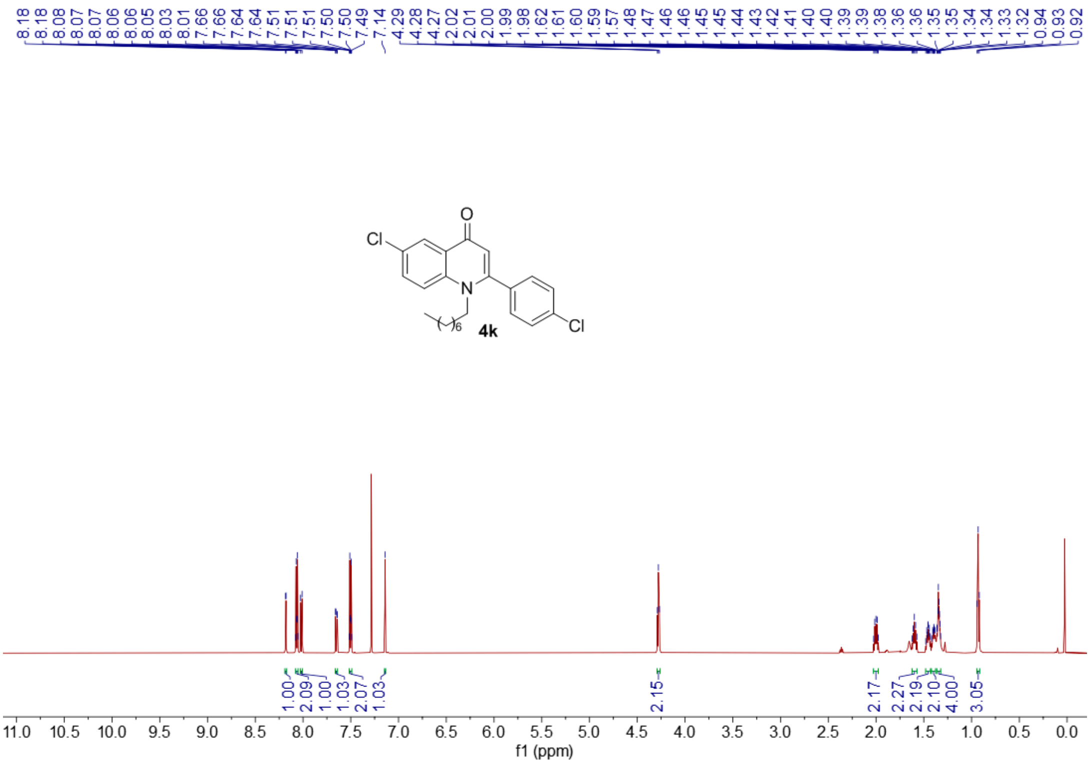

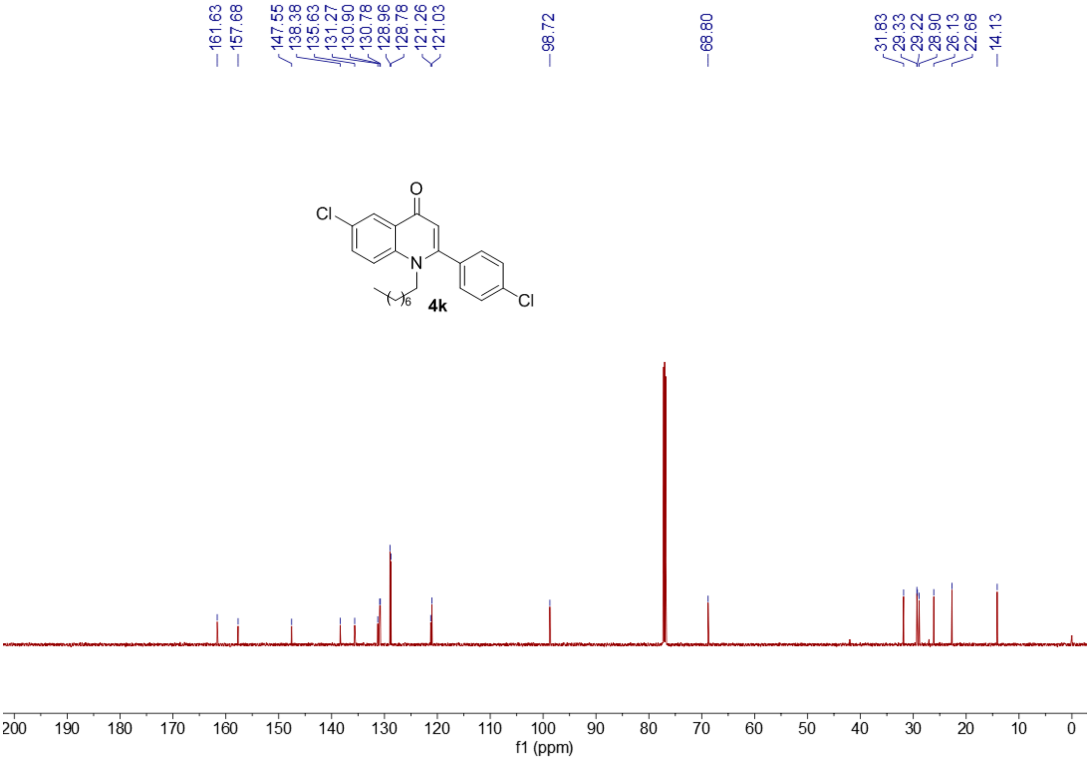

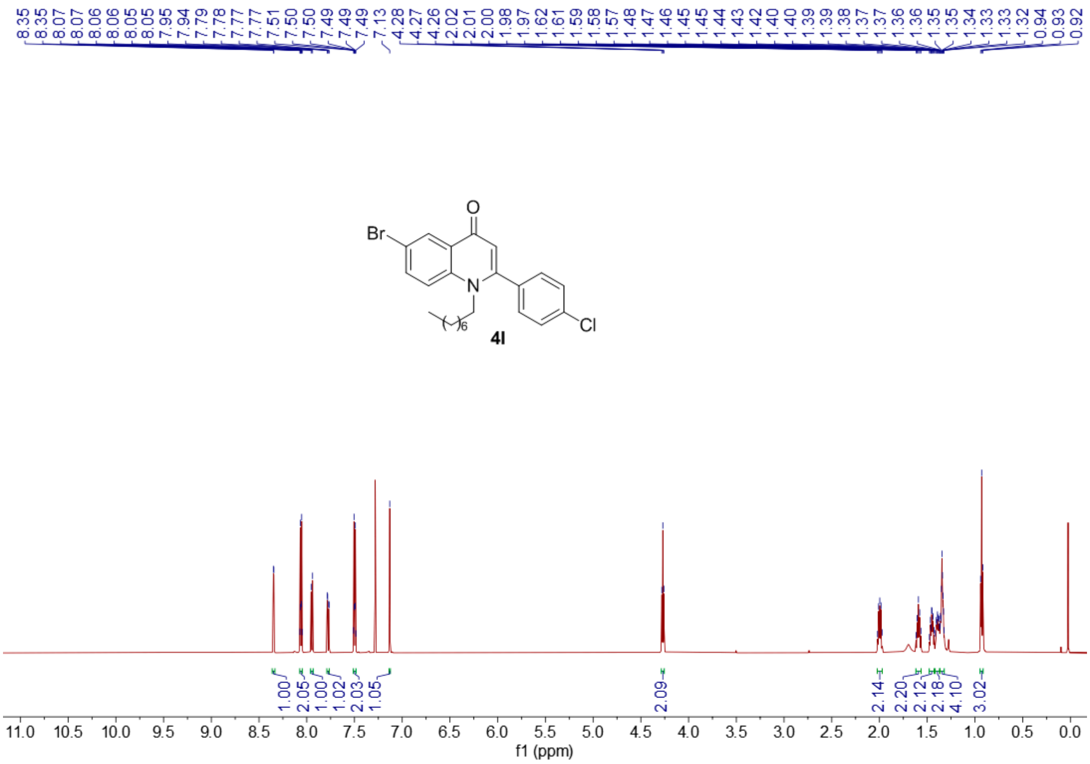
8.35 8.35 8.07 8.07 8.06 8.06 8.05 8.05 7.95 7.94 7.79 7.78 7.78 7.77 7.51 7.50 7.50 7.49 7.49 7.49 7.13 4.28 4.27 4.26 2.02 2.01 2.00 1.98 1.97 1.62 1.61 1.59 1.58 1.57 1.48 1.47 1.46 1.45 1.45 1.44 1.43 1.42 1.40 1.40 1.39 1.39 1.38 1.37 1.37 1.36 1.36 1.35 1.35 1.34 1.33 1.33 1.32 0.94 0.93 0.92
O
Br
N
Cl
6
4l
1.00 2.05 1.00 1.02 2.03 1.05 2.09 2.14 2.20 2.12 2.18 4.10 3.02
11.0 10.5 10.0 9.5 9.0 8.5 8.0 7.5 7.0 6.5 6.0 5.5 5.0 4.5 4.0 3.5 3.0 2.5 2.0 1.5 1.0 0.5 0.0
f1 (ppm)

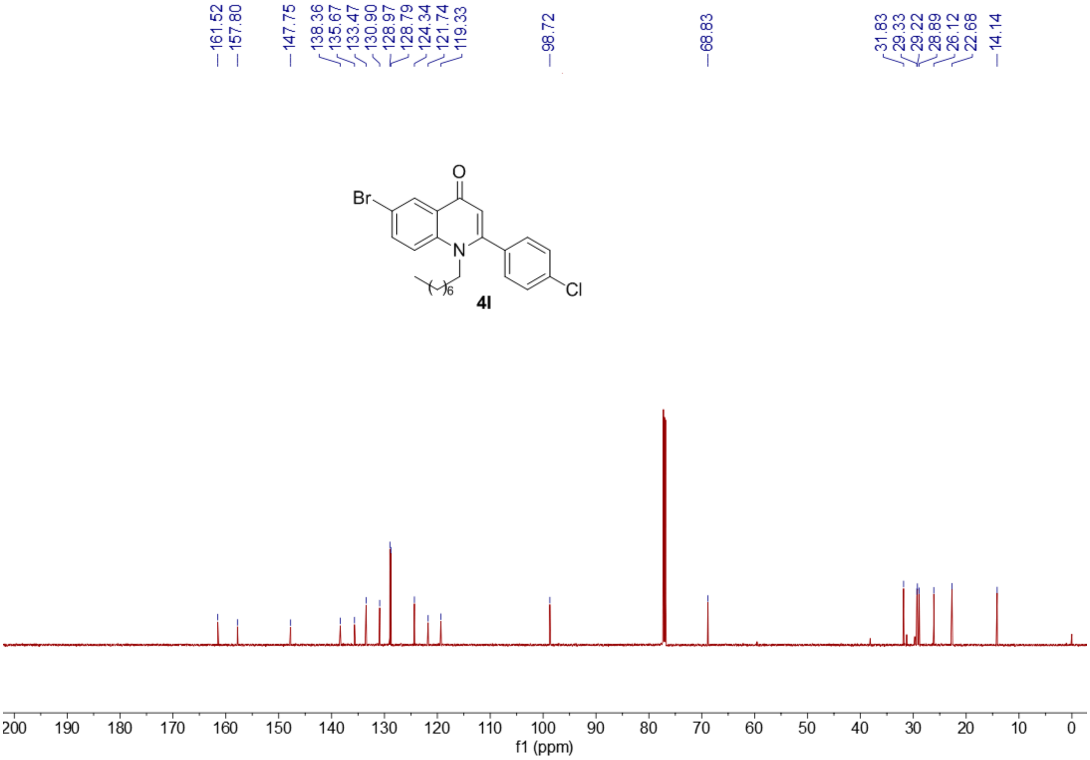
161.52 157.80 147.75 138.36 135.67 133.47 130.90 128.97 128.79 124.34 121.74 119.33 98.72 68.83 31.83 29.33 29.22 28.89 26.12 22.68 14.14
O
Br
N
Cl
6
4l
200 190 180 170 160 150 140 130 120 110 100 90 80 70 60 50 40 30 20 10 0
f1 (ppm)

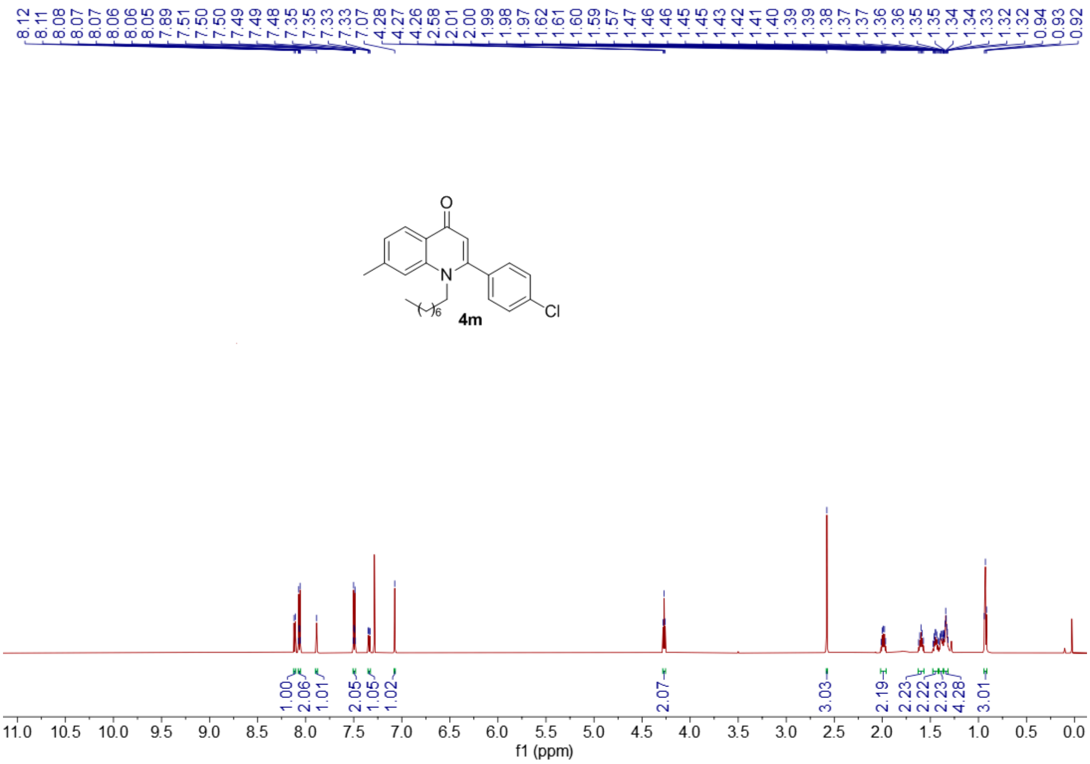
8.12 8.11 8.08 8.07 8.06 8.06 8.05 7.89 7.51 7.50 7.49 7.49 7.48 7.35 7.35 7.33 7.33 7.07 4.28 4.27 4.26 2.58 2.01 2.00 1.99 1.98 1.97 1.62 1.61 1.60 1.59 1.57 1.47 1.46 1.46 1.45 1.45 1.43 1.42 1.41 1.40 1.39 1.39 1.38 1.37 1.37 1.36 1.36 1.35 1.35 1.34 1.34 1.33 1.32 1.32 0.94 0.93 0.92
4m
1.00 2.06 1.01 2.05 1.05 1.02 2.07 3.03 2.19 2.23 2.22 2.23 4.28 3.01
f1 (ppm)

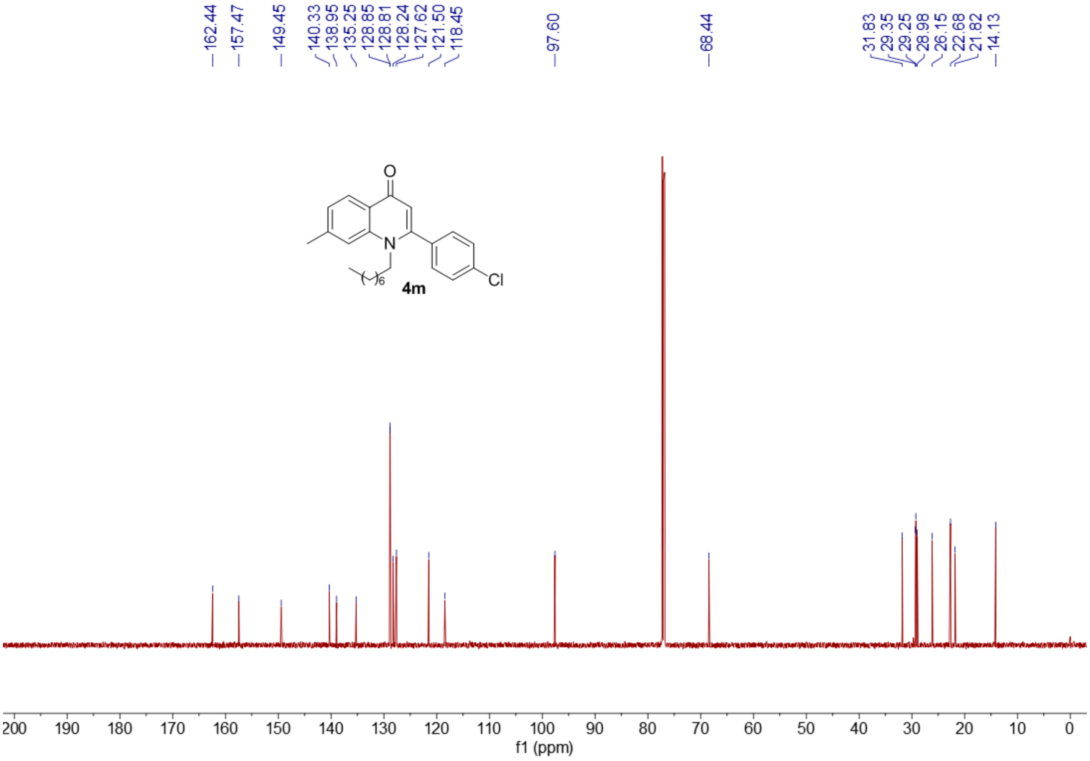
162.44 157.47 149.45 140.33 138.95 135.25 128.85 128.81 128.24 127.62 121.50 118.45 97.60 68.44 31.83 29.35 29.25 28.98 26.15 22.68 21.82 14.13
4m
f1 (ppm)

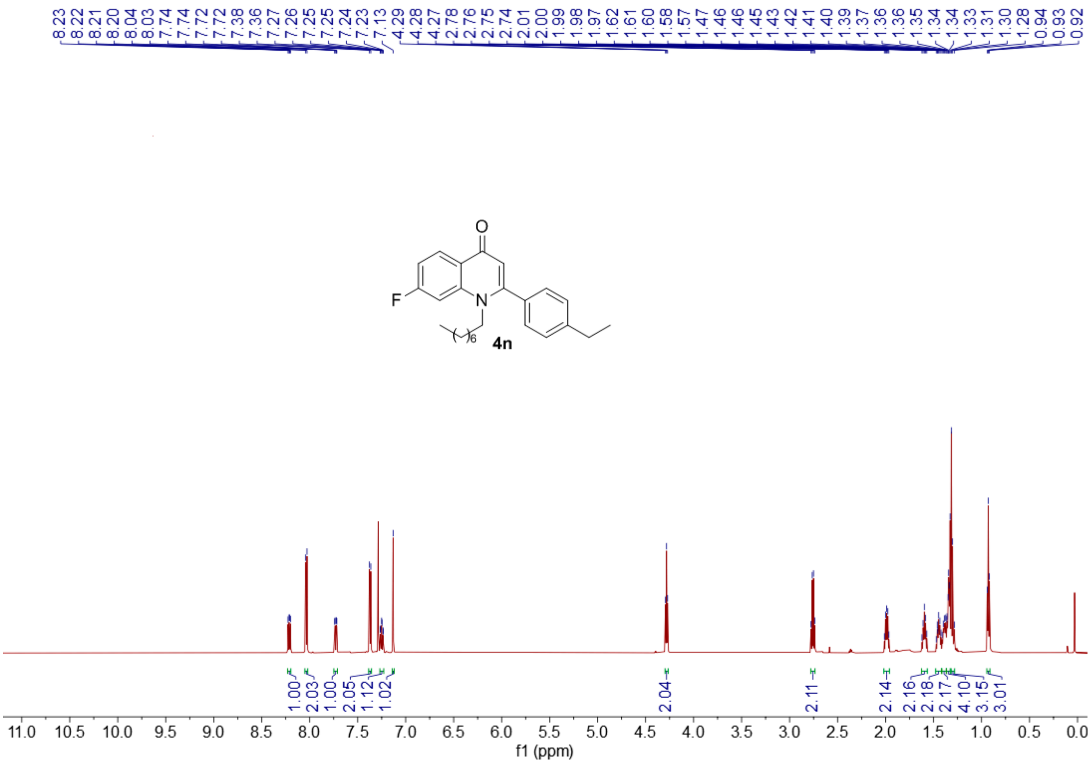
8.23
8.22
8.21
8.20
8.04
8.03
7.74
7.74
7.72
7.72
7.38
7.36
7.27
7.26
7.25
7.25
7.24
7.23
7.13
4.29
4.28
4.27
2.78
2.76
2.75
2.74
2.01
2.00
1.99
1.98
1.97
1.62
1.61
1.60
1.58
1.57
1.47
1.46
1.46
1.45
1.43
1.42
1.41
1.40
1.39
1.37
1.36
1.36
1.35
1.34
1.34
1.33
1.31
1.30
1.28
0.94
0.93
0.92
F
N
O
6
4n
1.00
2.03
1.00
2.05
1.12
1.02
2.04
2.11
2.14
2.16
2.18
2.17
4.10
3.15
3.01
f1 (ppm)

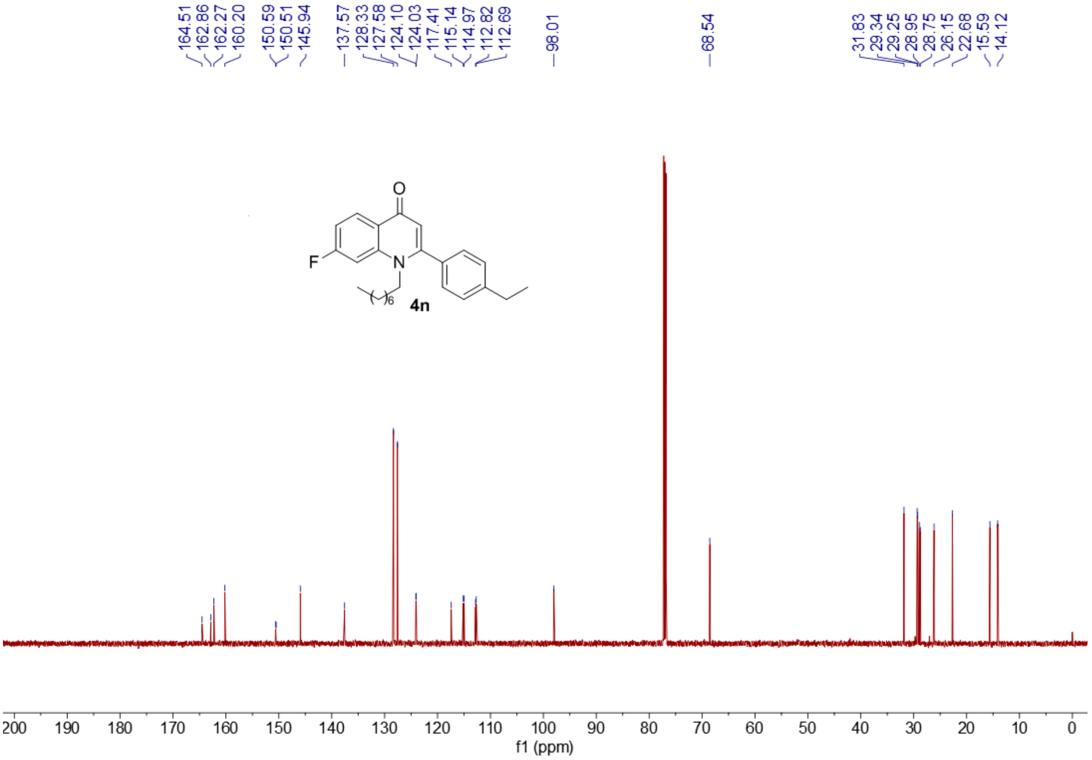
164.51
162.86
162.27
160.20
150.59
150.51
145.94
137.57
128.33
127.58
124.10
124.03
117.41
115.14
114.97
112.82
112.69
98.01
68.54
31.83
29.34
29.25
28.95
28.75
26.15
22.68
15.59
14.12
F
N
O
6
4n
f1 (ppm)

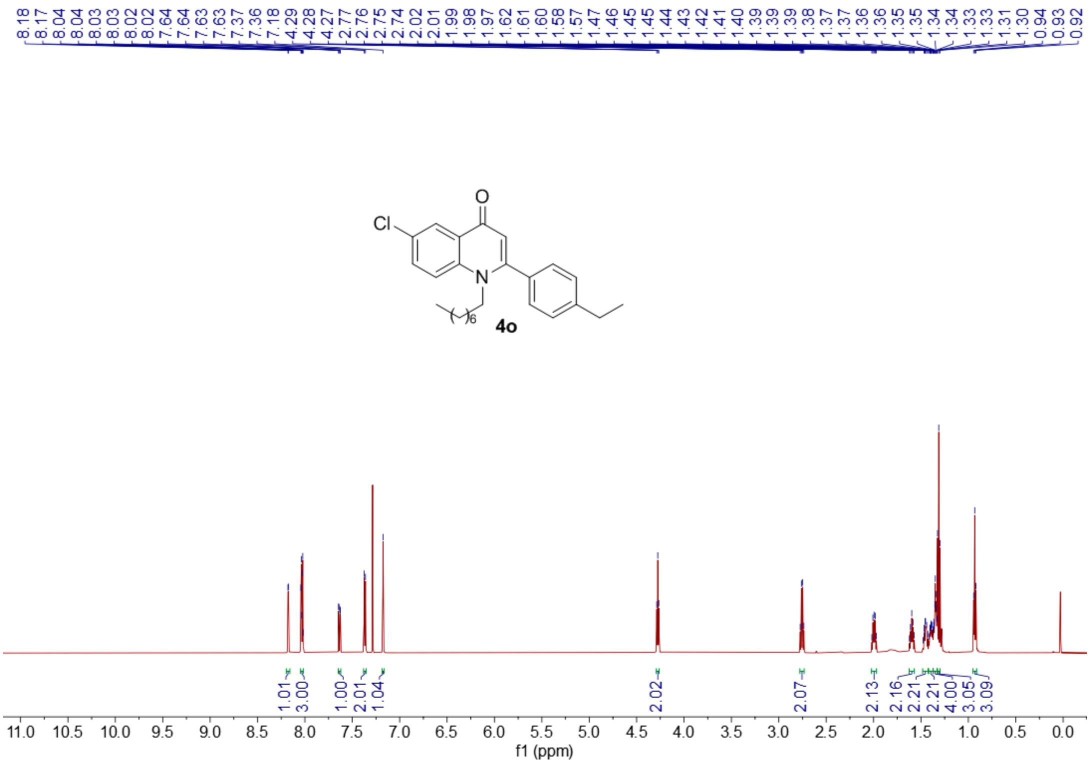

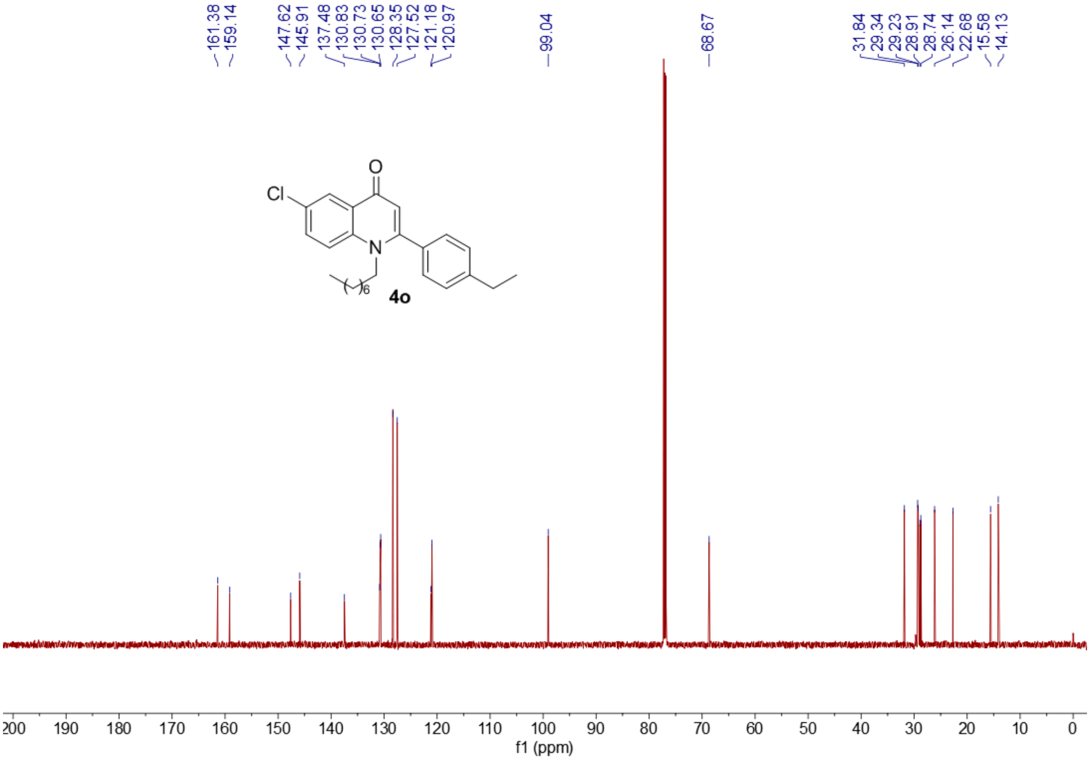

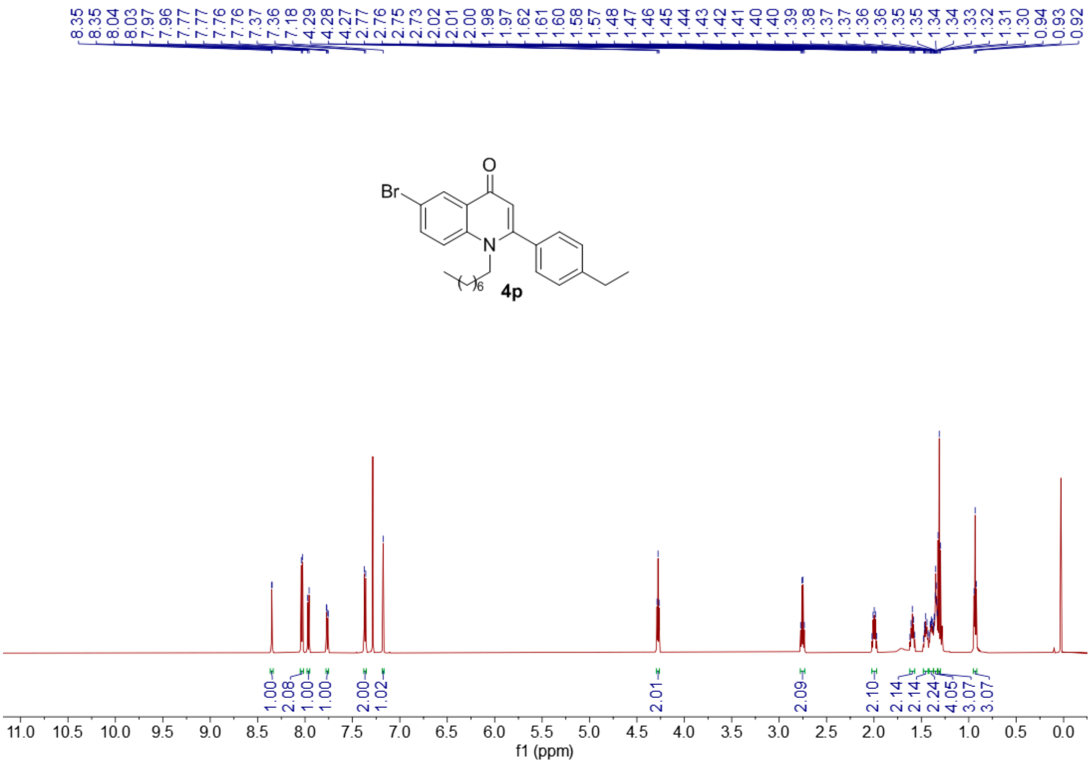
8.35 8.35 8.04 8.03 7.97 7.96 7.77 7.77 7.76 7.37 7.36 7.18 4.29 4.28 4.27 2.77 2.76 2.75 2.73 2.02 2.01 2.00 1.98 1.97 1.62 1.61 1.60 1.58 1.57 1.48 1.47 1.46 1.45 1.44 1.43 1.42 1.41 1.40 1.40 1.39 1.38 1.37 1.37 1.36 1.36 1.35 1.34 1.34 1.33 1.32 1.31 1.30 0.94 0.93 0.92
Br
O
N
6
4p
1.00 2.08 1.00 1.00 2.00 1.02 2.01 2.09 2.10 2.14 2.14 2.24 4.05 3.07 3.07
11.0 10.5 10.0 9.5 9.0 8.5 8.0 7.5 7.0 6.5 6.0 5.5 5.0 4.5 4.0 3.5 3.0 2.5 2.0 1.5 1.0 0.5 0.0
f1 (ppm)

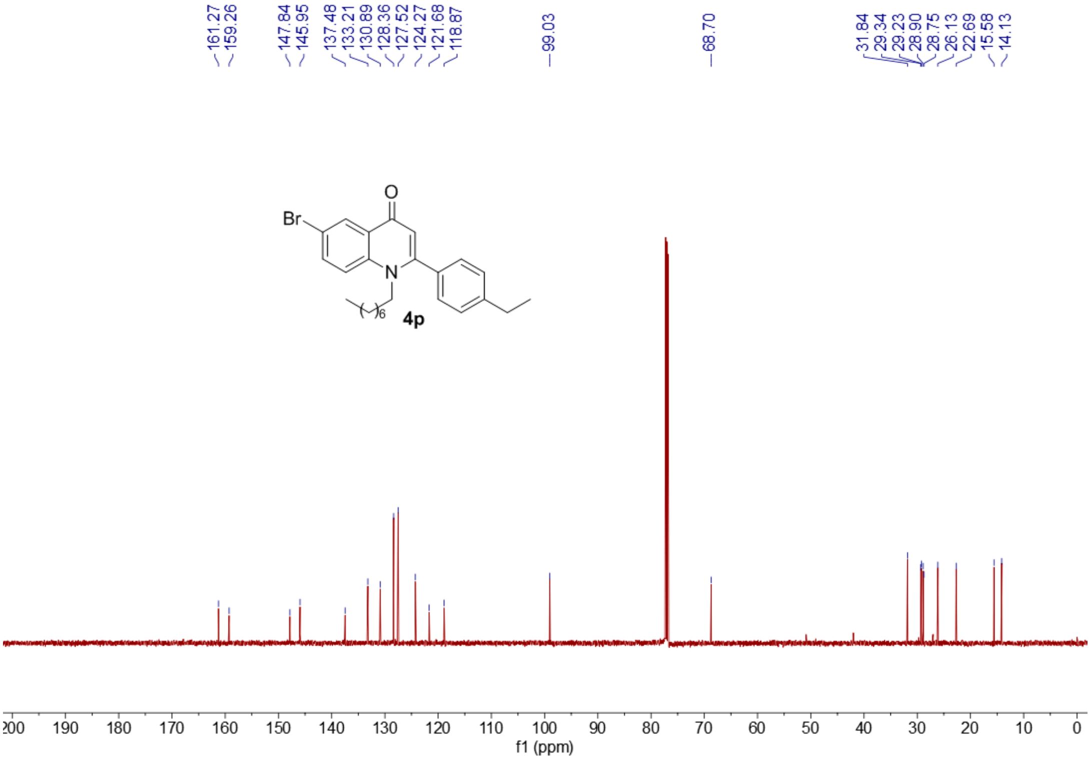
161.27 159.26 147.84 145.95 137.48 133.21 130.89 128.36 127.52 124.27 121.68 118.87 99.03 68.70 31.84 29.34 29.23 28.90 28.75 26.13 22.69 15.58 14.13
Br
O
N
6
4p
200 190 180 170 160 150 140 130 120 110 100 90 80 70 60 50 40 30 20 10 0
f1 (ppm)

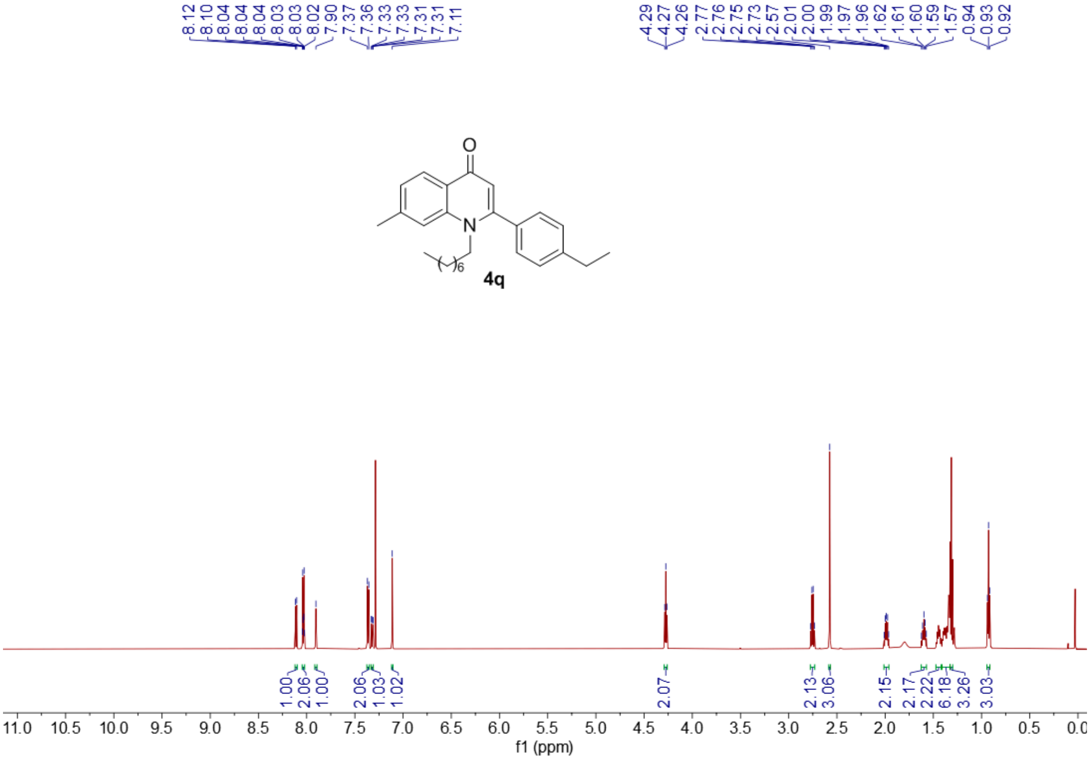
8.12 8.10 8.04 8.04 8.04 8.03 8.03 8.02 7.90 7.37 7.36 7.33 7.33 7.31 7.31 7.11
4.29 4.27 4.26 2.77 2.76 2.75 2.73 2.57 2.01 2.00 1.99 1.97 1.96 1.62 1.61 1.60 1.59 1.57 0.94 0.93 0.92
O
N
6
4q
1.00 2.06 1.00 2.06 1.03 1.02 2.07 2.13 3.06 2.15 2.17 2.22 6.18 3.26 3.03
11.0 10.5 10.0 9.5 9.0 8.5 8.0 7.5 7.0 6.5 6.0 5.5 5.0 4.5 4.0 3.5 3.0 2.5 2.0 1.5 1.0 0.5 0.0
f1 (ppm)

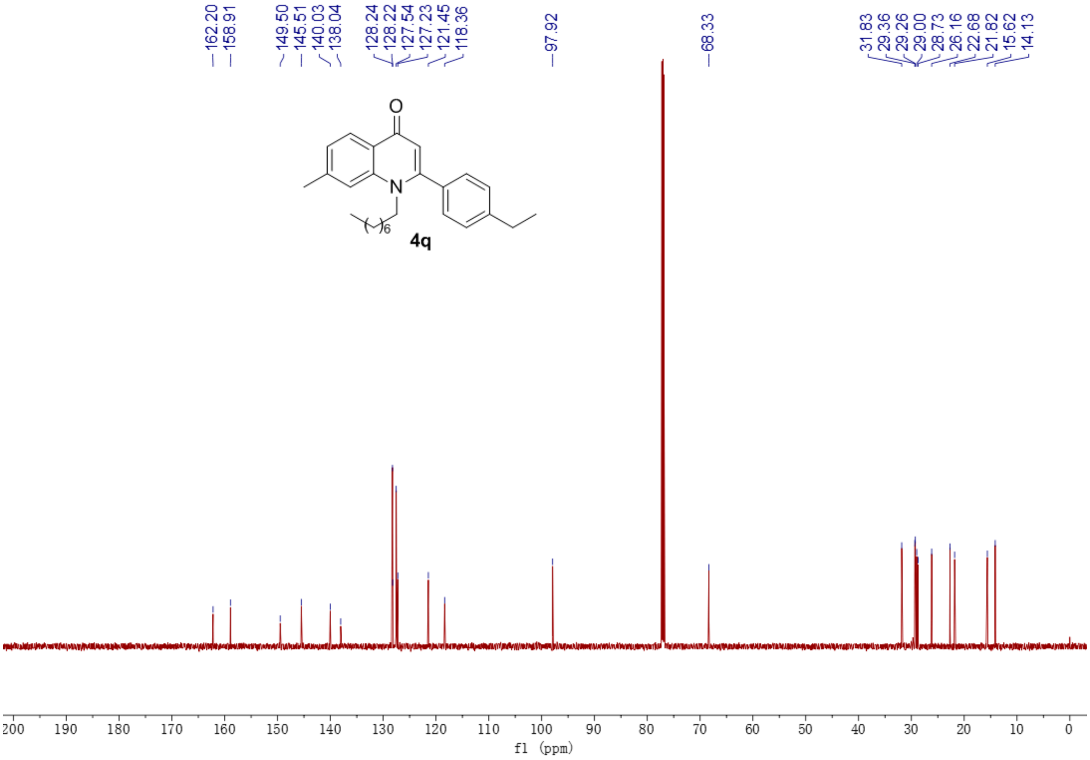
162.20 158.91 149.50 145.51 140.03 138.04 128.24 128.22 127.54 127.23 121.45 118.36 97.92 68.33 31.83 29.36 29.26 29.00 28.73 26.16 22.68 21.82 15.62 14.13
O
N
6
4q
200 190 180 170 160 150 140 130 120 110 100 90 80 70 60 50 40 30 20 10 0
f1 (ppm)

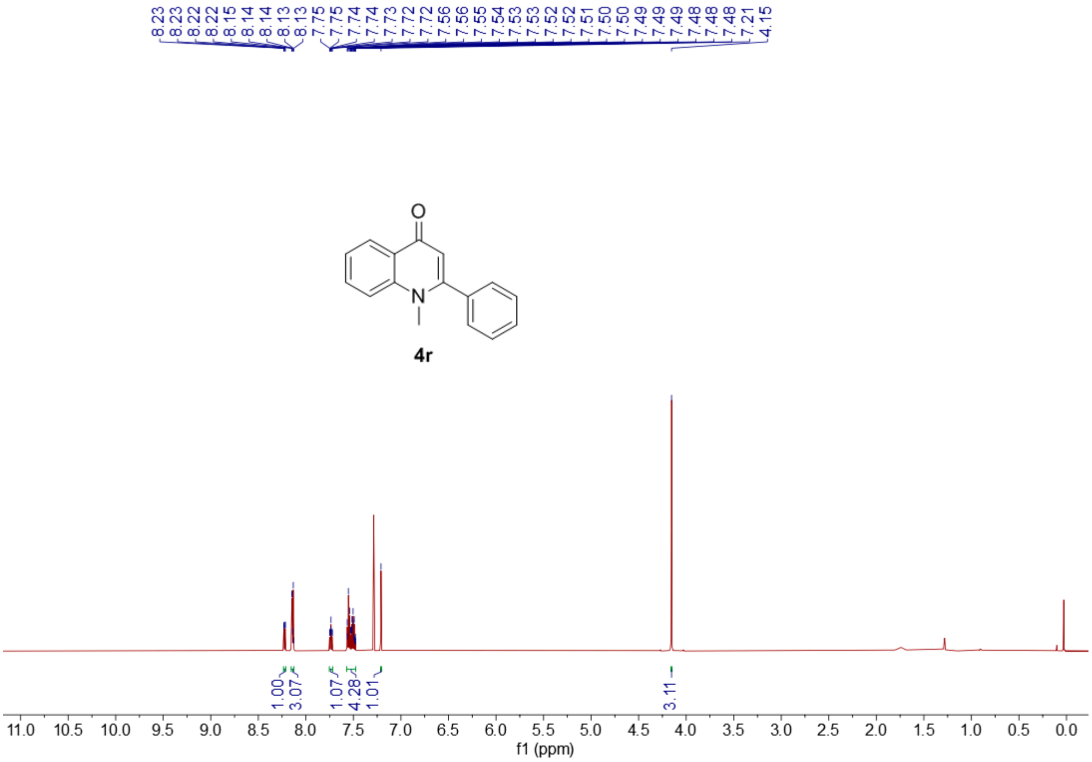
8.23 8.23 8.22 8.22 8.15 8.14 8.14 8.13 8.13 7.75 7.75 7.74 7.74 7.73 7.72 7.72 7.56 7.56 7.55 7.54 7.53 7.53 7.52 7.52 7.51 7.50 7.50 7.49 7.49 7.49 7.48 7.48 7.48 7.21 4.15
4r
1.00 3.07 1.07 4.28 1.01 3.11
f1 (ppm)

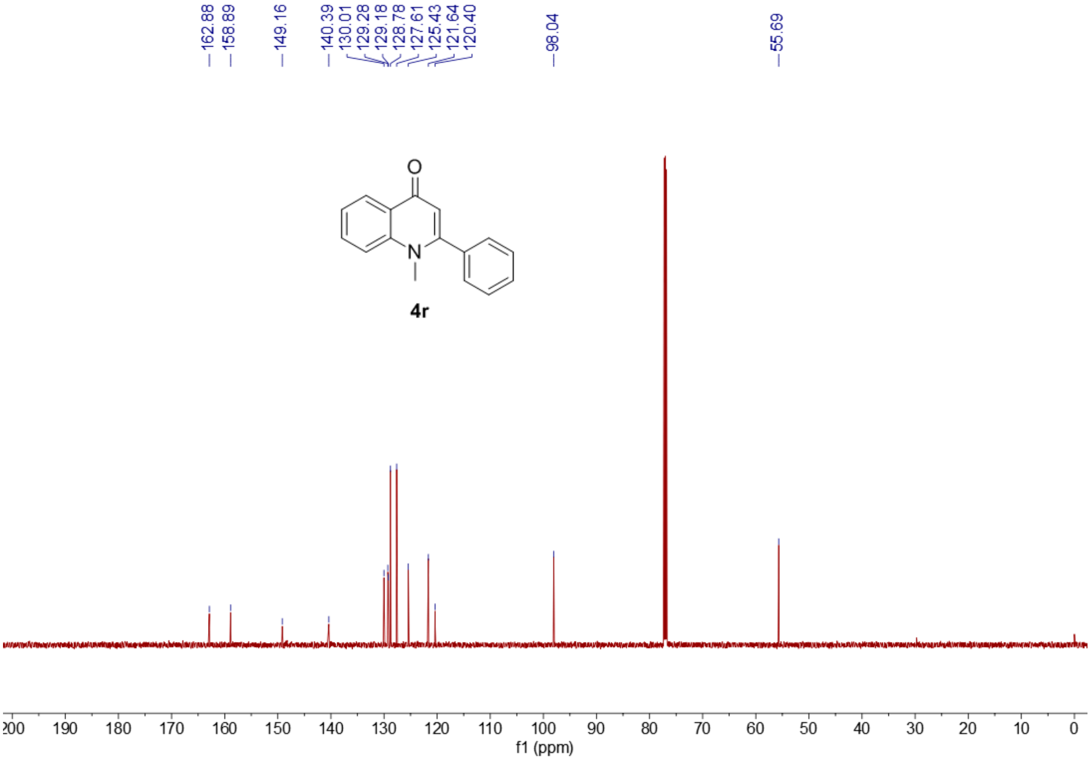
162.88 158.89 149.16 140.39 130.01 129.28 129.18 128.78 127.61 125.43 121.64 120.40 98.04 55.69
4r
f1 (ppm)

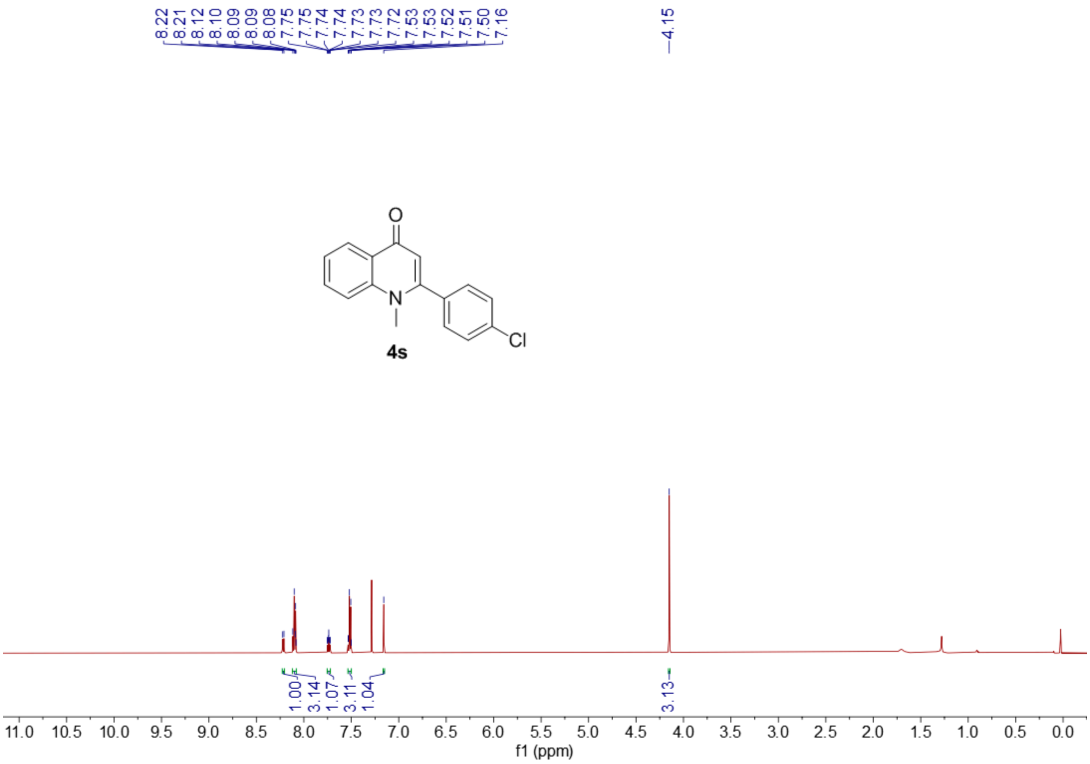

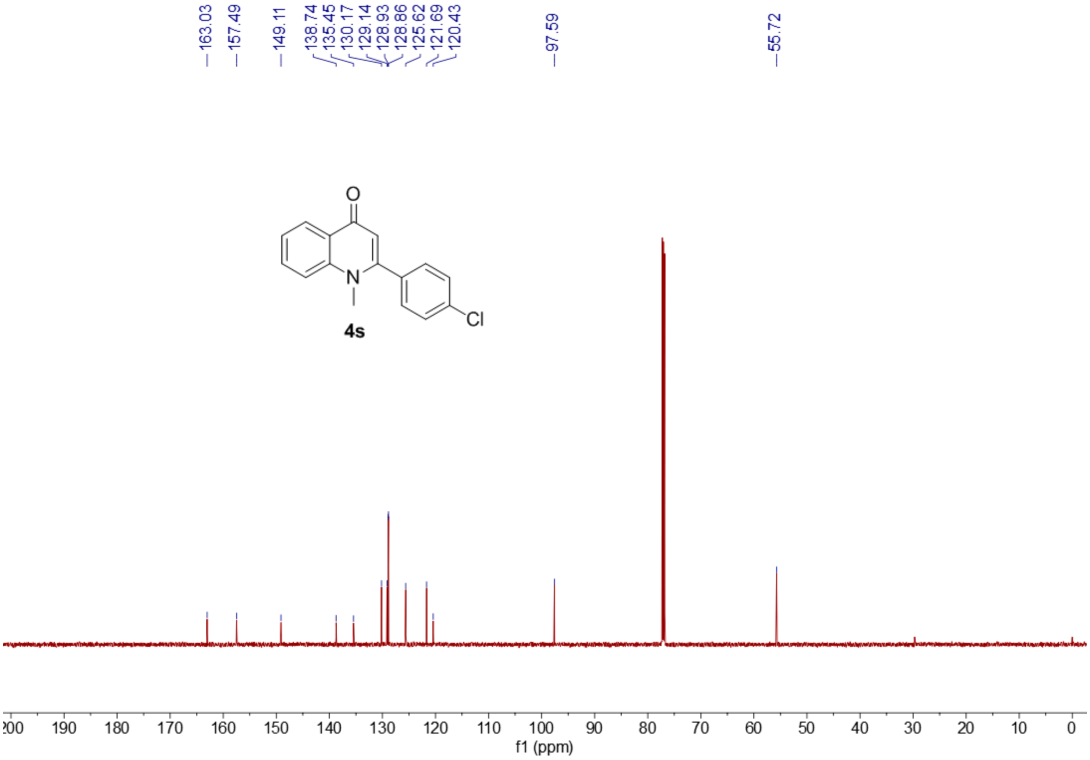

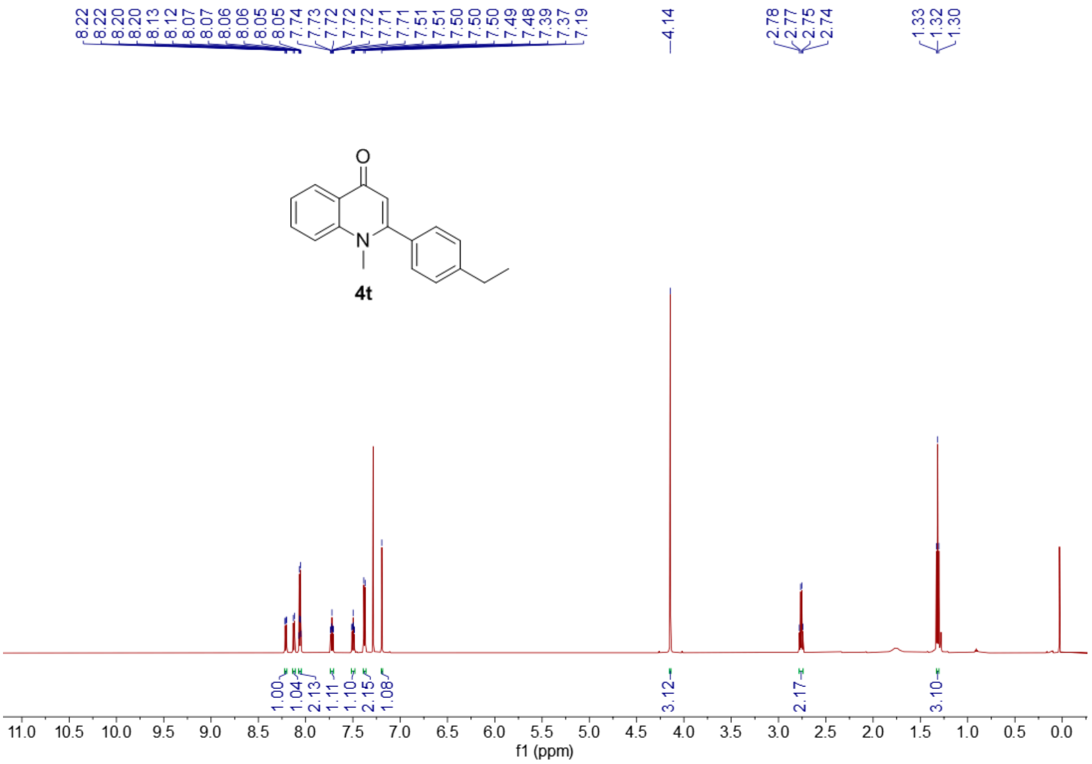
8.22
8.22
8.20
8.20
8.13
8.12
8.07
8.06
8.06
8.05
8.05
7.74
7.73
7.72
7.72
7.72
7.71
7.71
7.51
7.51
7.50
7.50
7.49
7.48
7.39
7.37
7.19
4.14
2.78
2.77
2.75
2.74
1.33
1.32
1.30
O
N
4t
1.00
1.04
2.13
1.11
1.10
2.15
1.08
3.12
2.17
3.10
11.0 10.5 10.0 9.5 9.0 8.5 8.0 7.5 7.0 6.5 6.0 5.5 5.0 4.5 4.0 3.5 3.0 2.5 2.0 1.5 1.0 0.5 0.0
f1 (ppm)

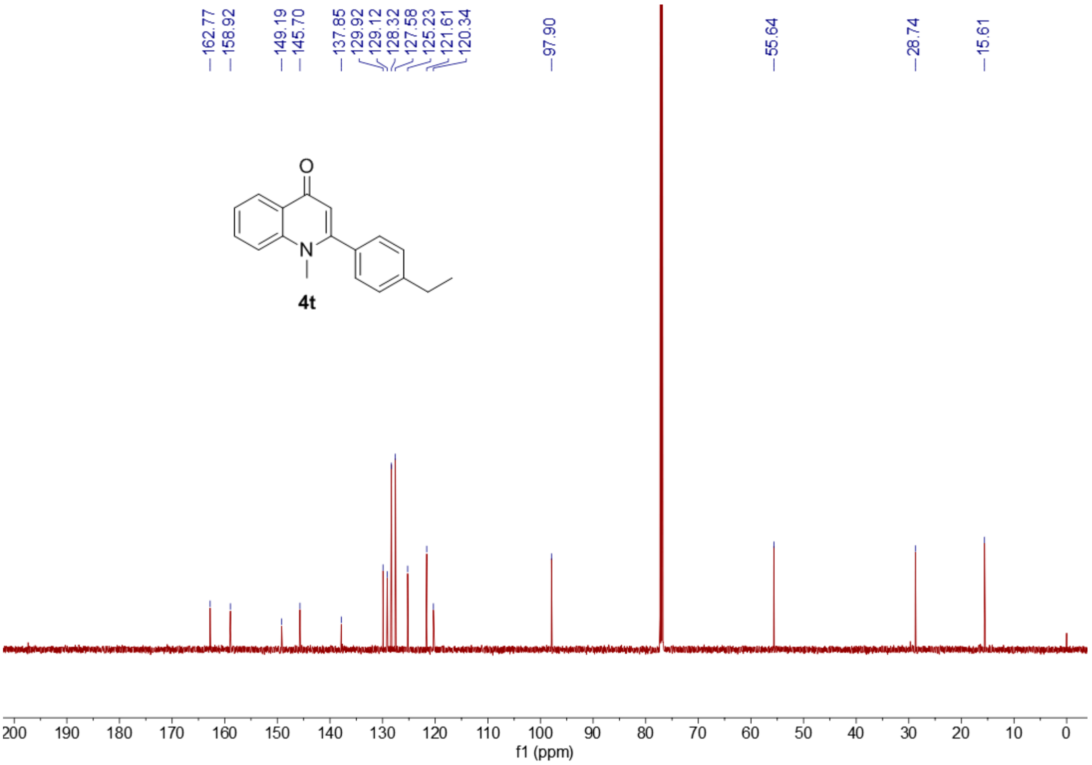
162.77
158.92
149.19
145.70
137.85
129.92
129.12
128.32
127.58
125.23
121.61
120.34
97.90
55.64
28.74
15.61
O
N
4t
200 190 180 170 160 150 140 130 120 110 100 90 80 70 60 50 40 30 20 10 0
f1 (ppm)

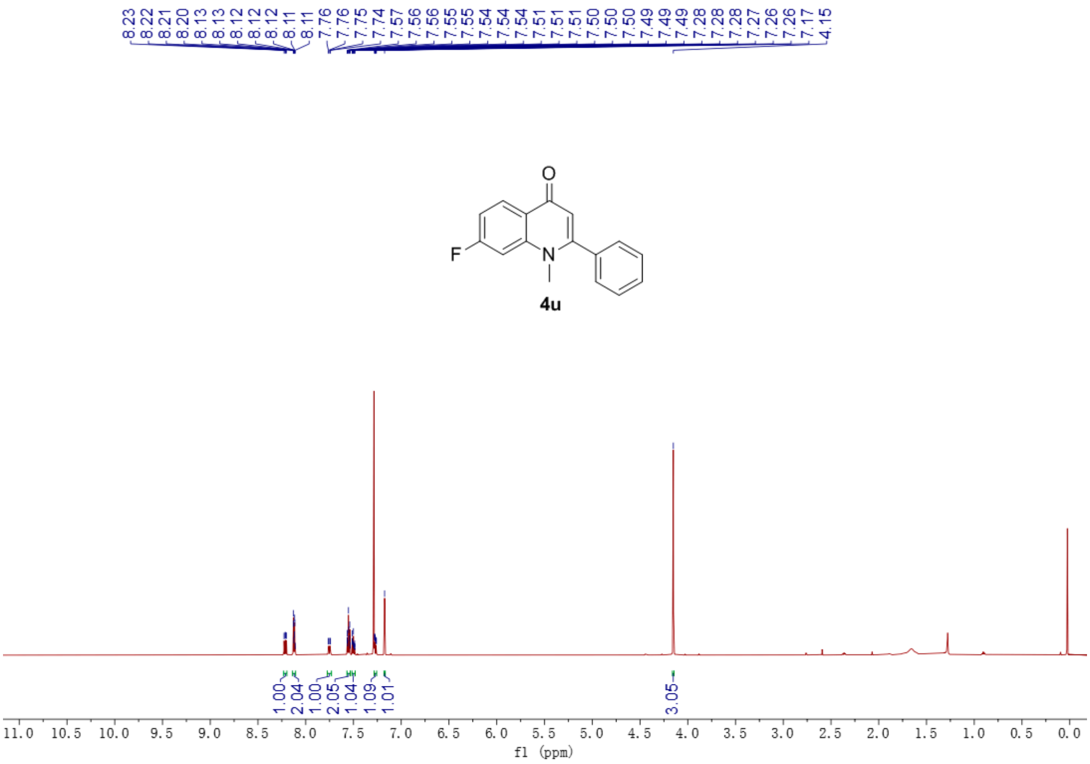
8.23 8.22 8.21 8.20 8.13 8.13 8.12 8.12 8.12 8.11 8.11 7.76 7.76 7.75 7.74 7.57 7.56 7.56 7.55 7.55 7.54 7.54 7.54 7.51 7.51 7.51 7.50 7.50 7.50 7.49 7.49 7.49 7.28 7.28 7.28 7.27 7.26 7.26 7.17 4.15
O
F
N
4u
1.00 2.04 1.00 2.05 1.04 1.09 1.01
3.05
11.0 10.5 10.0 9.5 9.0 8.5 8.0 7.5 7.0 6.5 6.0 5.5 5.0 4.5 4.0 3.5 3.0 2.5 2.0 1.5 1.0 0.5 0.0
f1 (ppm)

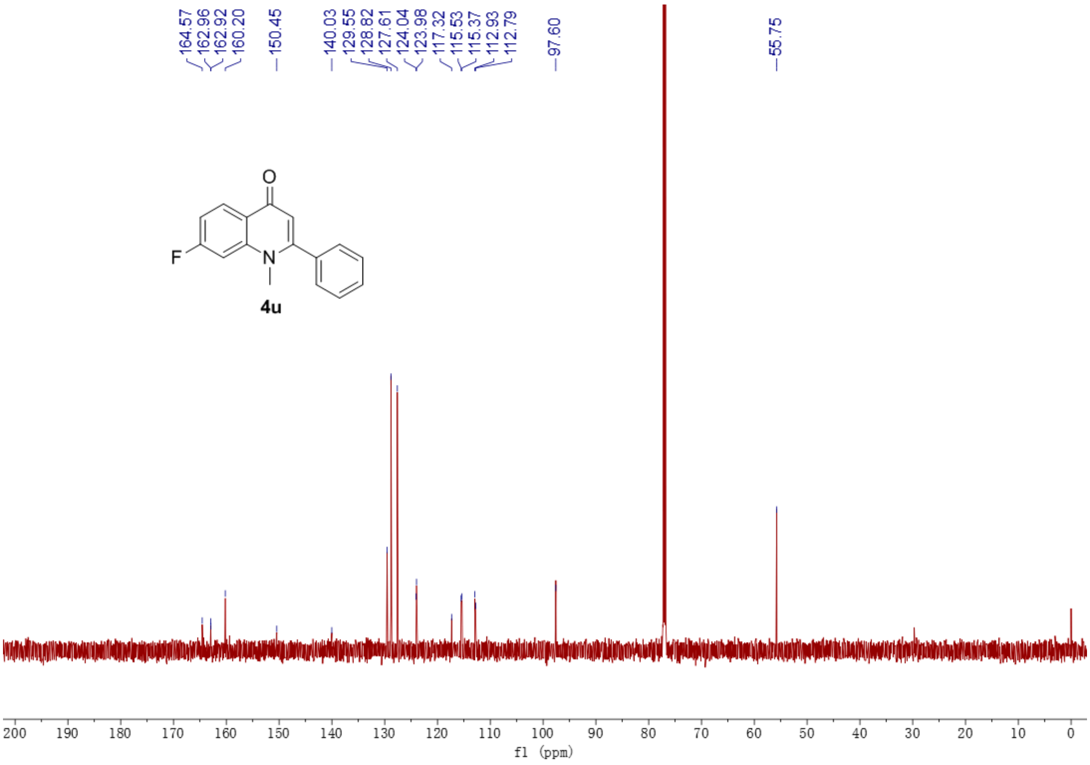
164.57 162.96 162.92 160.20 150.45 140.03 129.55 128.82 127.61 124.04 123.98 117.32 115.53 115.37 112.93 112.79 97.60 55.75
O
F
N
4u
200 190 180 170 160 150 140 130 120 110 100 90 80 70 60 50 40 30 20 10 0
f1 (ppm)

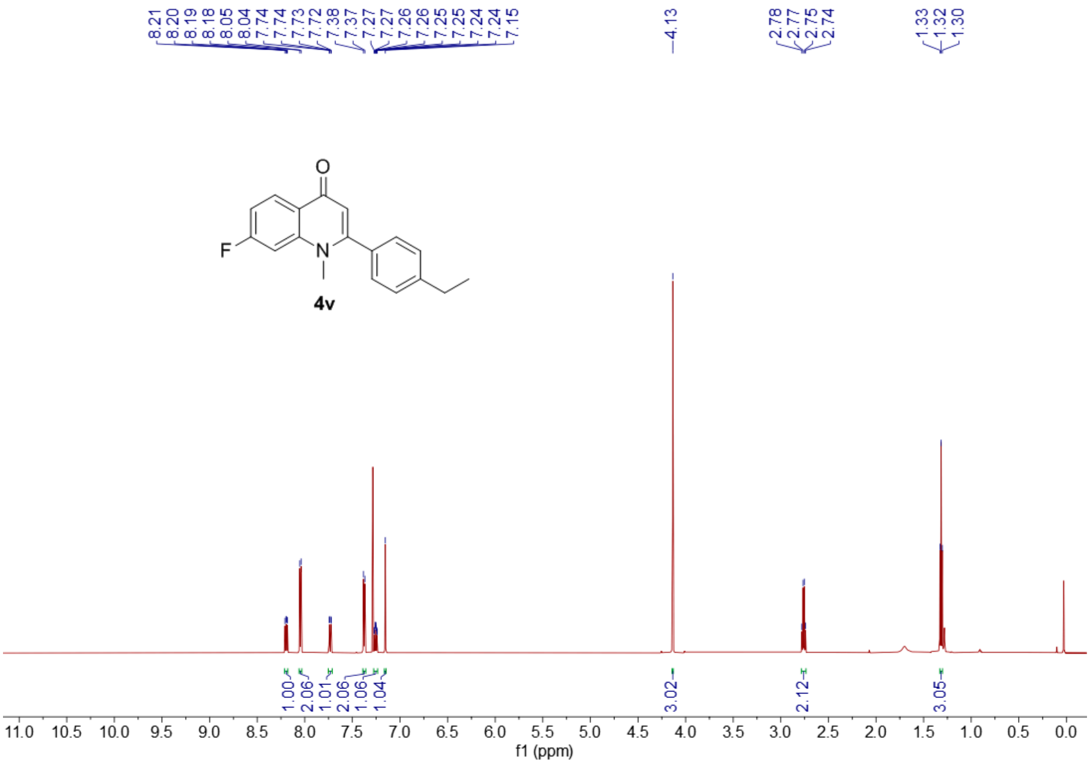

8.21
8.20
8.19
8.18
8.05
8.04
7.74
7.74
7.73
7.72
7.38
7.37
7.27
7.27
7.26
7.26
7.25
7.25
7.24
7.24
7.15
4.13
2.78
2.77
2.75
2.74
1.33
1.32
1.30
F
N
O
4v
1.00
2.06
1.01
2.06
1.06
1.04
3.02
2.12
3.05
11.0 10.5 10.0 9.5 9.0 8.5 8.0 7.5 7.0 6.5 6.0 5.5 5.0 4.5 4.0 3.5 3.0 2.5 2.0 1.5 1.0 0.5 0.0
f1 (ppm)


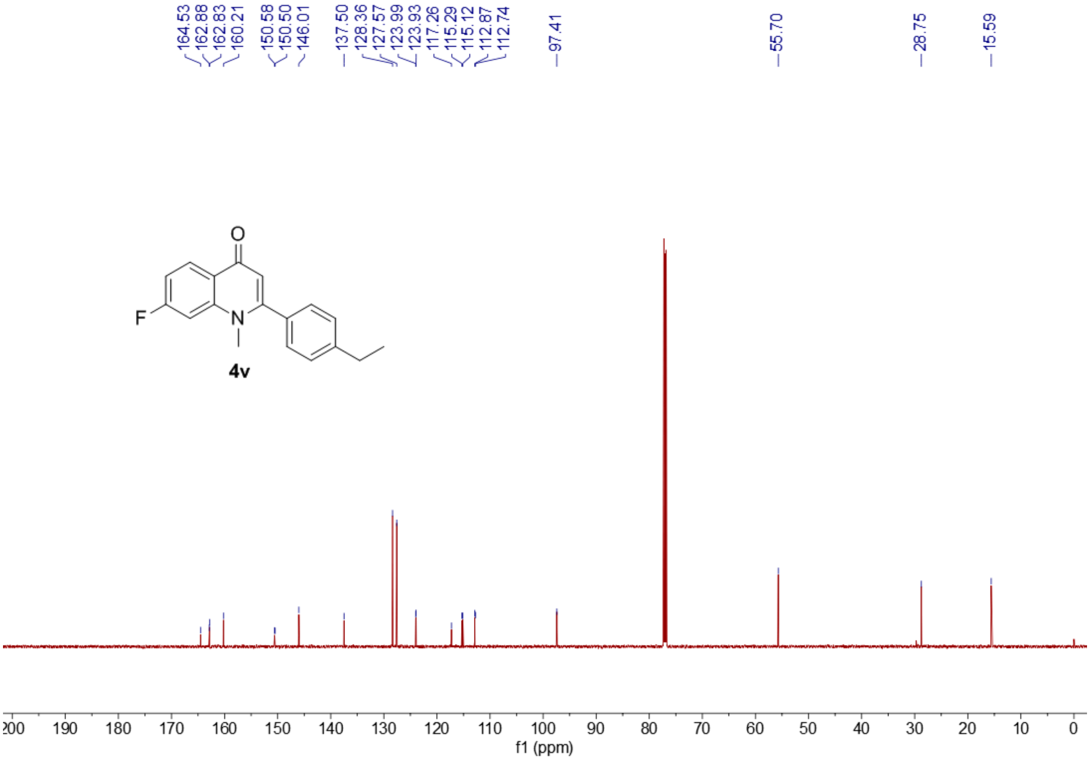

164.53
162.88
162.83
160.21
150.58
150.50
146.01
137.50
128.36
127.57
123.99
123.93
117.26
115.29
115.12
112.87
112.74
97.41
55.70
28.75
15.59
F
N
O
4v
200 190 180 170 160 150 140 130 120 110 100 90 80 70 60 50 40 30 20 10 0
f1 (ppm)

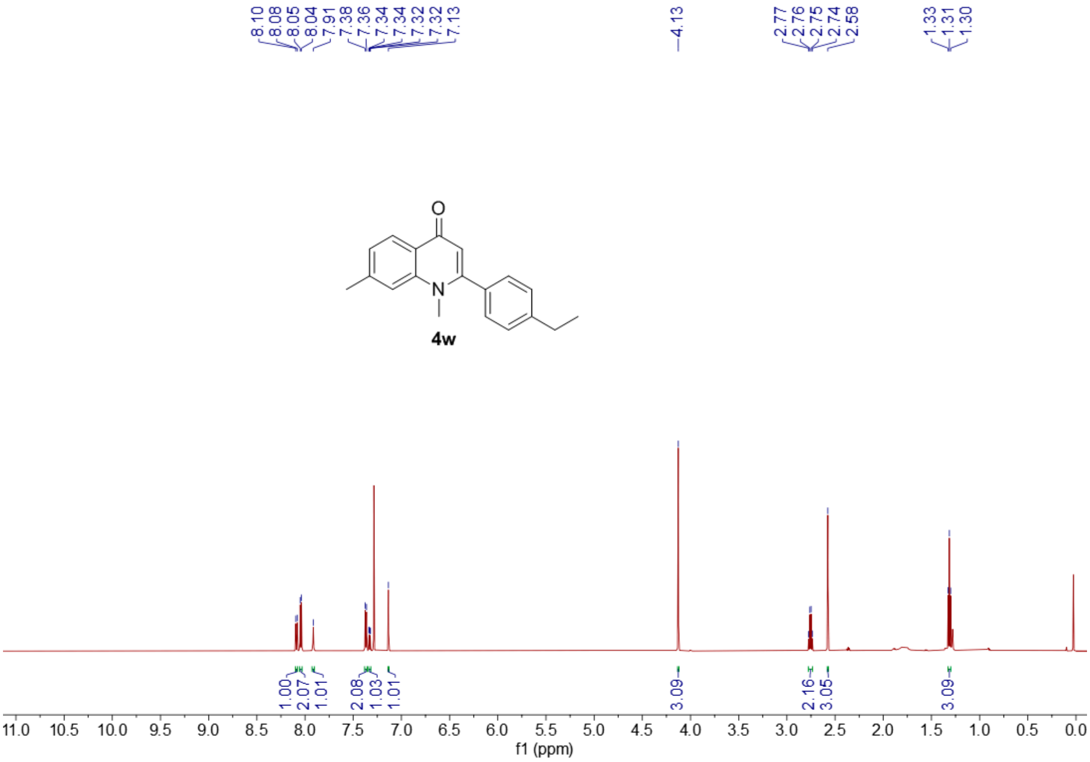
8.10
8.08
8.05
8.04
7.91
7.38
7.36
7.34
7.34
7.32
7.32
7.13
4.13
2.77
2.76
2.75
2.74
2.58
1.33
1.31
1.30
O
N
4w
1.00
2.07
1.01
2.08
1.03
1.01
3.09
2.16
3.05
3.09
11.0 10.5 10.0 9.5 9.0 8.5 8.0 7.5 7.0 6.5 6.0 5.5 5.0 4.5 4.0 3.5 3.0 2.5 2.0 1.5 1.0 0.5 0.0
f1 (ppm)

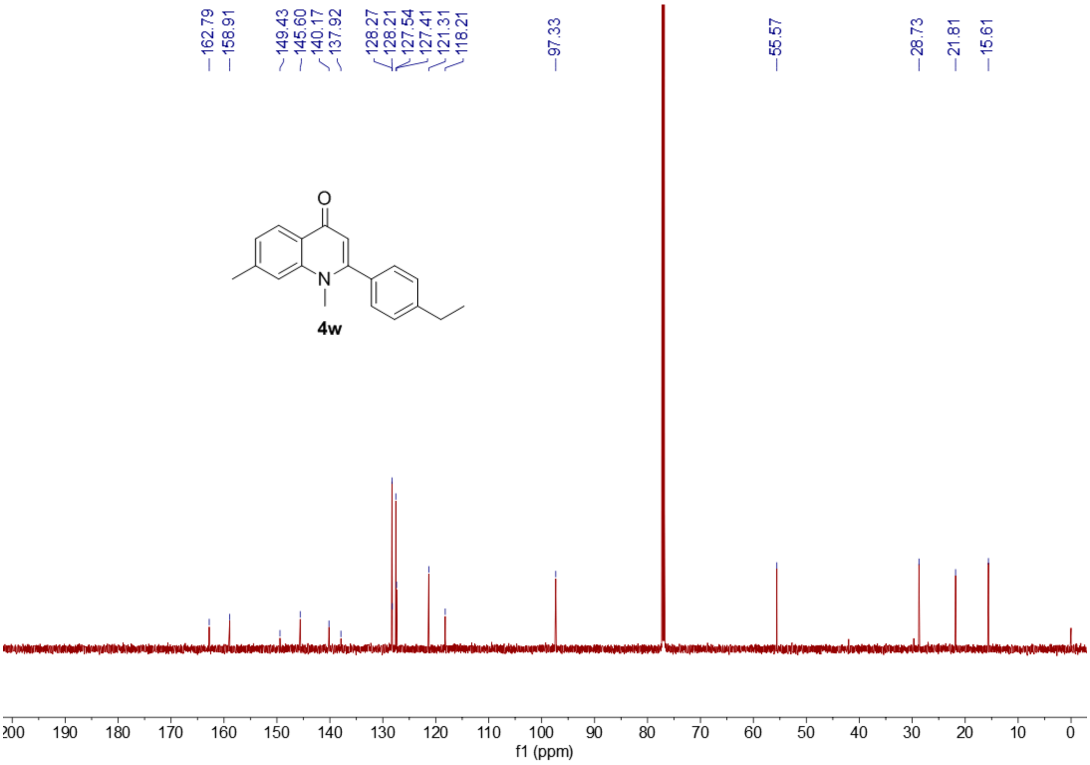
162.79
158.91
149.43
145.60
140.17
137.92
128.27
128.21
127.54
127.41
121.31
118.21
97.33
55.57
28.73
21.81
15.61
O
N
4w
200 190 180 170 160 150 140 130 120 110 100 90 80 70 60 50 40 30 20 10 0
f1 (ppm)

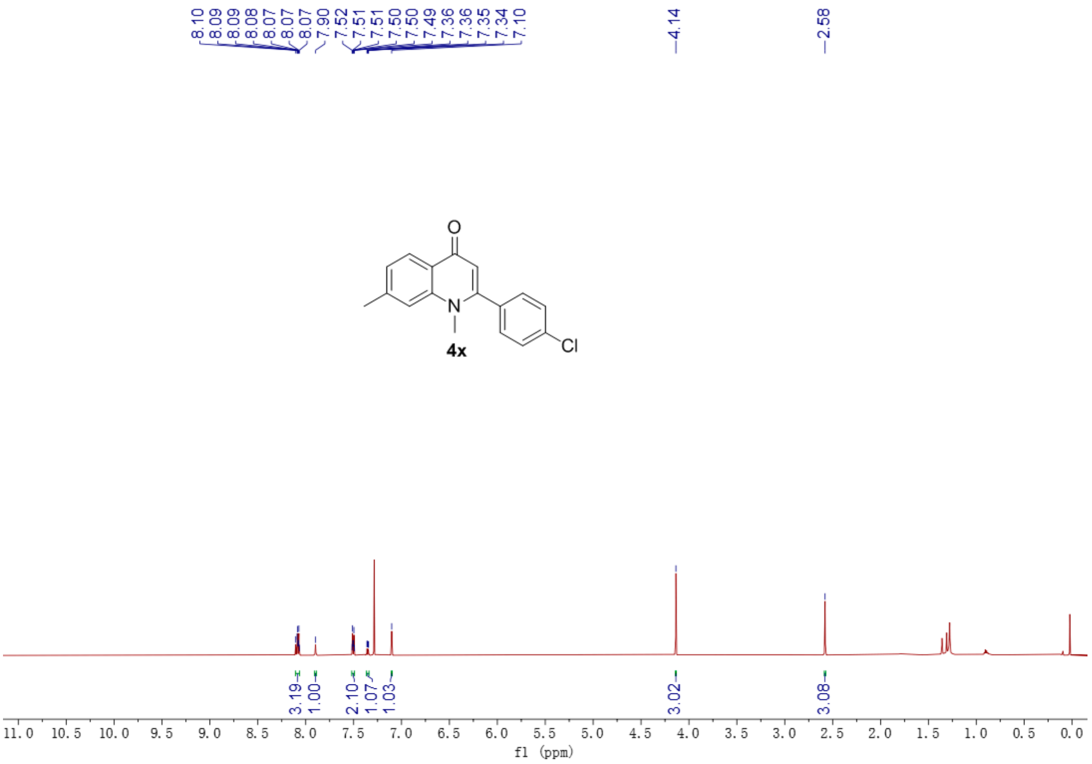
8.10
8.09
8.09
8.08
8.07
8.07
7.90
7.52
7.51
7.51
7.50
7.50
7.49
7.36
7.36
7.35
7.34
7.10
4.14
2.58
4x
Cl
3.19
1.00
2.10
1.07
1.03
3.02
3.08
f1 (ppm)

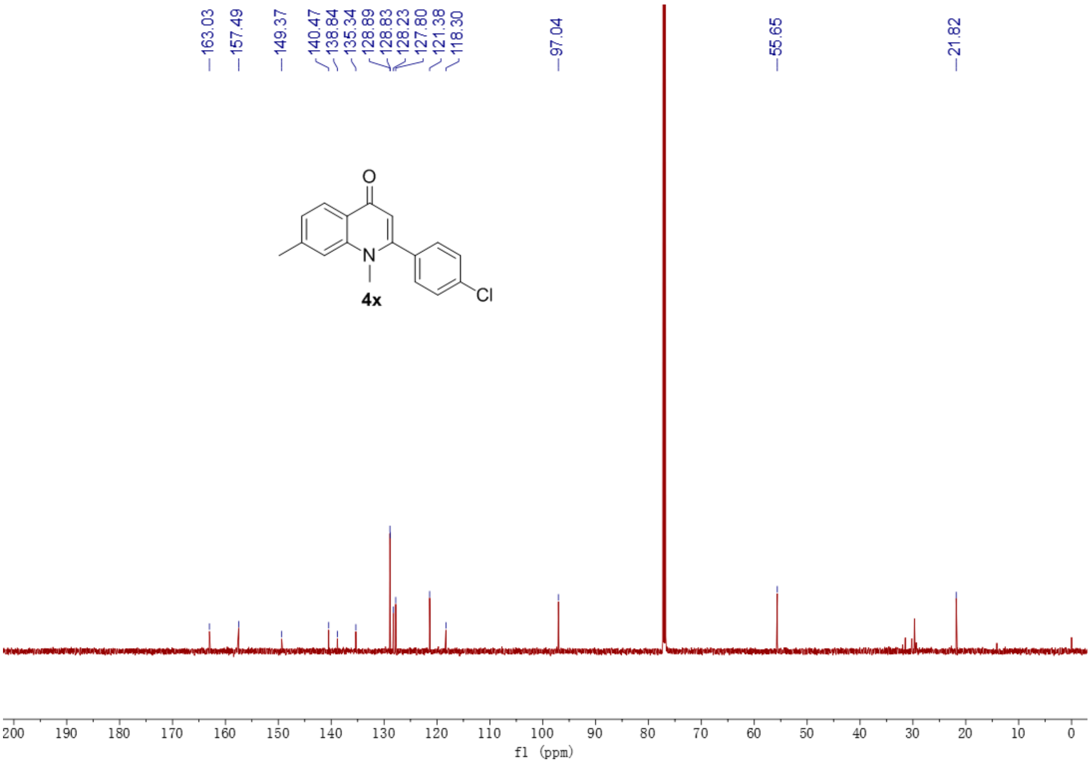
163.03
157.49
149.37
140.47
138.84
135.34
128.89
128.83
128.23
127.80
121.38
118.30
97.04
55.65
21.82
4x
Cl
f1 (ppm)

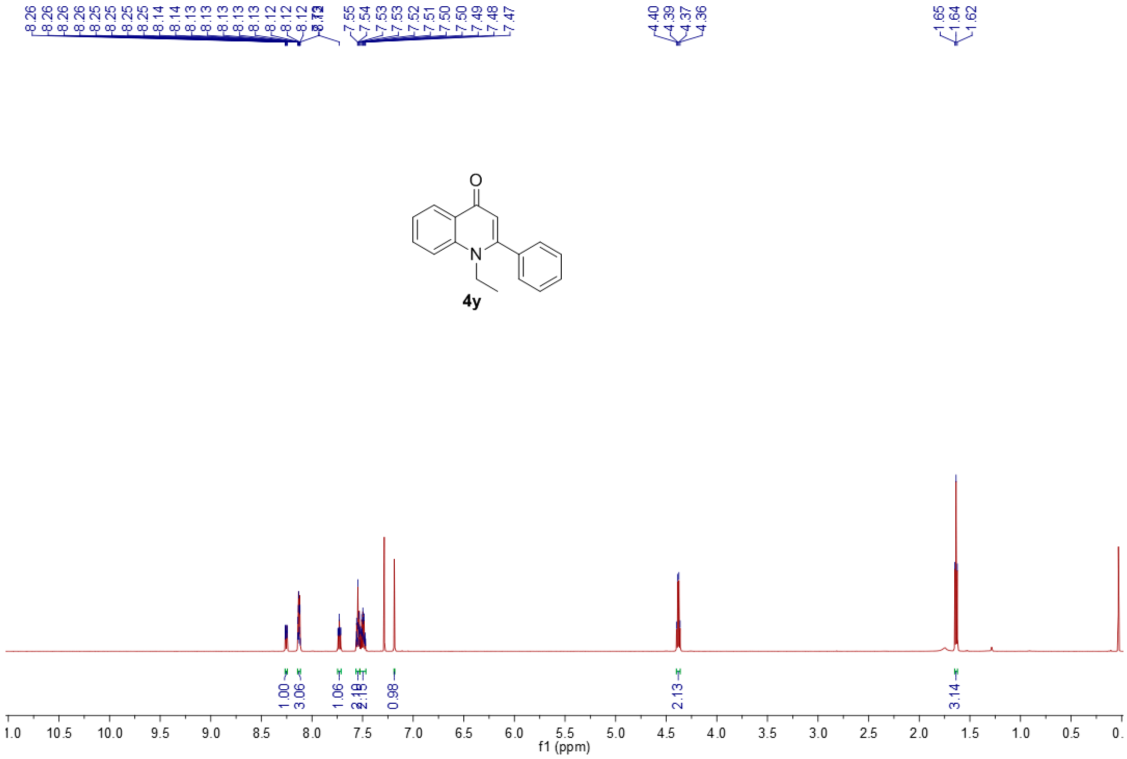
8.26 8.26 8.26 8.26 8.25 8.25 8.25 8.25 8.14 8.14 8.13 8.13 8.13 8.13 8.13 8.12 8.12 8.12 7.72
7.55 7.54 7.53 7.53 7.52 7.51 7.50 7.50 7.49 7.48 7.47
4.40 4.39 4.37 4.36
1.65 1.64 1.62
4y
1.00 3.06 1.06 2.10 2.15 0.98 2.13 3.14
f1 (ppm)

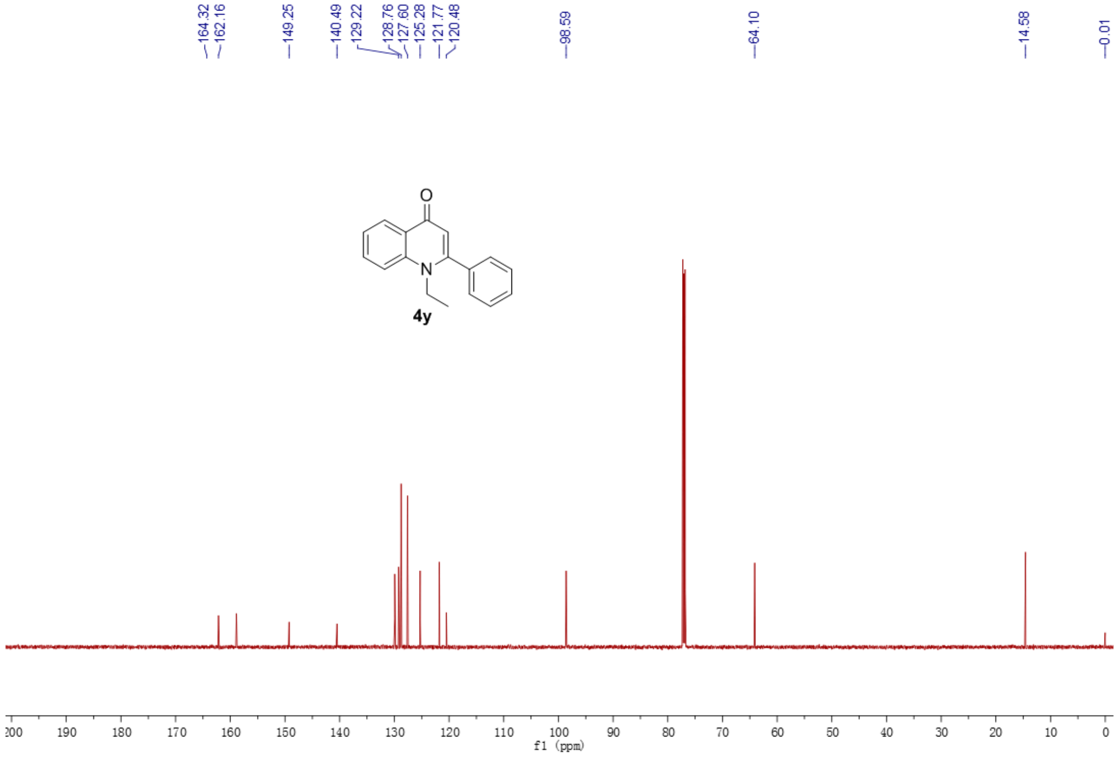
164.32 162.16 149.25 140.49 129.22 128.76 127.60 125.28 121.77 120.48 98.59 64.10 14.58 -0.01
4y
f1 (ppm)

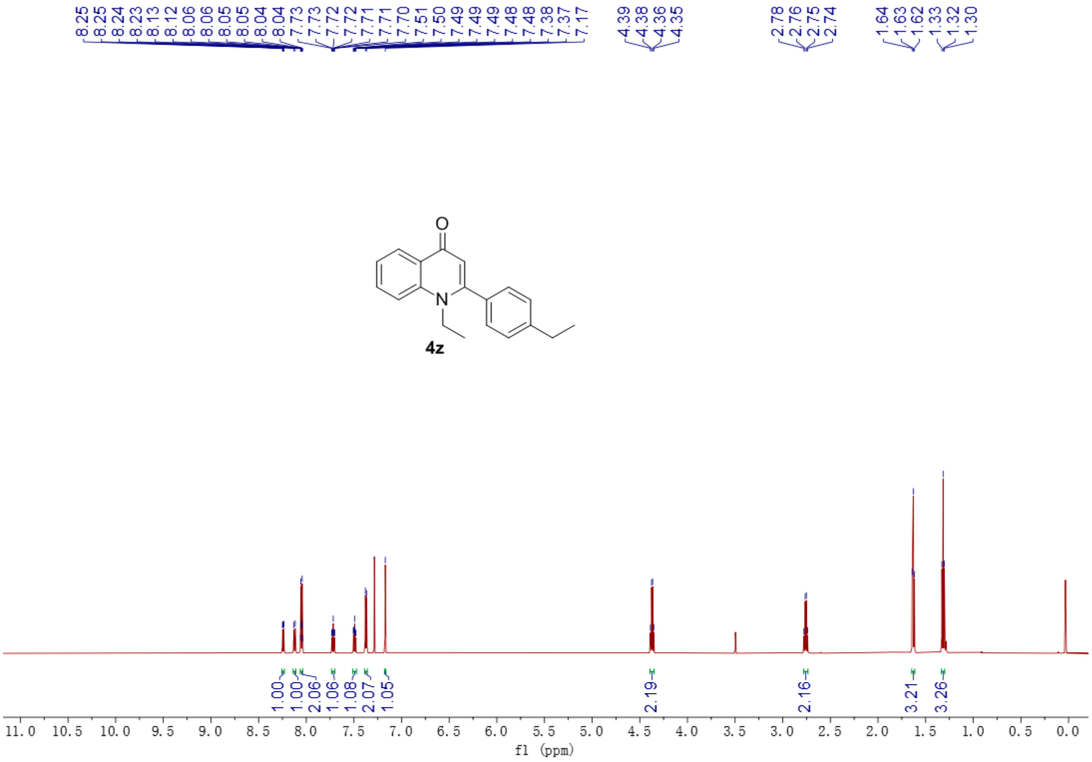
4z
f1 (ppm)

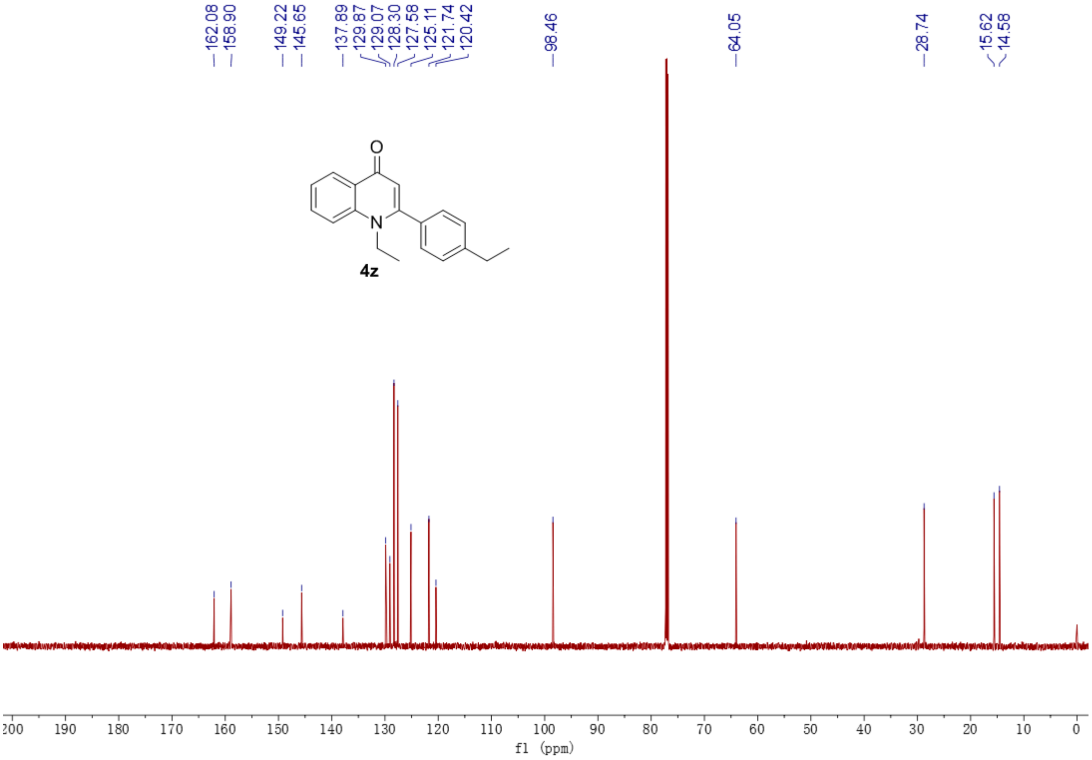
4z
f1 (ppm)

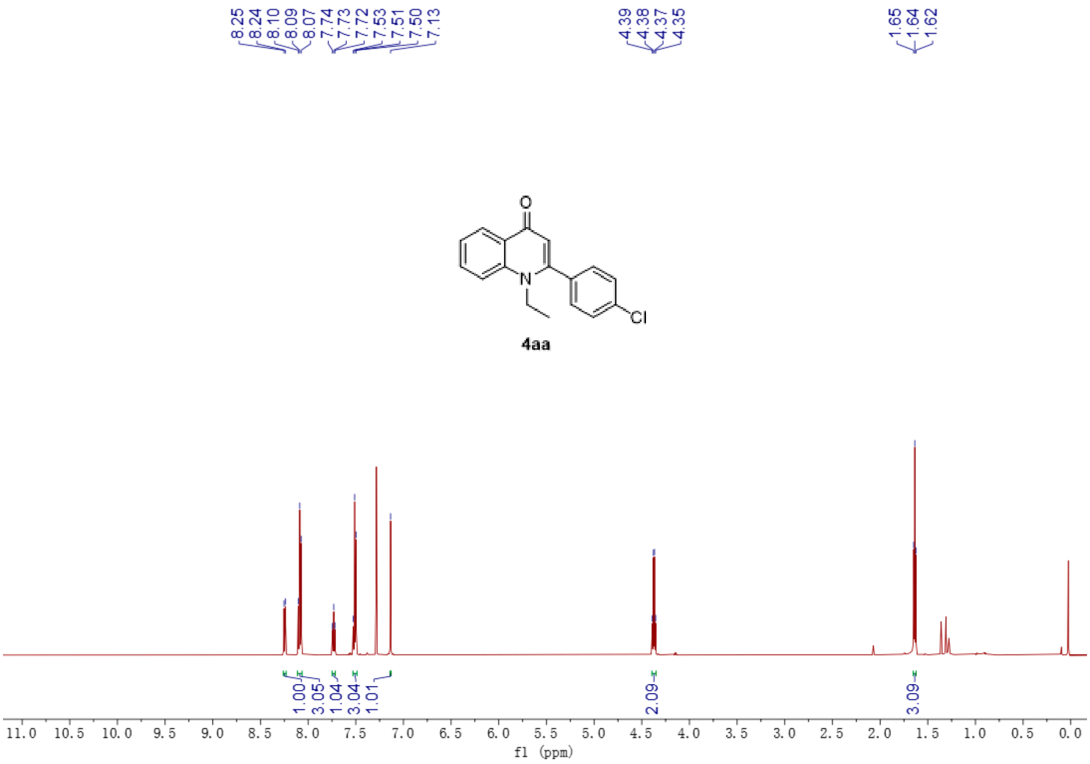
8.25 8.24 8.10 8.09 8.07 7.74 7.73 7.72 7.53 7.51 7.50 7.13
4.39 4.38 4.37 4.35
1.65 1.64 1.62
O
N
Cl
4aa
1.00 3.05 1.04 3.04 1.01
2.09
3.09
f1 (ppm)

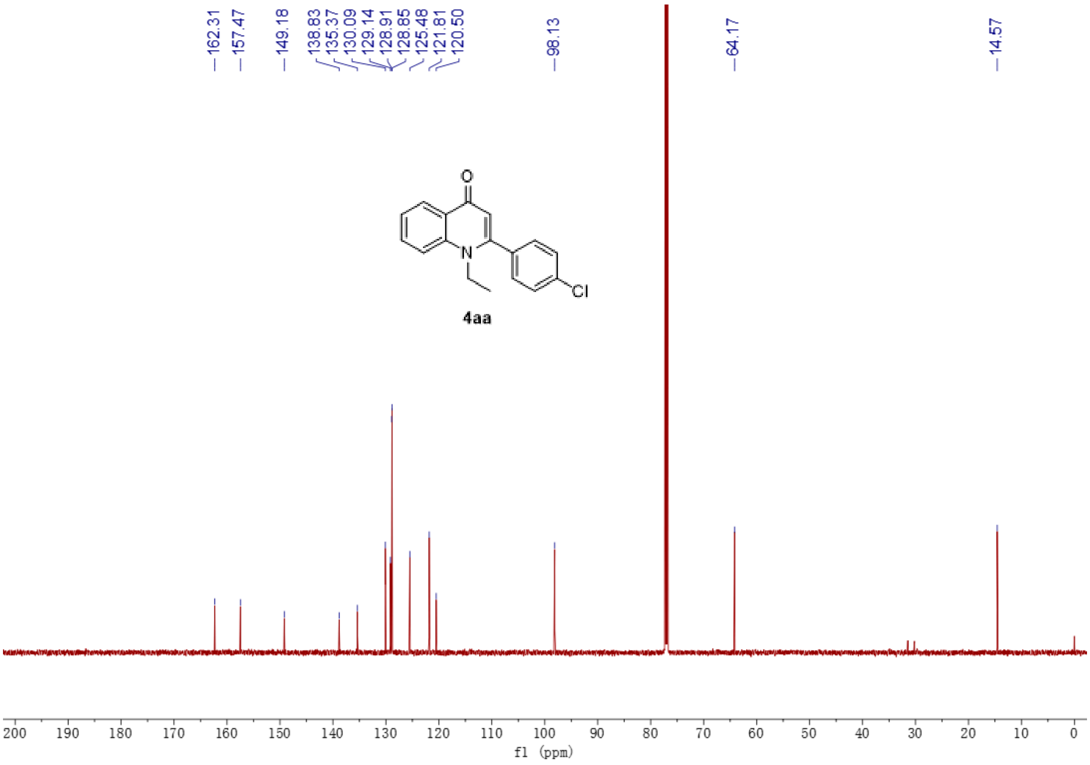
162.31 157.47 149.18 138.83 135.37 130.09 129.14 128.91 128.85 125.48 121.81 120.50
98.13
64.17
14.57
O
N
Cl
4aa
f1 (ppm)

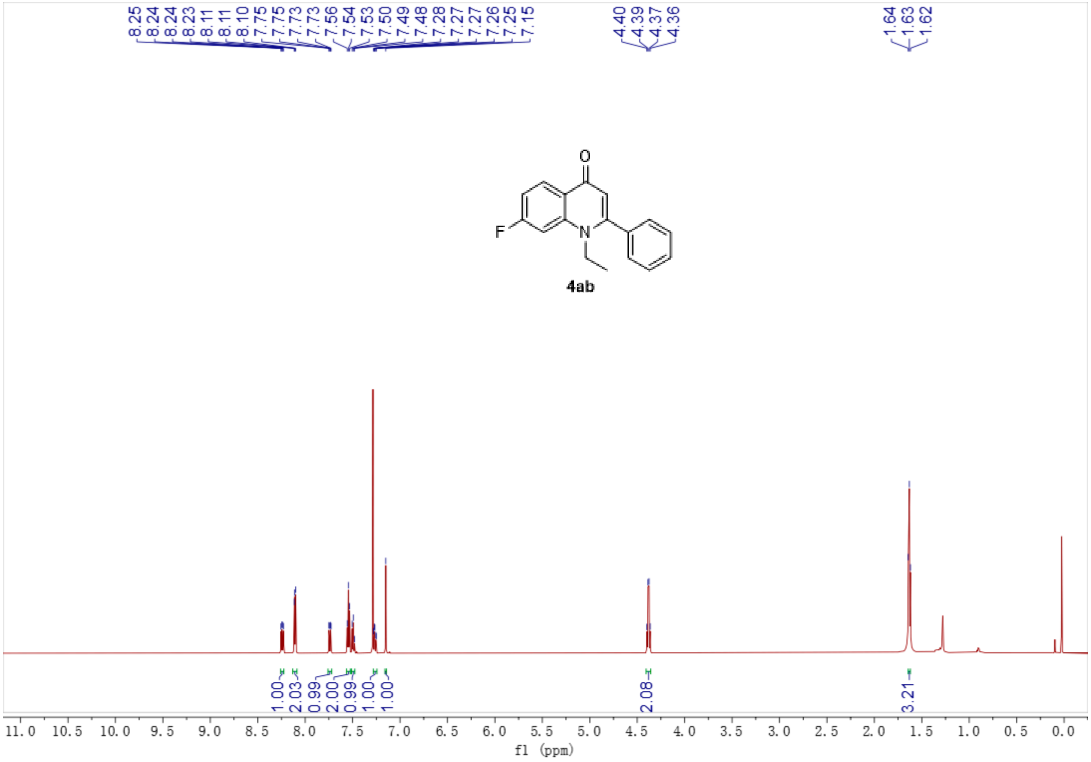
8.25 8.24 8.24 8.23 8.11 8.11 8.10 7.75 7.73 7.73 7.56 7.54 7.53 7.50 7.49 7.48 7.28 7.27 7.27 7.26 7.25 7.15
4.40 4.39 4.37 4.36
1.64 1.63 1.62
O
F
N
4ab
1.00 2.03 0.99 2.00 0.99 1.00 1.00
2.08
3.21
11.0 10.5 10.0 9.5 9.0 8.5 8.0 7.5 7.0 6.5 6.0 5.5 5.0 4.5 4.0 3.5 3.0 2.5 2.0 1.5 1.0 0.5 0.0
f1 (ppm)

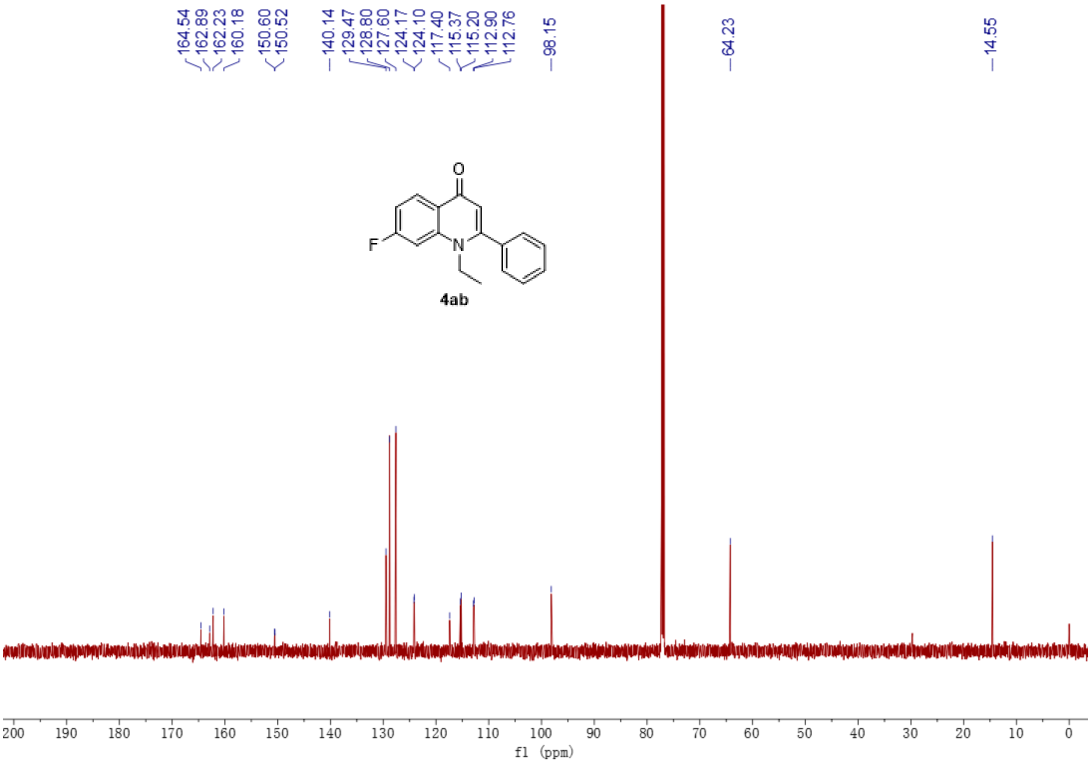
164.54 162.89 162.23 160.18 150.60 150.52
140.14 129.47 128.80 127.60 124.17 124.10 117.40 115.37 115.20 112.90 112.76
98.15
64.23
14.55
O
F
N
4ab
200 190 180 170 160 150 140 130 120 110 100 90 80 70 60 50 40 30 20 10 0
f1 (ppm)

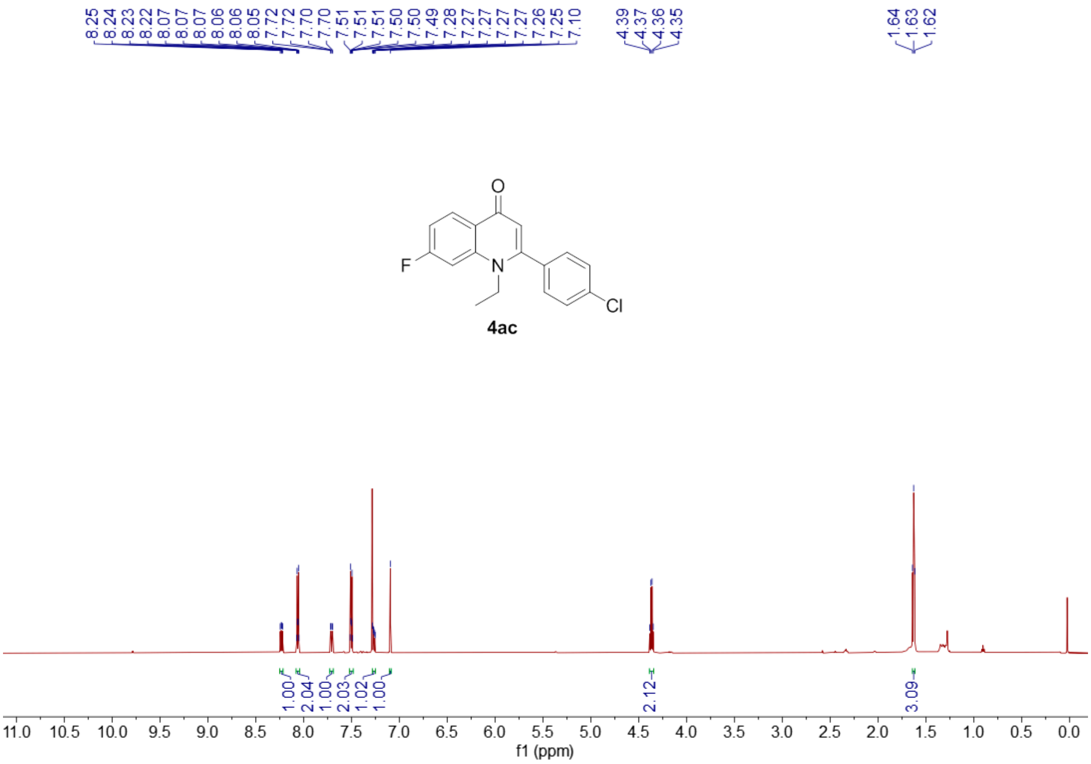
8.25
8.24
8.23
8.22
8.07
8.07
8.06
8.06
8.05
7.72
7.72
7.70
7.70
7.51
7.51
7.51
7.50
7.49
7.28
7.27
7.27
7.27
7.27
7.26
7.25
7.10
4.39
4.37
4.36
4.35
1.64
1.63
1.62
O
F
N
Cl
1.00
2.04
1.00
2.03
1.02
1.00
2.12
3.09
f1 (ppm)

4ac

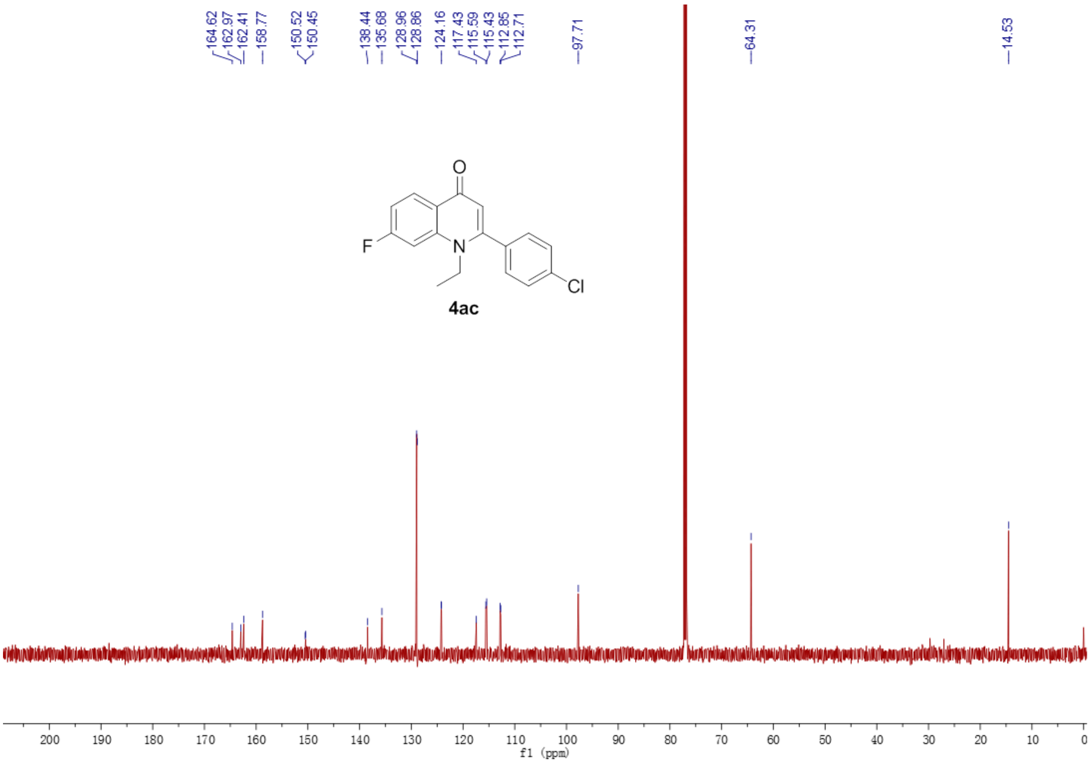
164.62
162.97
162.41
158.77
150.52
150.45
138.44
135.68
128.96
128.86
124.16
117.43
115.59
115.43
112.85
112.71
97.71
64.31
14.53
O
F
N
Cl
f1 (ppm)

4ac

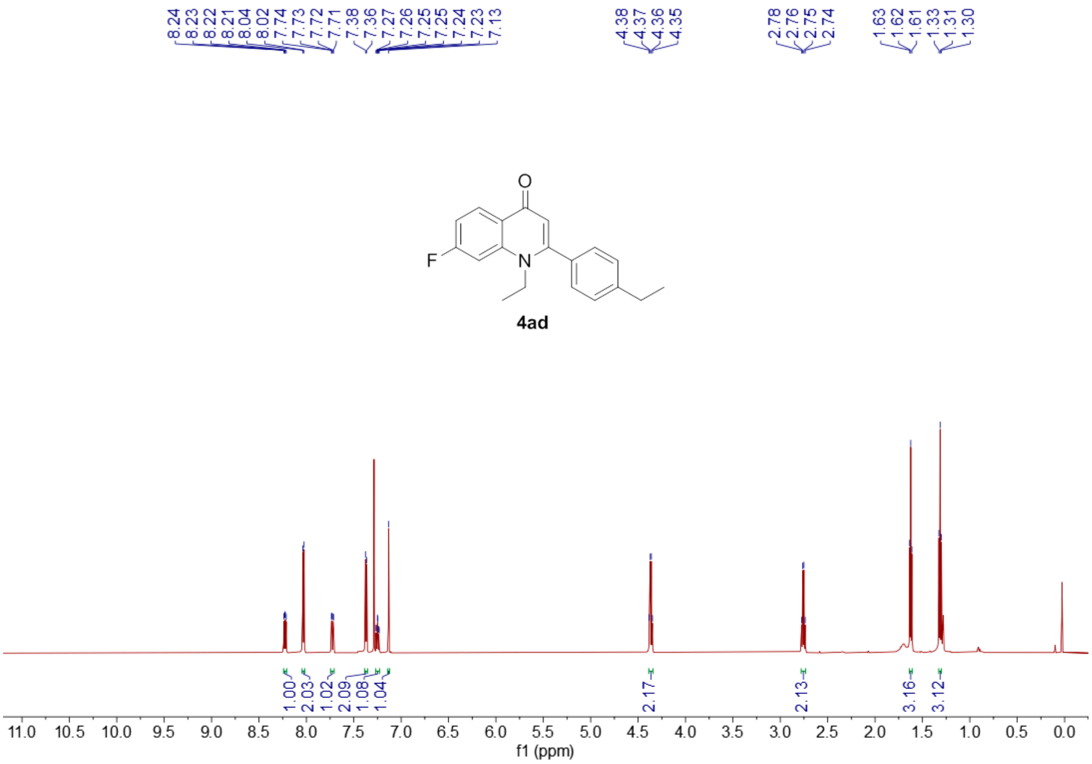
8.24
8.23
8.22
8.21
8.04
8.02
7.74
7.73
7.72
7.71
7.38
7.36
7.27
7.26
7.25
7.25
7.24
7.23
7.13
4.38
4.37
4.36
4.35
2.78
2.76
2.75
2.74
1.63
1.62
1.61
1.33
1.31
1.30
O
F
N
4ad
1.00
2.03
1.02
2.09
1.08
1.04
2.17
2.13
3.16
3.12
11.0 10.5 10.0 9.5 9.0 8.5 8.0 7.5 7.0 6.5 6.0 5.5 5.0 4.5 4.0 3.5 3.0 2.5 2.0 1.5 1.0 0.5 0.0
f1 (ppm)

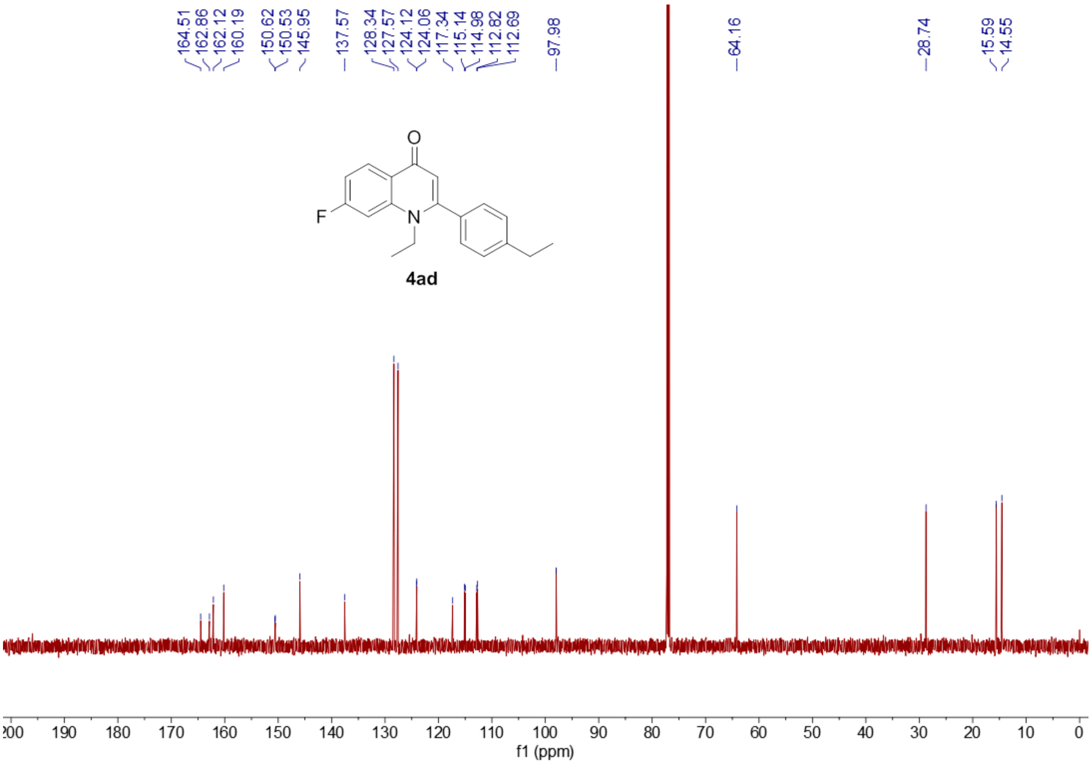
164.51
162.86
162.12
160.19
150.62
150.53
145.95
137.57
128.34
127.57
124.12
124.06
117.34
115.14
114.98
112.82
112.69
97.98
64.16
28.74
15.59
14.55
O
F
N
4ad
200 190 180 170 160 150 140 130 120 110 100 90 80 70 60 50 40 30 20 10 0
f1 (ppm)

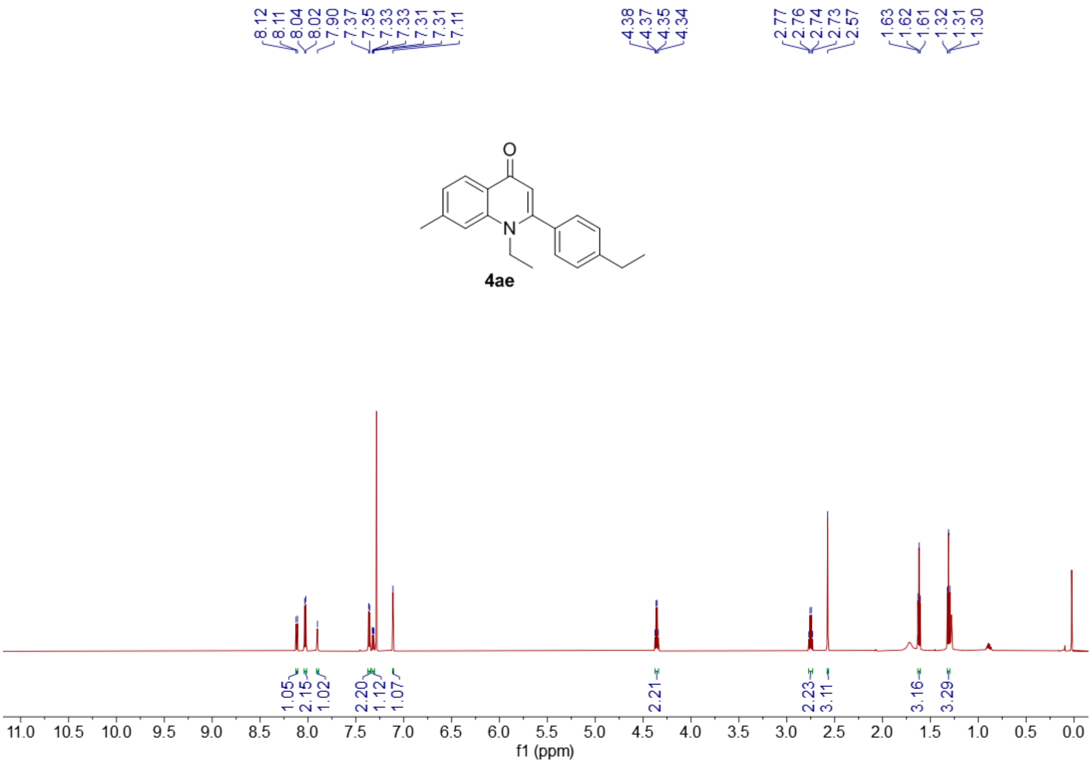

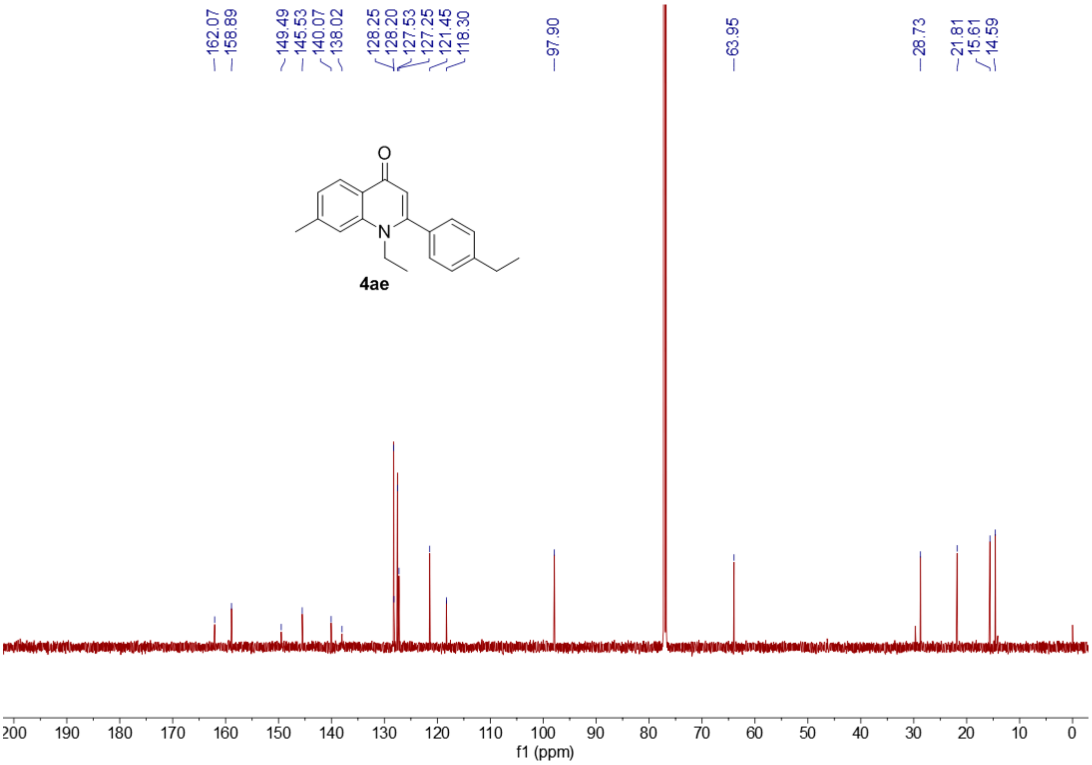

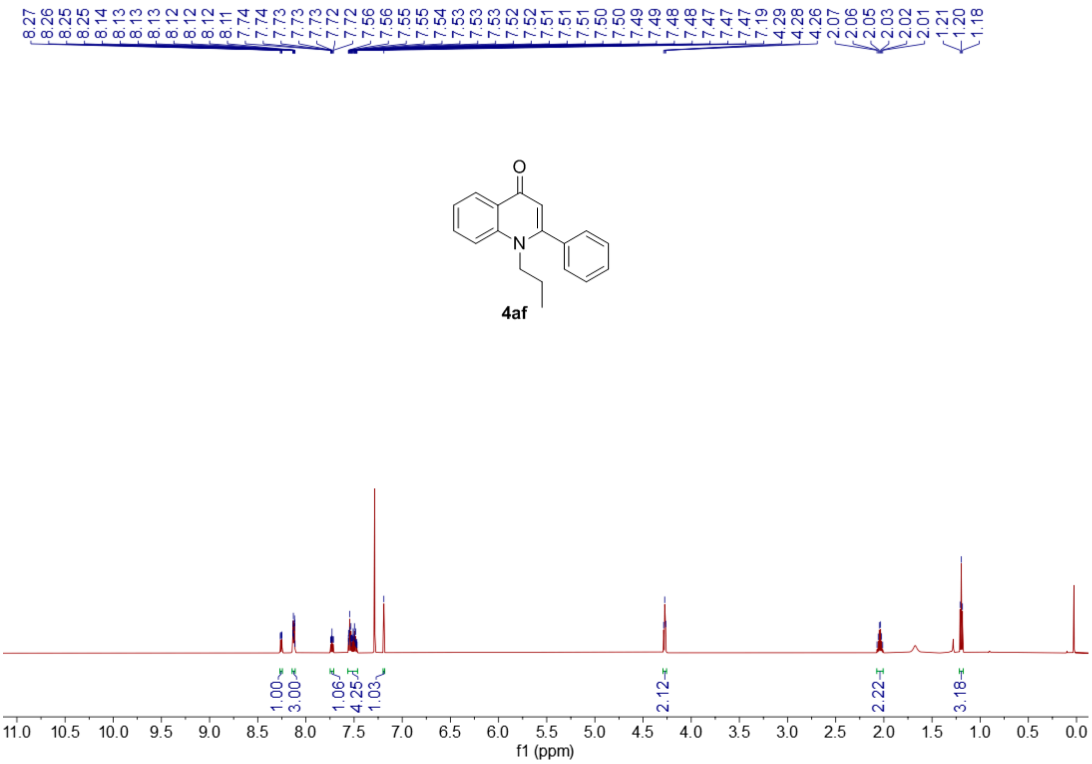

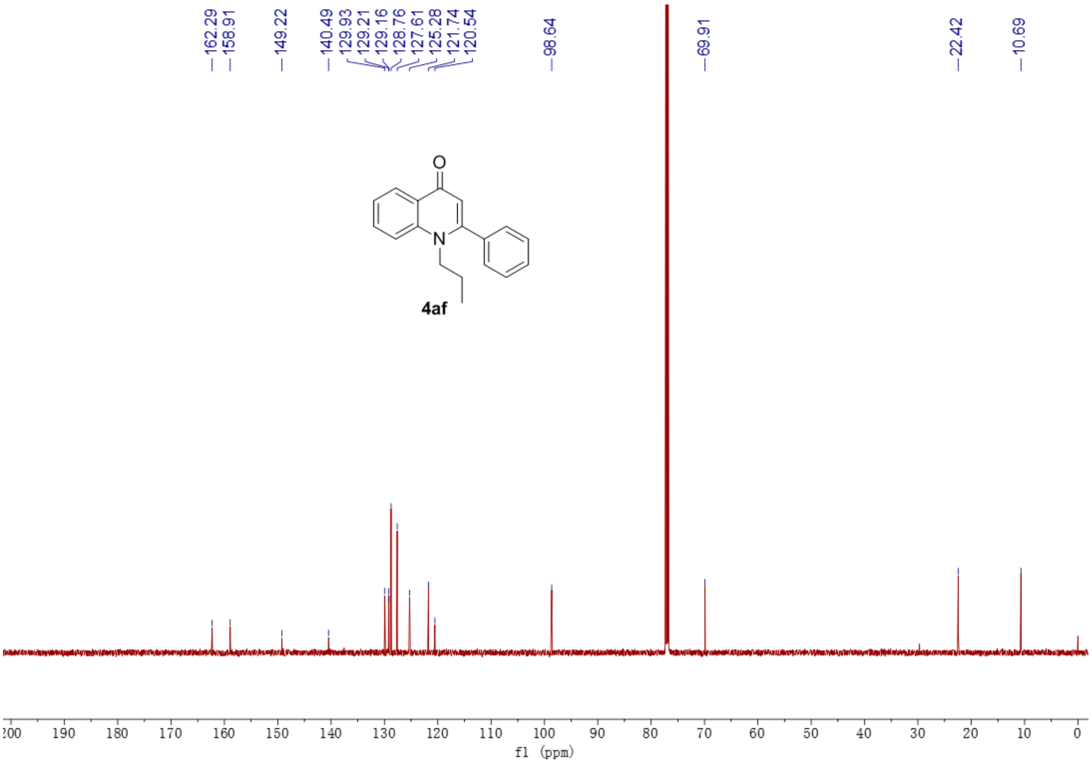

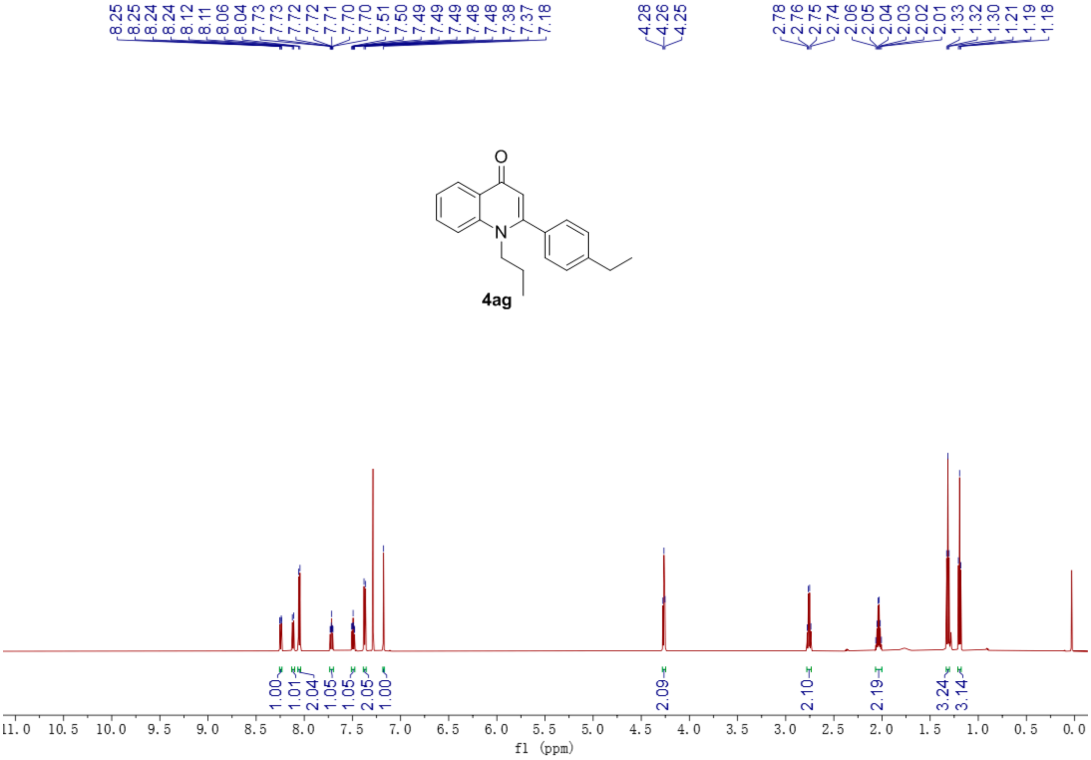
8.25
8.25
8.24
8.12
8.11
8.06
8.04
7.73
7.73
7.72
7.71
7.70
7.70
7.51
7.50
7.49
7.49
7.49
7.48
7.48
7.38
7.37
7.18
4.28
4.26
4.25
2.78
2.76
2.75
2.74
2.06
2.05
2.04
2.03
2.02
2.01
1.33
1.32
1.30
1.21
1.19
1.18
O
N
4ag
1.00
1.01
2.04
1.05
1.05
2.05
1.00
2.09
2.10
2.19
3.24
3.14
f1 (ppm)

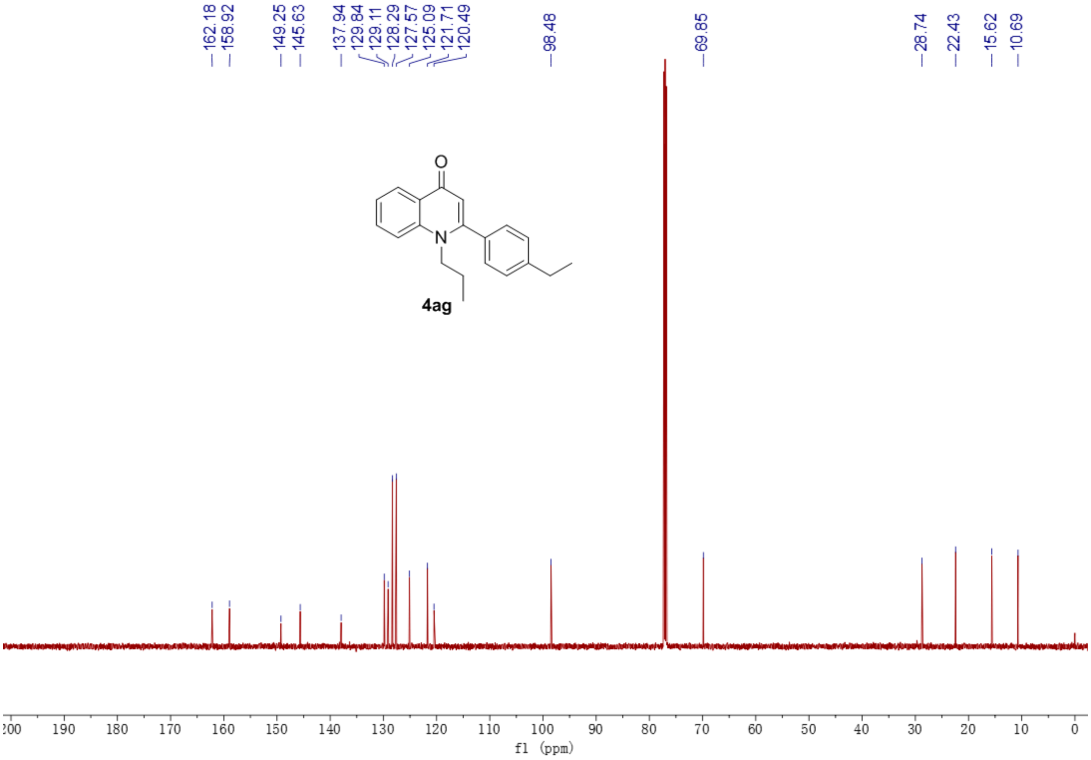
162.18
158.92
149.25
145.63
137.94
129.84
129.11
128.29
127.57
125.09
121.71
120.49
98.48
69.85
28.74
22.43
15.62
10.69
O
N
4ag
f1 (ppm)

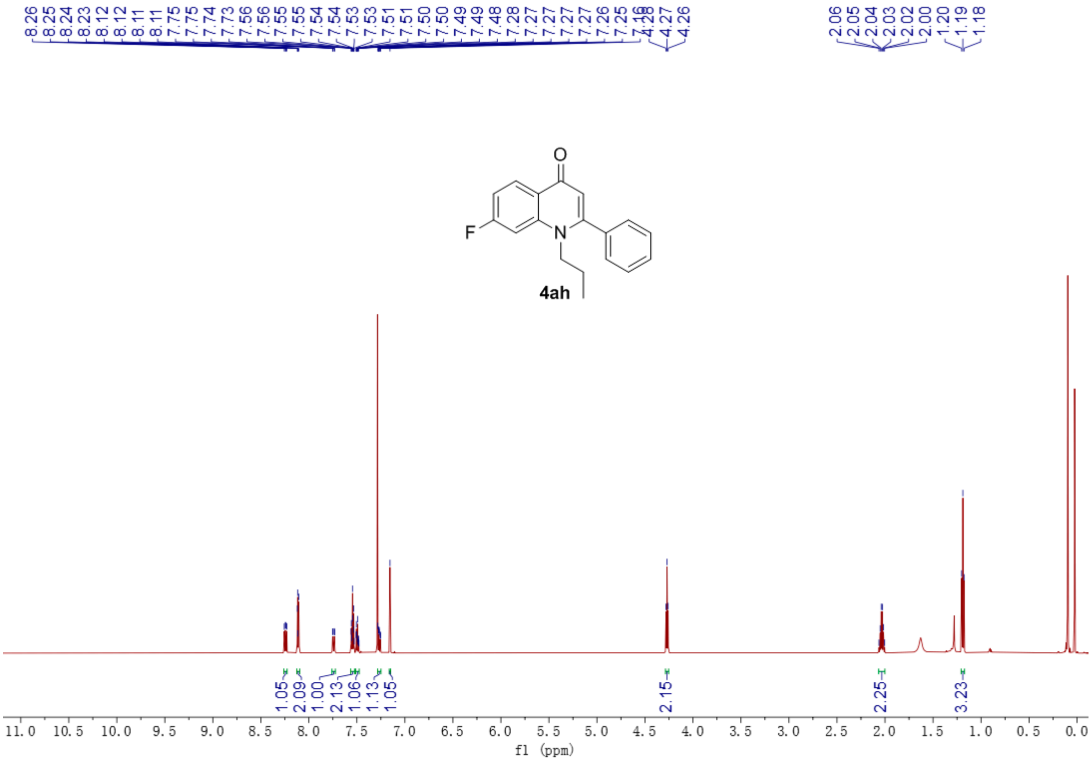
8.26 8.25 8.24 8.23 8.12 8.12 8.11 8.11 7.75 7.75 7.74 7.73 7.56 7.56 7.55 7.55 7.54 7.54 7.53 7.53 7.51 7.51 7.50 7.49 7.49 7.48 7.28 7.27 7.27 7.27 7.27 7.26 7.25 7.16 4.28 4.27 4.26
2.06 2.05 2.04 2.03 2.02 2.00 1.20 1.19 1.18
F
N
O
4ah
1.05 2.09 1.00 2.13 1.06 1.13 1.05 2.15 2.25 3.23
11.0 10.5 10.0 9.5 9.0 8.5 8.0 7.5 7.0 6.5 6.0 5.5 5.0 4.5 4.0 3.5 3.0 2.5 2.0 1.5 1.0 0.5 0.0
f1 (ppm)

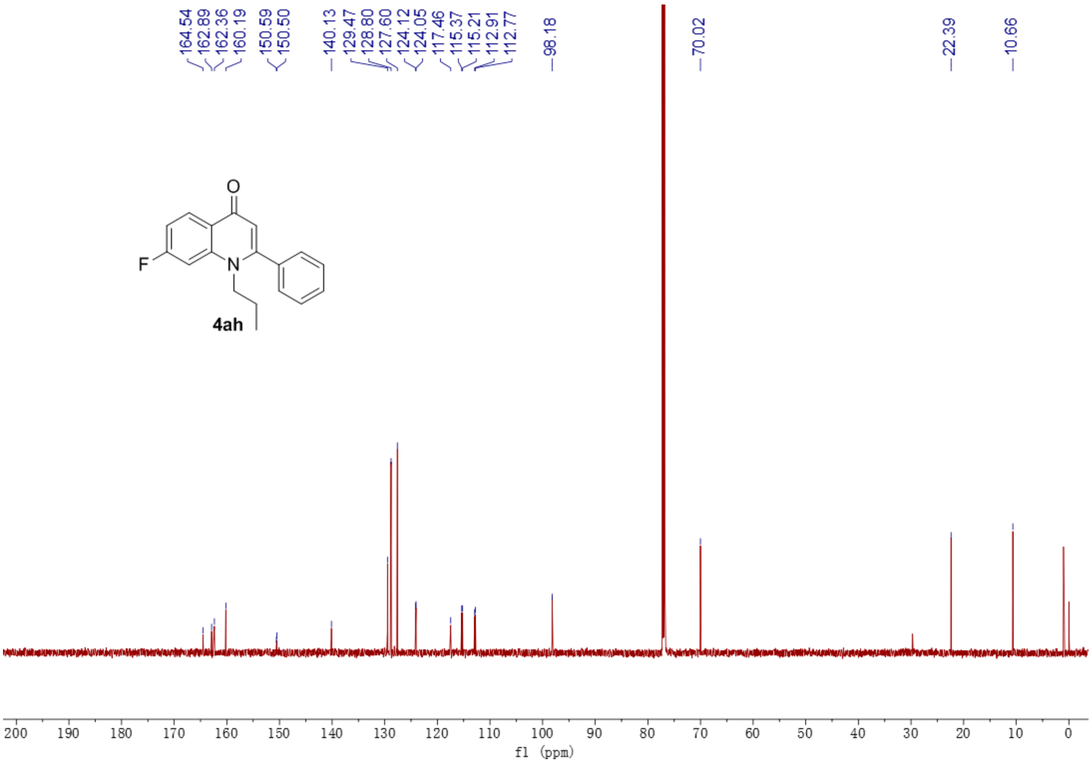
164.54 162.89 162.36 160.19 150.59 150.50 140.13 129.47 128.80 127.60 124.12 124.05 117.46 115.37 115.21 112.91 112.77 98.18 70.02 22.39 10.66
F
N
O
4ah
200 190 180 170 160 150 140 130 120 110 100 90 80 70 60 50 40 30 20 10 0
f1 (ppm)

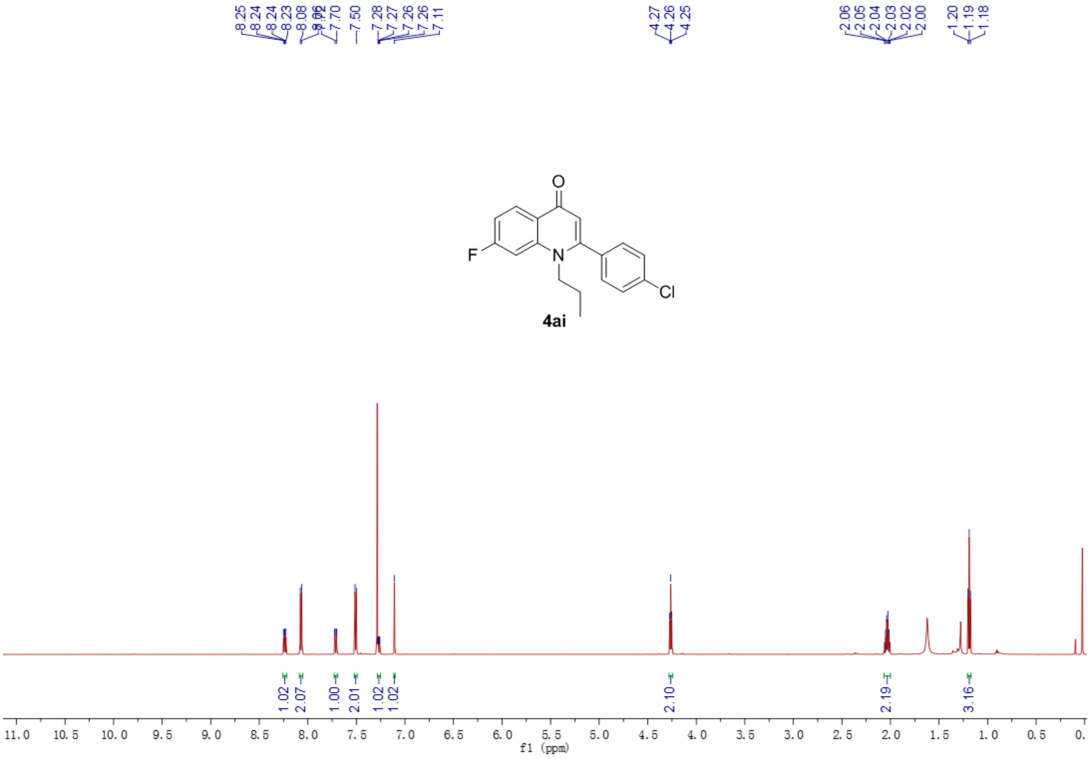
8.25
8.24
8.24
8.23
8.08
7.70
7.50
7.28
7.27
7.26
7.26
7.11
4.27
4.26
4.25
2.06
2.05
2.04
2.03
2.02
2.00
1.20
1.19
1.18
O
F
N
Cl
4ai
1.02
2.07
1.00
2.01
1.02
1.02
2.10
2.19
3.16
f1 (ppm)

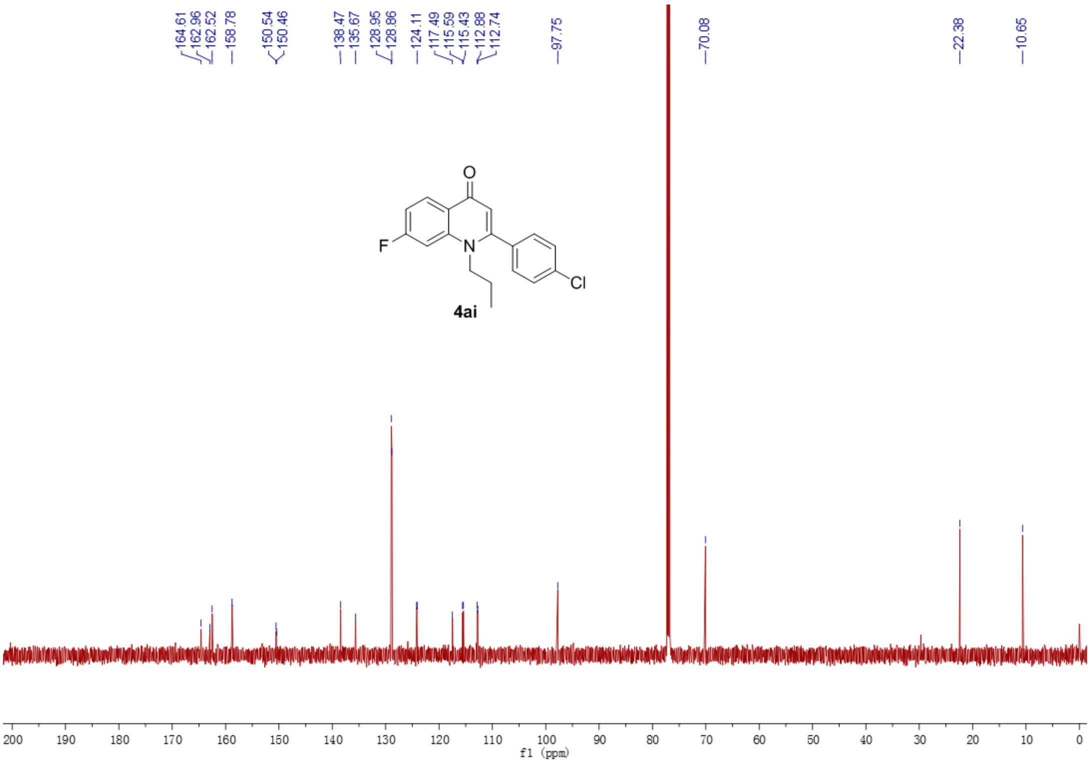
164.61
162.96
162.52
158.78
150.54
150.46
138.47
135.67
128.95
128.86
124.11
117.49
115.59
115.43
112.88
112.74
97.75
70.08
22.38
10.65
O
F
N
Cl
4ai
f1 (ppm)

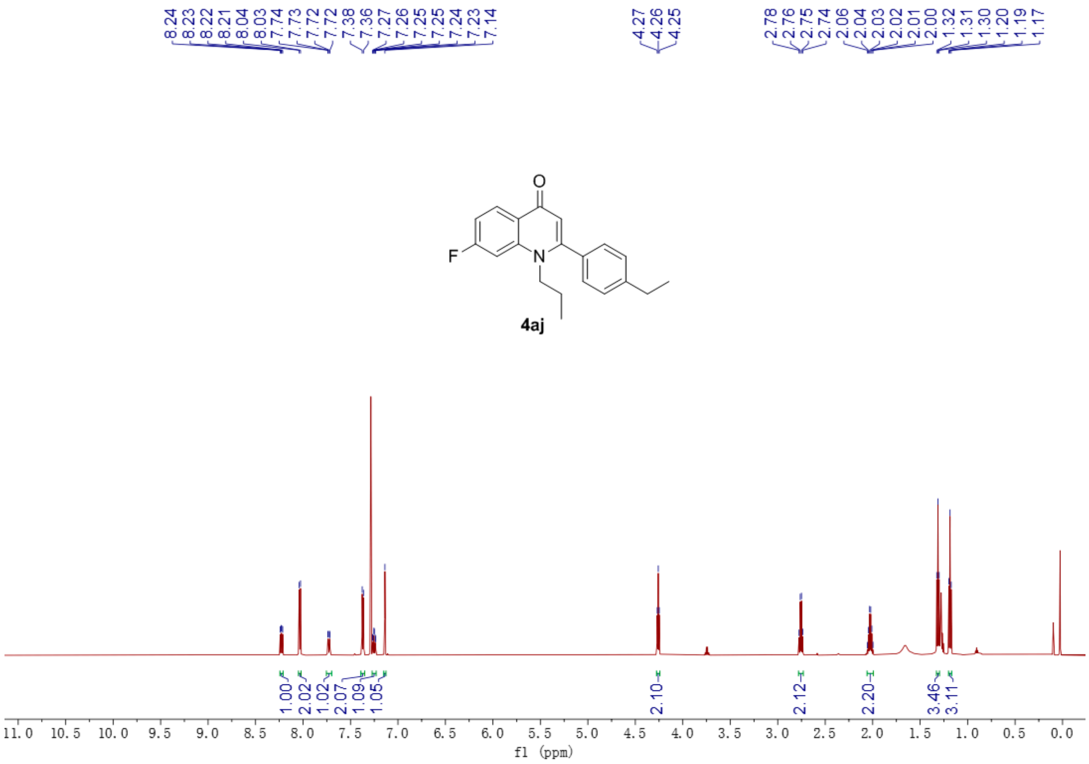
8.24
8.23
8.22
8.21
8.04
8.03
7.74
7.73
7.72
7.72
7.38
7.36
7.27
7.26
7.25
7.25
7.24
7.23
7.14
4.27
4.26
4.25
2.78
2.76
2.75
2.74
2.06
2.04
2.03
2.02
2.01
2.00
1.32
1.31
1.30
1.20
1.19
1.17
O
F
N
4aj
1.00
2.02
1.02
2.07
1.09
1.05
2.10
2.12
2.20
3.46
3.11
11.0 10.5 10.0 9.5 9.0 8.5 8.0 7.5 7.0 6.5 6.0 5.5 5.0 4.5 4.0 3.5 3.0 2.5 2.0 1.5 1.0 0.5 0.0
f1 (ppm)

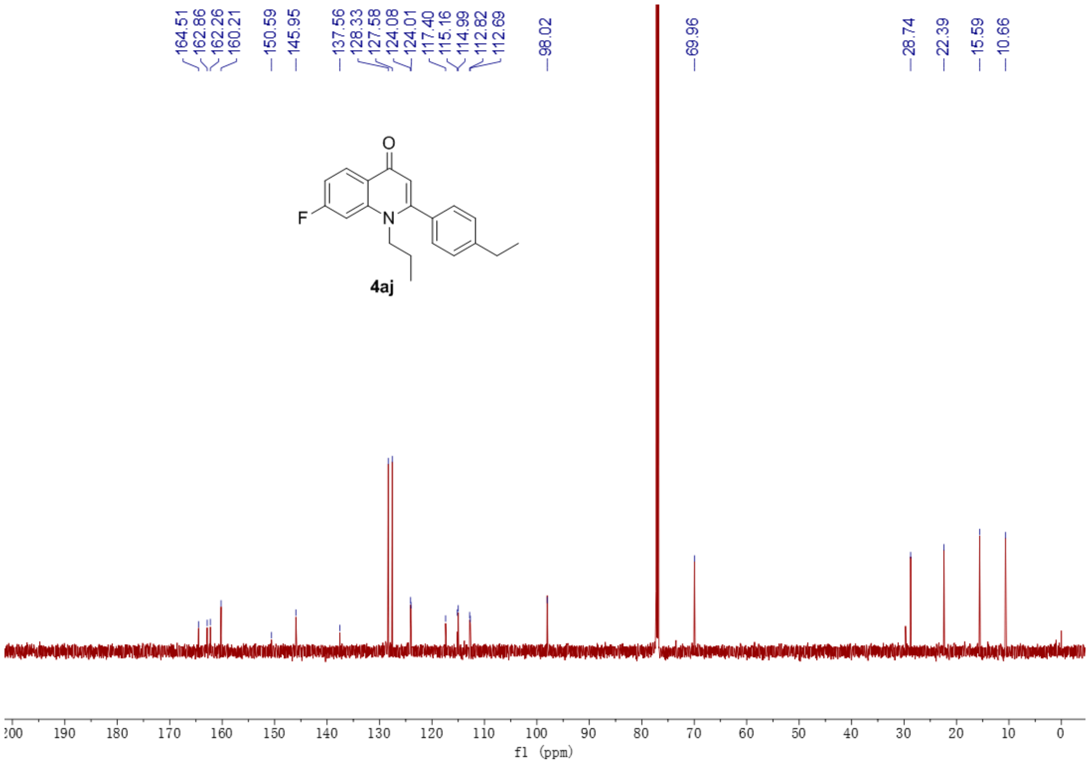
164.51
162.86
162.26
160.21
150.59
145.95
137.56
128.33
127.58
124.08
124.01
117.40
115.16
114.99
112.82
112.69
98.02
69.96
28.74
22.39
15.59
10.66
O
F
N
4aj
200 190 180 170 160 150 140 130 120 110 100 90 80 70 60 50 40 30 20 10 0
f1 (ppm)

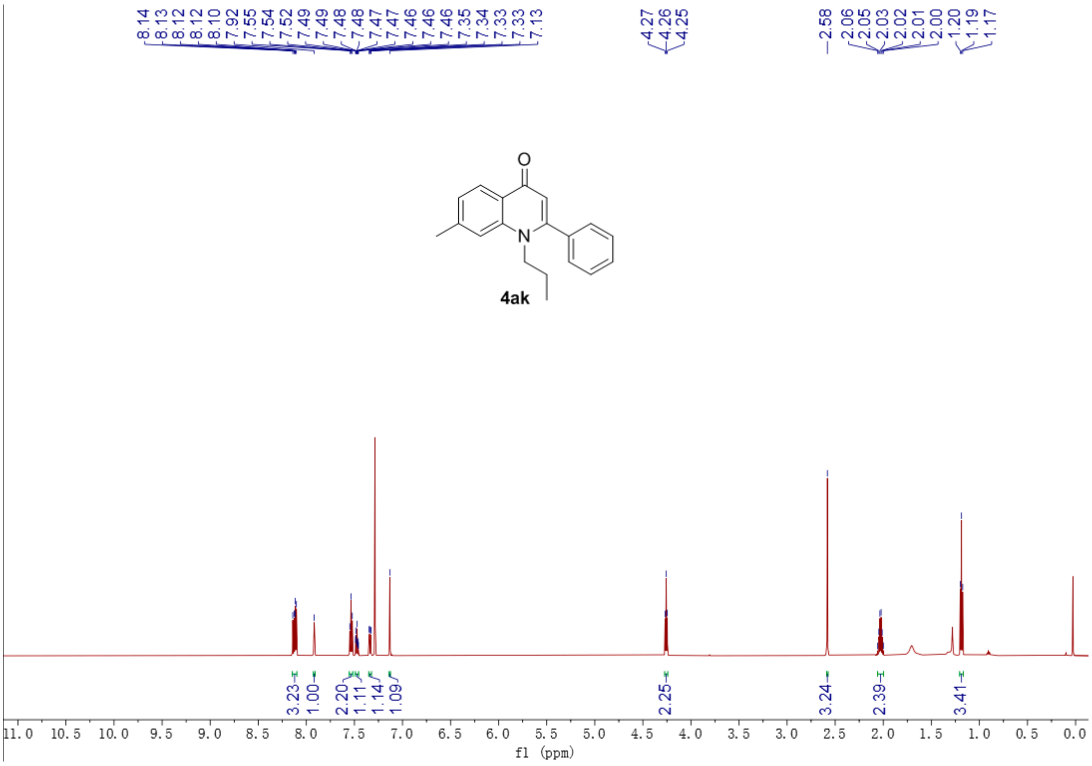
8.14
8.13
8.12
8.12
8.10
7.92
7.55
7.54
7.52
7.49
7.49
7.48
7.48
7.47
7.47
7.46
7.46
7.46
7.35
7.34
7.33
7.33
7.13
4.27
4.26
4.25
2.58
2.06
2.05
2.03
2.02
2.01
2.00
1.20
1.19
1.17
O
N
4ak
3.23
1.00
2.20
1.11
1.14
1.09
2.25
3.24
2.39
3.41
f1 (ppm)

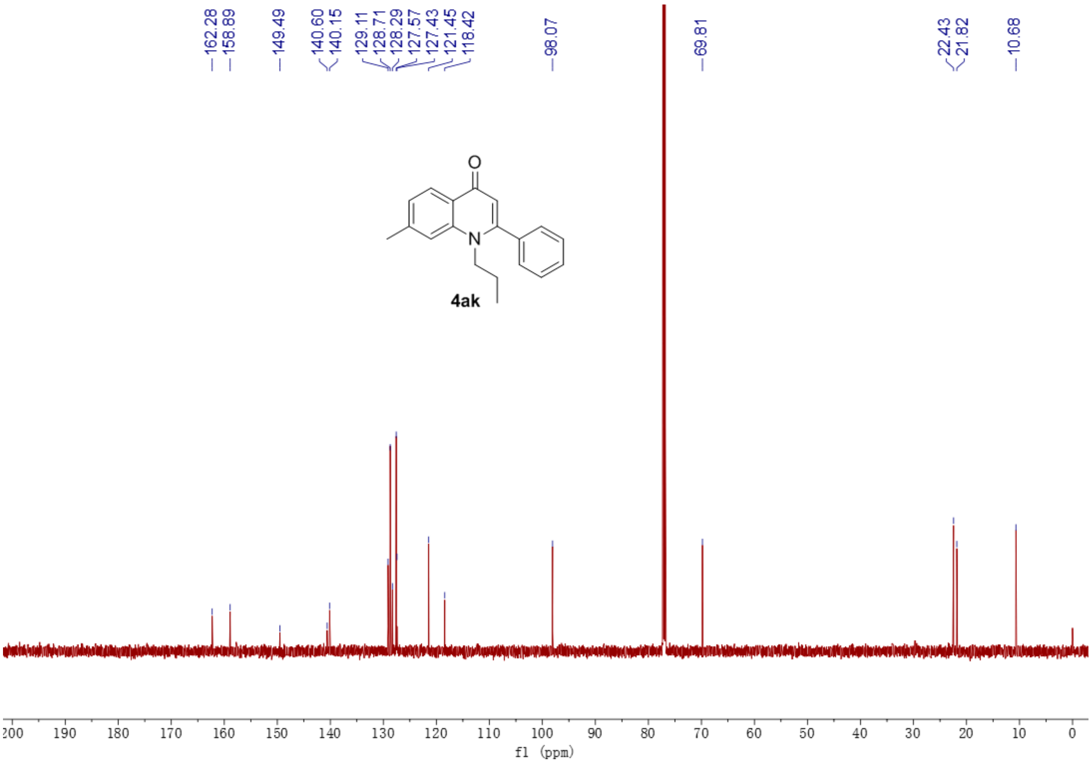
162.28
158.89
149.49
140.60
140.15
129.11
128.71
128.29
127.57
127.43
121.45
118.42
98.07
69.81
22.43
21.82
10.68
O
N
4ak
f1 (ppm)

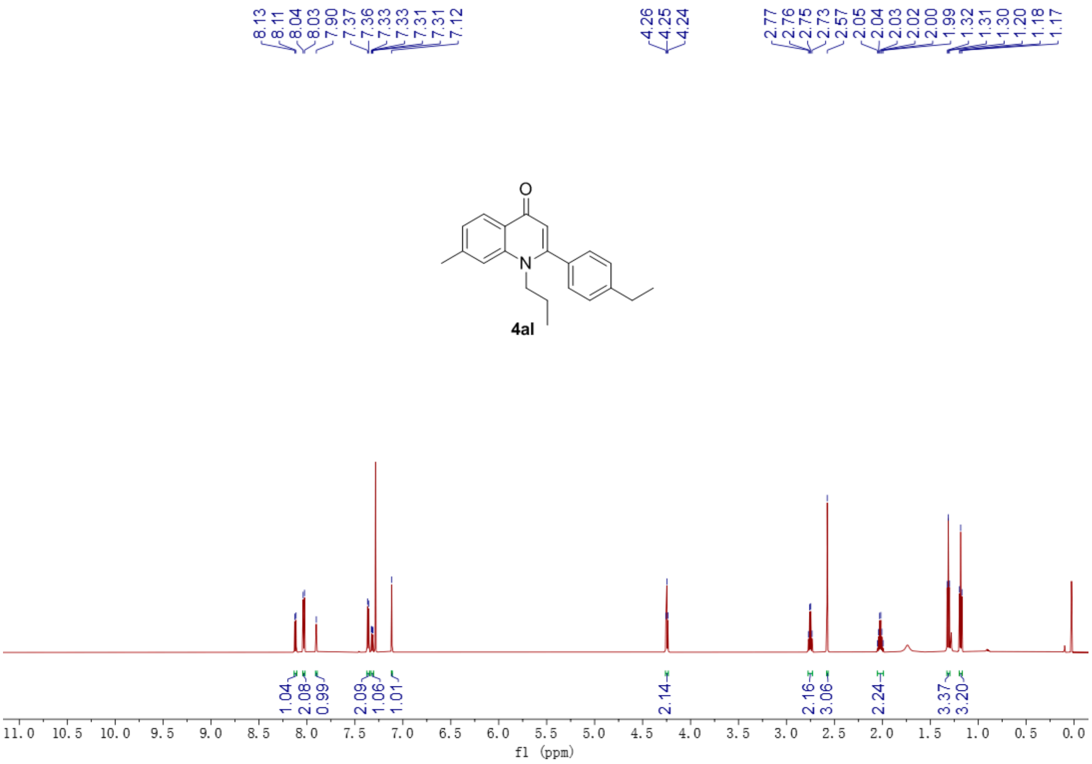
8.13
8.11
8.04
8.03
7.90
7.37
7.36
7.33
7.33
7.31
7.31
7.12
4.26
4.25
4.24
2.77
2.76
2.75
2.73
2.57
2.05
2.04
2.03
2.02
2.00
1.99
1.32
1.31
1.30
1.20
1.18
1.17
4al
1.04
2.08
0.99
2.09
1.06
1.01
2.14
2.16
3.06
2.24
3.37
3.20
f1 (ppm)

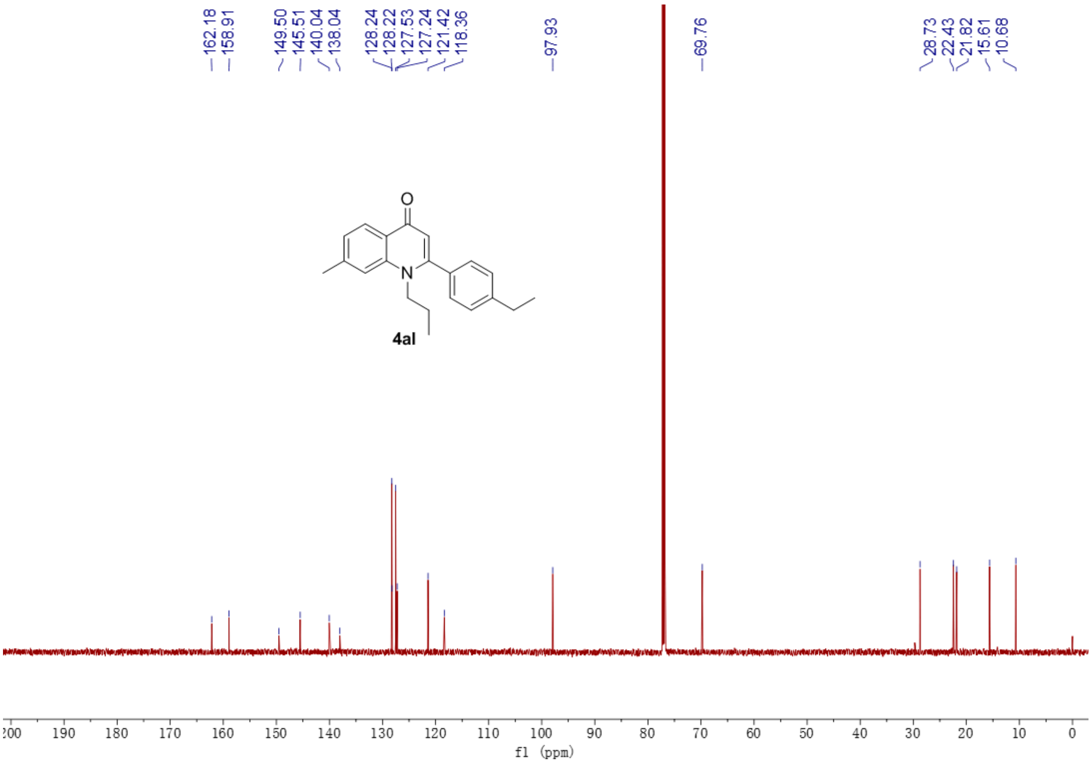
162.18
158.91
149.50
145.51
140.04
138.04
128.24
128.22
127.53
127.24
121.42
118.36
97.93
69.76
28.73
22.43
21.82
15.61
10.68
4al
f1 (ppm)

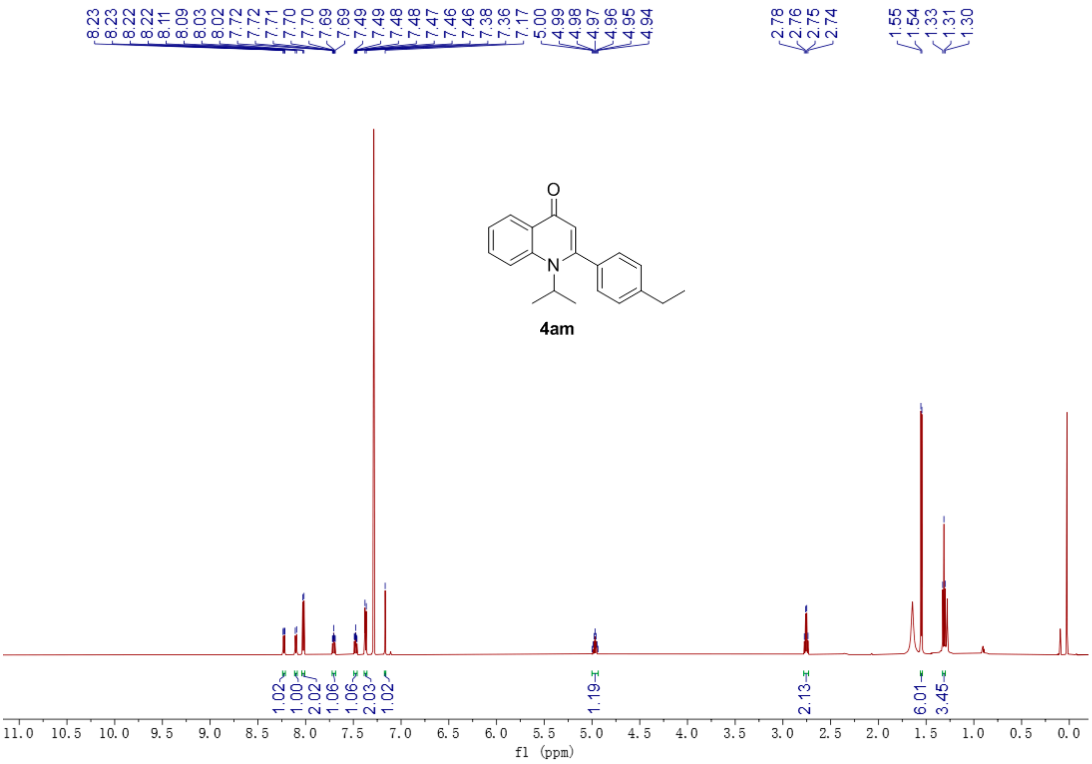
8.23
8.23
8.22
8.22
8.11
8.09
8.03
8.02
7.72
7.72
7.71
7.70
7.70
7.69
7.69
7.49
7.49
7.48
7.48
7.47
7.46
7.46
7.38
7.36
7.17
5.00
4.99
4.98
4.97
4.96
4.95
4.94
2.78
2.76
2.75
2.74
1.55
1.54
1.33
1.31
1.30
O
N
4am
1.02
1.00
2.02
1.06
1.06
2.03
1.02
1.19
2.13
6.01
3.45
f1 (ppm)

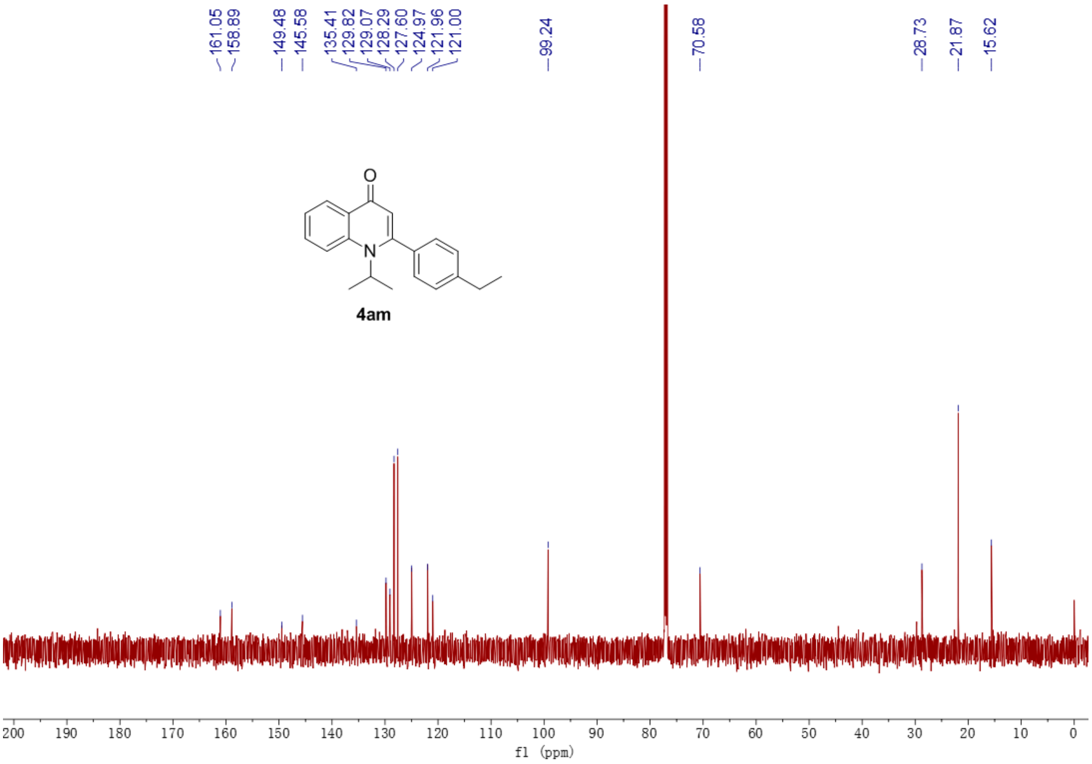
161.05
158.89
149.48
145.58
135.41
129.82
129.07
128.29
127.60
124.97
121.96
121.00
99.24
70.58
28.73
21.87
15.62
O
N
4am
f1 (ppm)

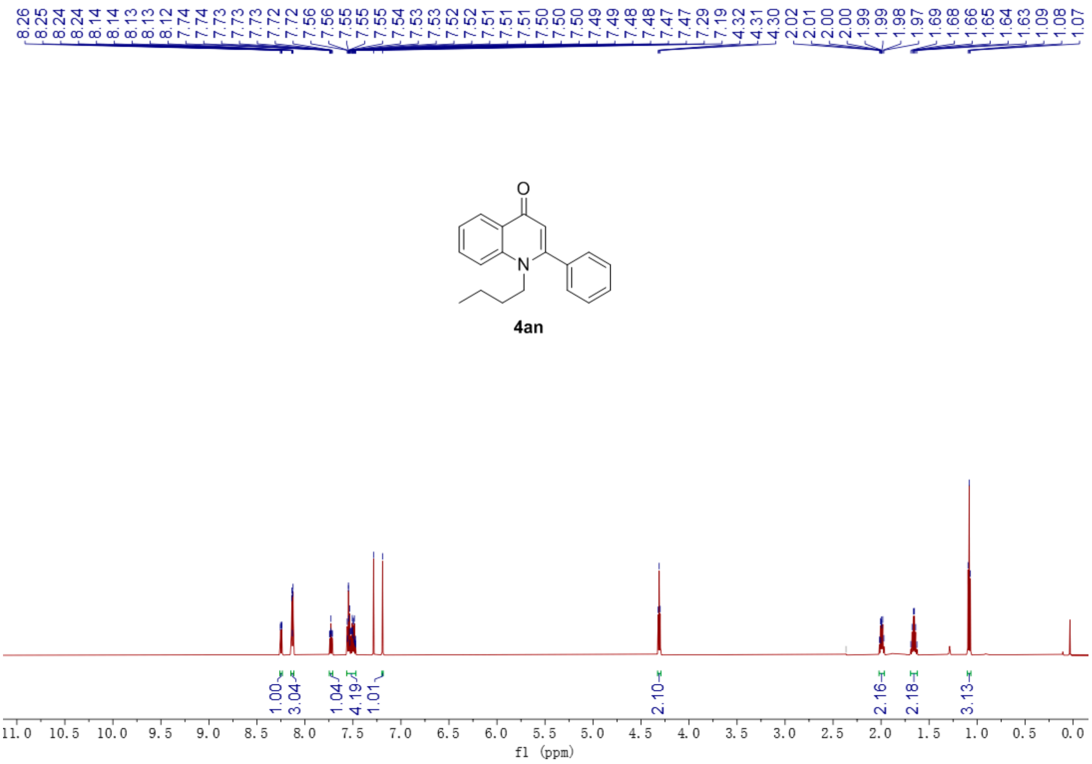

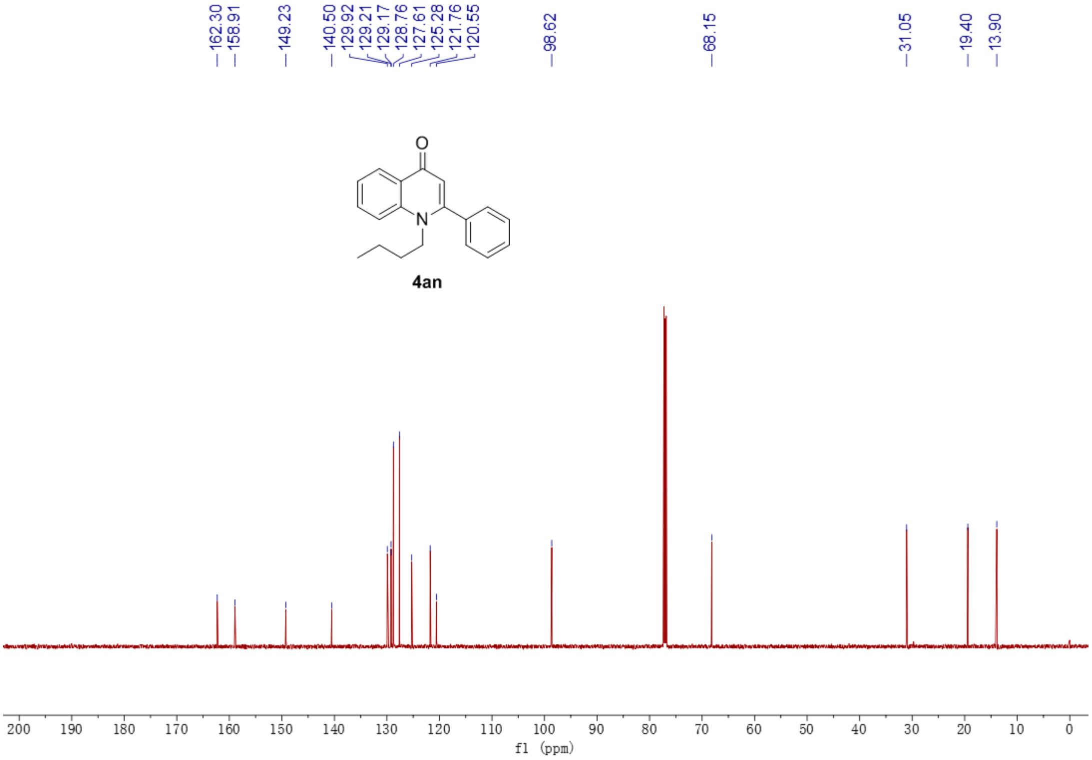

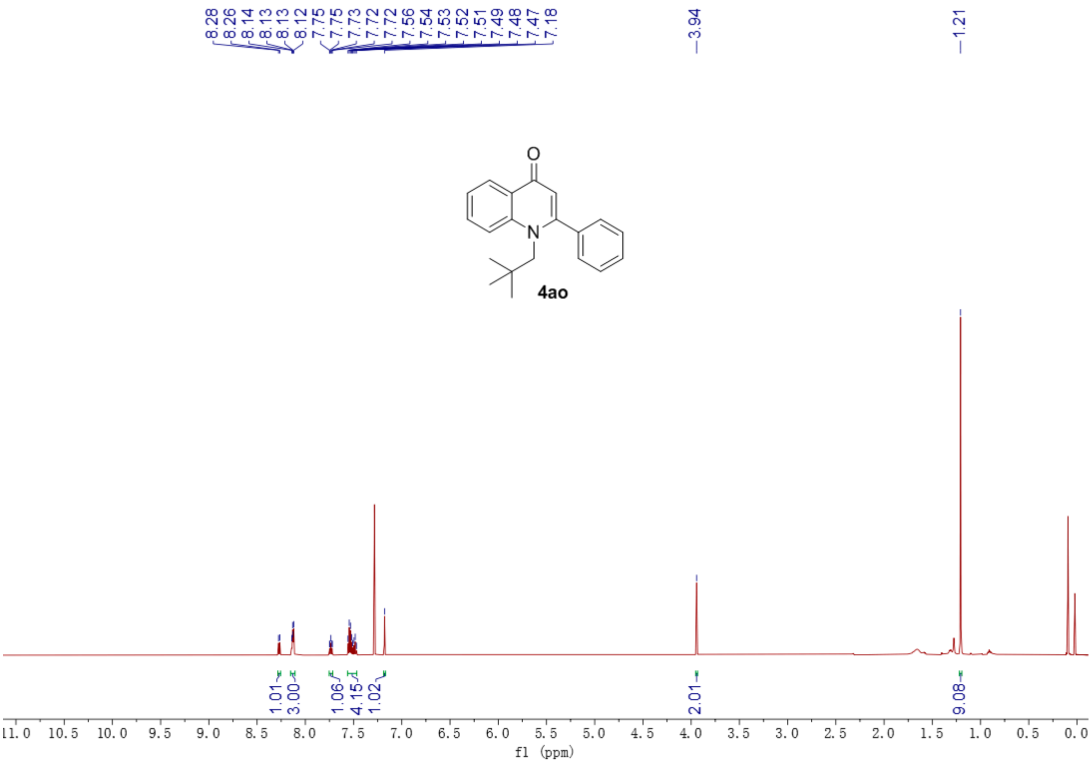
8.28
8.26
8.14
8.13
8.13
8.12
7.75
7.75
7.73
7.72
7.72
7.56
7.54
7.53
7.52
7.51
7.49
7.48
7.47
7.18
3.94
1.21
4ao
1.01
3.00
1.06
4.15
1.02
2.01
9.08
f1 (ppm)

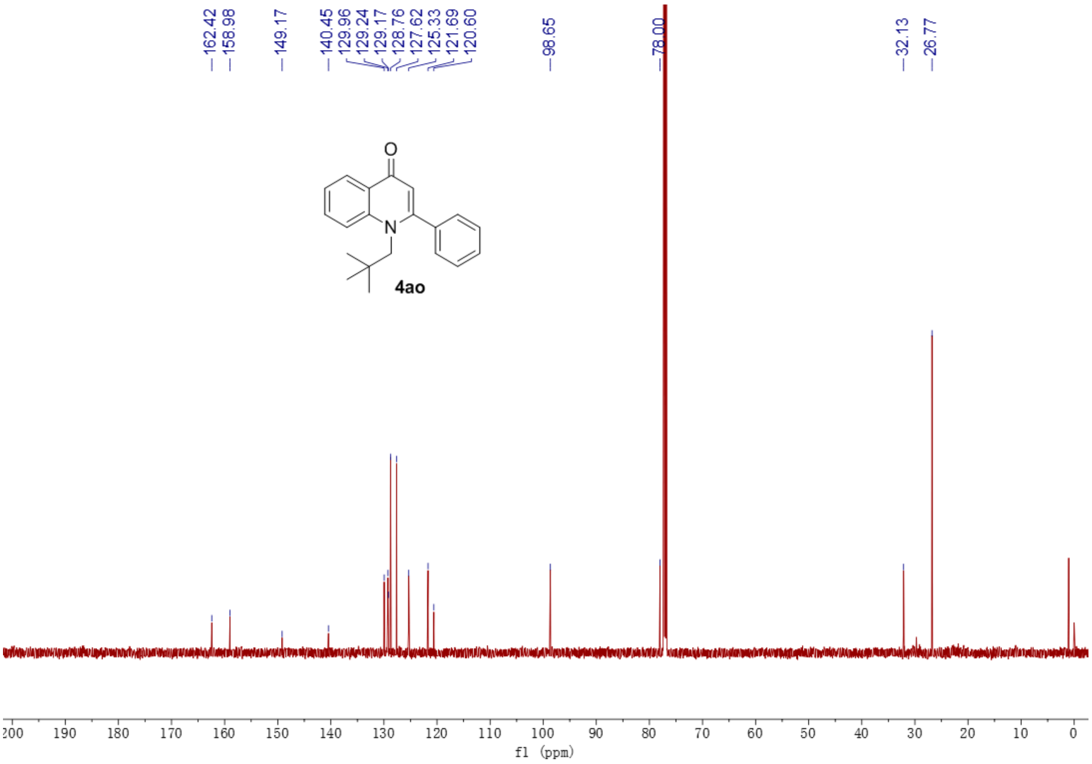
162.42
158.98
149.17
140.45
129.96
129.24
129.17
128.76
127.62
125.33
121.69
120.60
98.65
78.00
32.13
26.77
4ao
f1 (ppm)

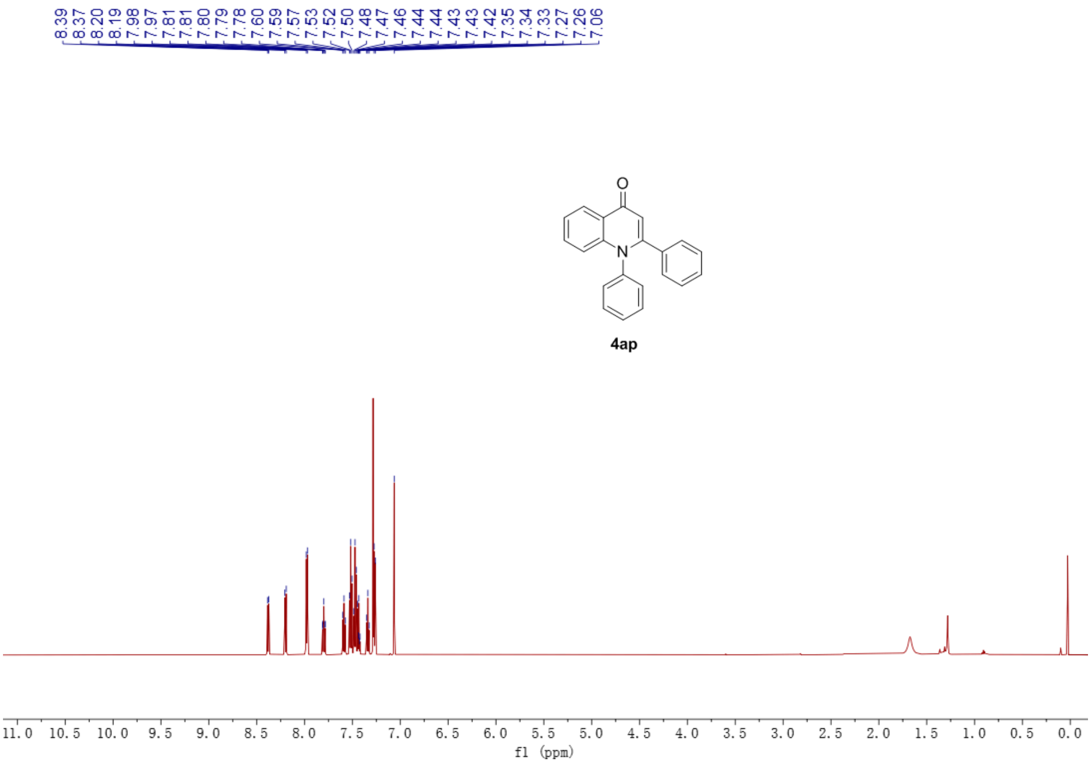
8.39 8.37 8.20 8.19 7.98 7.97 7.81 7.81 7.80 7.79 7.78 7.60 7.59 7.57 7.53 7.52 7.50 7.48 7.47 7.46 7.44 7.44 7.43 7.43 7.42 7.35 7.34 7.33 7.27 7.26 7.06
O
N
4ap
11.0 10.5 10.0 9.5 9.0 8.5 8.0 7.5 7.0 6.5 6.0 5.5 5.0 4.5 4.0 3.5 3.0 2.5 2.0 1.5 1.0 0.5 0.0
f1 (ppm)

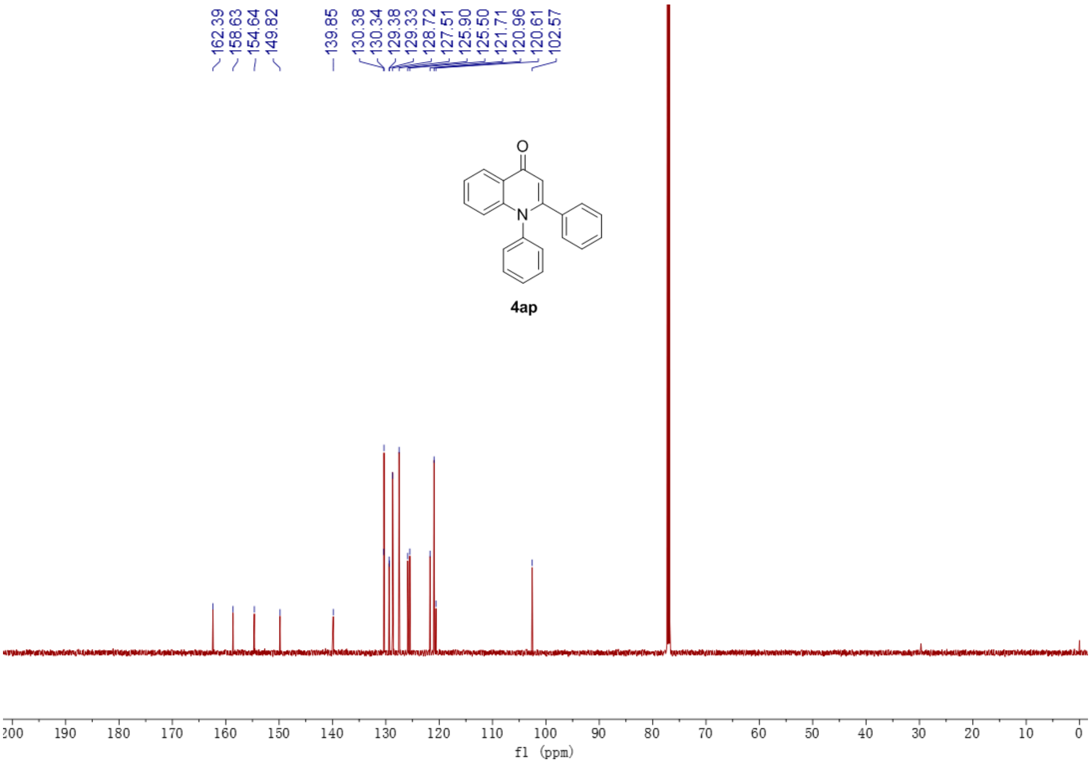
162.39 158.63 154.64 149.82
139.85
130.38 130.34 129.38 129.33 128.72 127.51 125.90 125.50 121.71 120.96 120.61 102.57
O
N
4ap
200 190 180 170 160 150 140 130 120 110 100 90 80 70 60 50 40 30 20 10 0
f1 (ppm)

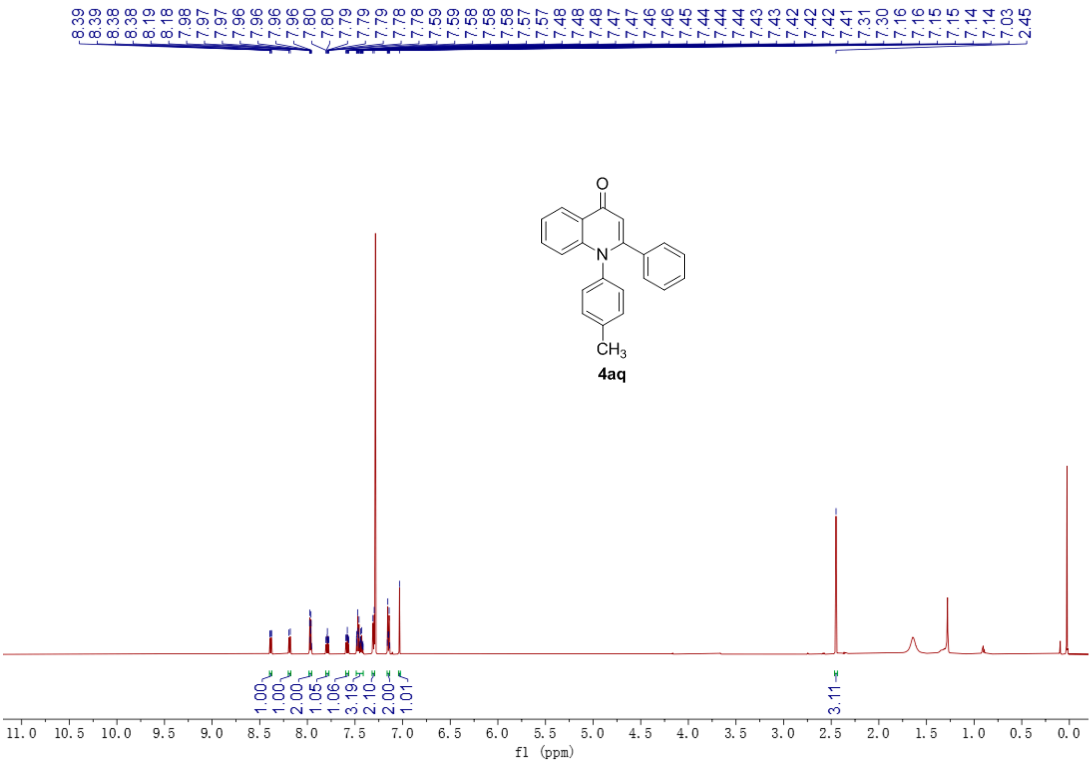
8.39
8.39
8.38
8.38
8.19
8.18
7.98
7.97
7.97
7.96
7.96
7.96
7.80
7.80
7.79
7.79
7.79
7.78
7.78
7.59
7.59
7.58
7.58
7.58
7.57
7.57
7.48
7.48
7.48
7.47
7.47
7.46
7.46
7.45
7.44
7.44
7.44
7.43
7.43
7.42
7.42
7.42
7.41
7.31
7.30
7.16
7.16
7.15
7.14
7.14
7.03
2.45
4aq
1.00
1.00
2.00
1.05
1.06
3.19
2.10
2.00
1.01
3.11
f1 (ppm)

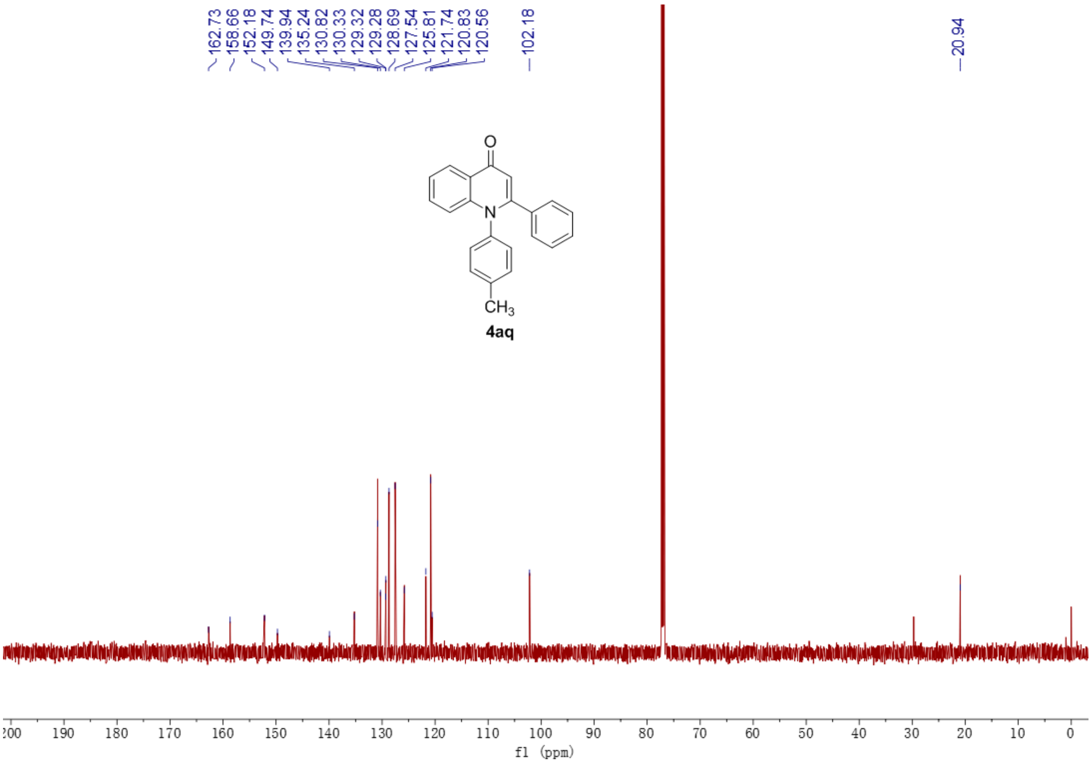
162.73
158.66
152.18
149.74
139.94
135.24
130.82
130.33
129.32
129.28
128.69
127.54
125.81
121.74
120.83
120.56
102.18
20.94
4aq
f1 (ppm)

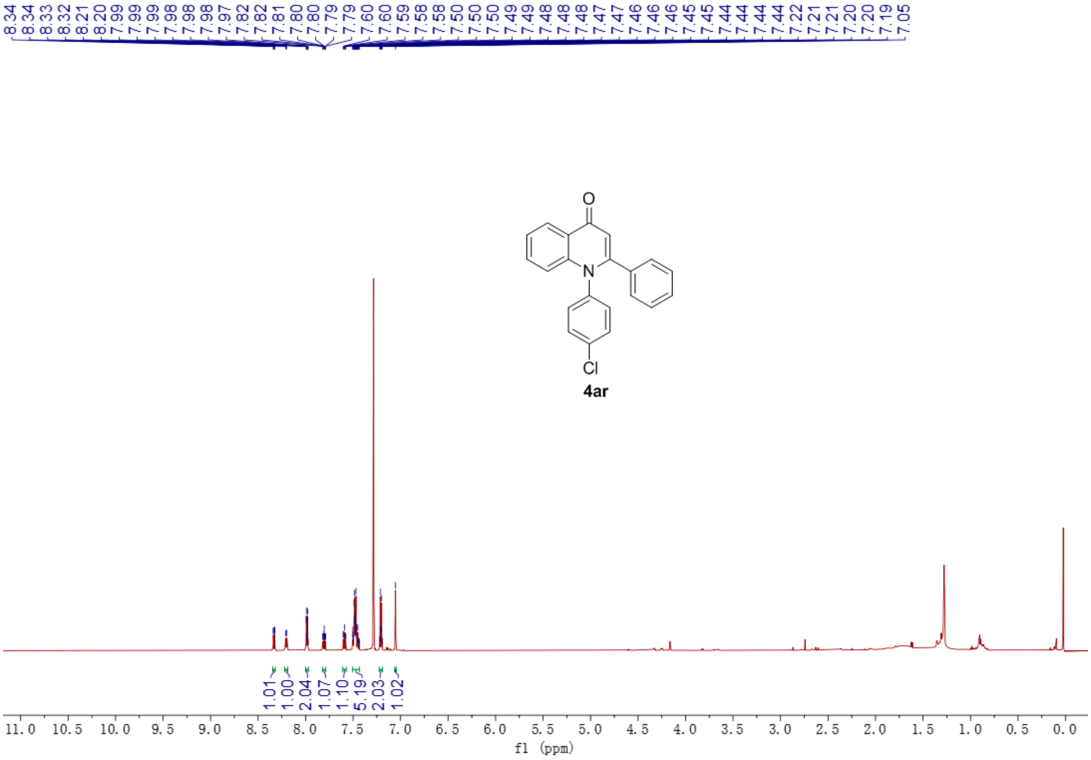
8.34
8.34
8.33
8.32
8.21
8.20
7.99
7.99
7.99
7.98
7.98
7.98
7.97
7.82
7.82
7.81
7.80
7.80
7.79
7.79
7.60
7.60
7.59
7.58
7.58
7.50
7.50
7.50
7.49
7.49
7.48
7.48
7.48
7.47
7.47
7.46
7.46
7.46
7.45
7.45
7.44
7.44
7.44
7.44
7.22
7.21
7.21
7.20
7.20
7.19
7.05
O
N
Cl
4ar
1.01
1.00
2.04
1.07
1.10
5.19
2.03
1.02
11.0 10.5 10.0 9.5 9.0 8.5 8.0 7.5 7.0 6.5 6.0 5.5 5.0 4.5 4.0 3.5 3.0 2.5 2.0 1.5 1.0 0.5 0.0
f1 (ppm)

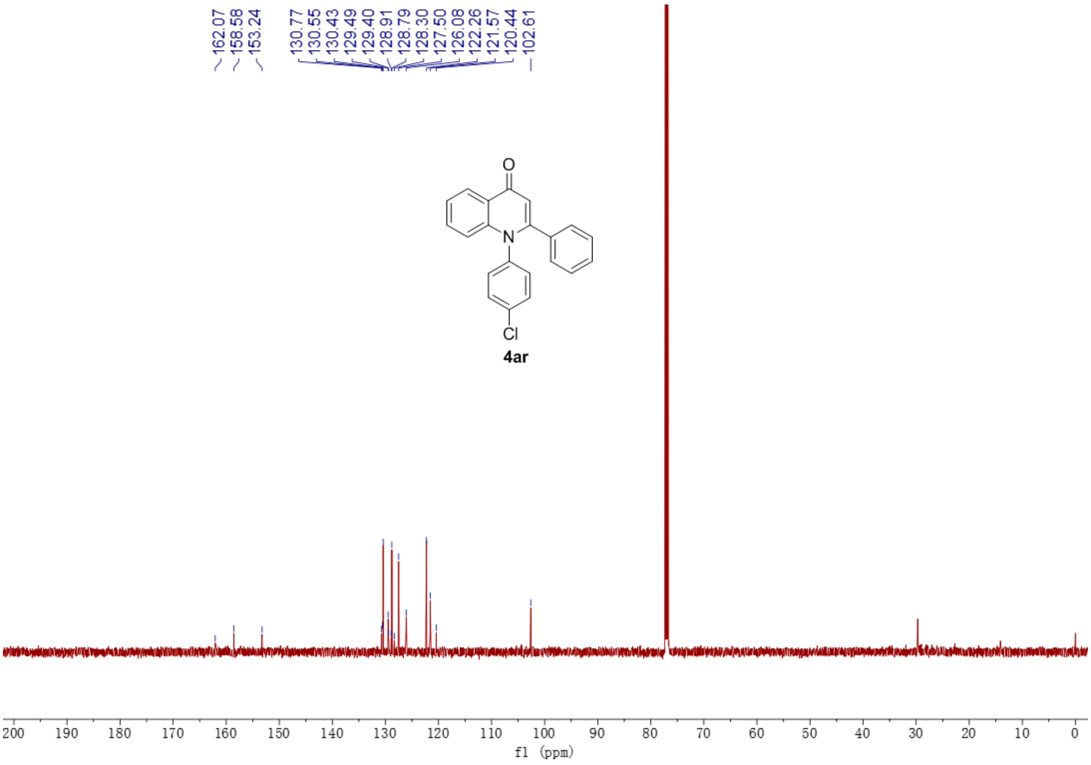
162.07
158.58
153.24
130.77
130.55
130.43
129.49
129.40
128.91
128.79
128.30
127.50
126.08
122.26
121.57
120.44
102.61
O
N
Cl
4ar
200 190 180 170 160 150 140 130 120 110 100 90 80 70 60 50 40 30 20 10 0
f1 (ppm)

## 7. DFT Calculation

*7.1 Computation Details*

The geometries of reactants, products and reaction intermediates were all optimized with M06-2x functional [1] and 6-31G(d,p) basis set [2] using Gaussian program. In order to describe the solvation effect of DMF, the SMD (Solvation Model Based on Density) [3] implicit solvent model was used in all calculations. The vibrational frequency analyses were carried out for these optimized structures with the same calculation method to obtain the zero-point energy and free energy corrections. The thermodynamic correction terms and Gibbs free energy of there structures at 373.15K were then obtained using Shermo program [4]. In order to obtain the electron energy with higher accuracy which has the major impact on the accuracy of Gibbs free energy, single point calculations for the optimized structures with M06-2x functional and 6-311 + G(d,p) basis set were performed. Finally, the single point energy was added to the free energy correction calculated before to obtain the Gibbs free energy. All these DFT calculations above were performed using Gaussian 16 program suite [5].

*7.2 Cartesian Coordination and Energies for the Calculated Species*

**7**

[SMD(DMF)/M062X/6-31G(d,p)] G: -709.1264289 a.u.

| | | | |
|---|---|---|---|
| C(1) | -4.5799 | 1.5482 | -0.0692 |
| C(2) | -4.6544 | 0.1878 | 0.1472 |
| C(3) | -3.4948 | -0.6202 | 0.1473 |
| C(4) | -2.2328 | 0.0093 | -0.0530 |
| C(5) | -2.1998 | 1.3993 | -0.2801 |
| C(6) | -3.3453 | 2.1712 | -0.2964 |
| H(7) | -5.4930 | 2.1357 | -0.0705 |
| H(8) | -5.6157 | -0.2894 | 0.3133 |
| H(9) | -1.2485 | 1.8797 | -0.4777 |
| H(10) | -3.2884 | 3.2371 | -0.4833 |
| C(11) | -0.9839 | -0.7842 | -0.0519 |
| O(12) | -0.9975 | -2.0152 | -0.1298 |
| C(13) | 0.3194 | -0.0752 | 0.0661 |
| C(14) | 1.4614 | -0.7483 | -0.1379 |
| H(15) | 0.3255 | 0.9707 | 0.3469 |
| H(16) | 1.3717 | -1.8007 | -0.4034 |
| C(17) | 2.8279 | -0.2248 | -0.0417 |
| C(18) | 3.8966 | -1.1010 | -0.2750 |
| C(19) | 3.1123 | 1.1131 | 0.2743 |
| C(20) | 5.2136 | -0.6592 | -0.1942 |
| H(21) | 3.6861 | -2.1381 | -0.5208 |
| C(22) | 4.4263 | 1.5544 | 0.3544 |
| H(23) | 2.3037 | 1.8139 | 0.4559 |
| C(24) | 5.4815 | 0.6704 | 0.1210 |
| H(25) | 6.0285 | -1.3516 | -0.3774 |
| H(26) | 4.6315 | 2.5914 | 0.5989 |
| H(27) | 6.5068 | 1.0197 | 0.1843 |

| | | | |
|---|---|---|---|
| N(28) | -3.6195 | -1.9562 | 0.3877 |
| H(29) | -2.8367 | -2.5450 | 0.1457 |
| H(30) | -4.5447 | -2.3540 | 0.3485 |

**11**

[SMD(DMF)/M062X/6-31G(d,p)] G: -1290.407042 a.u.

| | | | |
|---|---|---|---|
| P(1) | -1.9900 | -0.3987 | -0.1171 |
| O(2) | -2.3151 | 0.4993 | 1.1342 |
| O(3) | -3.0760 | -0.5771 | -1.0844 |
| O(4) | -1.3593 | -1.7062 | 0.5378 |
| O(5) | -0.7007 | 0.3259 | -0.8284 |
| P(6) | 0.6140 | 0.7995 | -0.0247 |
| O(7) | 0.3085 | 1.1096 | 1.3929 |
| O(8) | 1.1951 | 1.9905 | -0.8899 |
| O(9) | 1.6048 | -0.3863 | -0.3217 |
| C(10) | 2.9412 | -0.3337 | 0.2576 |
| C(11) | 3.6304 | -1.6329 | -0.0821 |
| H(12) | 2.8406 | -0.1959 | 1.3373 |
| H(13) | 3.4611 | 0.5272 | -0.1708 |
| H(14) | 3.0833 | -2.4777 | 0.3408 |
| H(15) | 3.6990 | -1.7580 | -1.1644 |
| H(16) | 0.8869 | 2.8664 | -0.6119 |
| H(17) | -1.4975 | 0.8174 | 1.5833 |
| H(18) | 4.6401 | -1.6264 | 0.3337 |
| H(19) | -1.1626 | -2.4116 | -0.0971 |

**12**

[SMD(DMF)/M062X/6-31G(d,p)] G: -1999.530921 a.u.

| | | | |
|---|---|---|---|
| C(1) | 2.8972 | -4.4875 | 0.4496 |
| C(2) | 1.5413 | -4.3082 | 0.7069 |
| C(3) | 0.8490 | -3.2441 | 0.1329 |
| C(4) | 1.5424 | -2.3322 | -0.6861 |
| C(5) | 2.9066 | -2.5203 | -0.9276 |
| C(6) | 3.5858 | -3.5982 | -0.3715 |
| H(7) | 3.4157 | -5.3289 | 0.8968 |
| H(8) | 1.0123 | -4.9974 | 1.3579 |
| H(9) | 3.4277 | -1.8196 | -1.5722 |
| H(10) | 4.6409 | -3.7402 | -0.5745 |
| C(11) | 0.8057 | -1.2219 | -1.3527 |
| O(12) | -0.2824 | -1.4738 | -1.8882 |
| C(13) | 1.3421 | 0.1386 | -1.3730 |
| C(14) | 2.2510 | 0.5711 | -0.4780 |
| H(15) | 0.8646 | 0.8090 | -2.0822 |
| H(16) | 2.6243 | -0.1388 | 0.2583 |

| | | | |
|---|---|---|---|
| C(17) | 2.7733 | 1.9325 | -0.3571 |
| C(18) | 3.5840 | 2.2375 | 0.7450 |
| C(19) | 2.5008 | 2.9373 | -1.2986 |
| C(20) | 4.1026 | 3.5168 | 0.9126 |
| H(21) | 3.8004 | 1.4624 | 1.4748 |
| C(22) | 3.0220 | 4.2129 | -1.1323 |
| H(23) | 1.8886 | 2.7198 | -2.1678 |
| C(24) | 3.8219 | 4.5066 | -0.0261 |
| H(25) | 4.7253 | 3.7402 | 1.7722 |
| H(26) | 2.8080 | 4.9820 | -1.8668 |
| H(27) | 4.2268 | 5.5054 | 0.0996 |
| N(28) | -0.5010 | -2.9893 | 0.4909 |
| H(29) | -1.1062 | -2.7357 | -0.2903 |
| H(30) | -0.9162 | -3.7533 | 1.0153 |
| P(31) | -0.8154 | 0.5626 | 1.7293 |
| O(32) | -0.0451 | 1.7336 | 2.1785 |
| O(33) | -2.1417 | 0.2398 | 2.5652 |
| H(34) | -2.6136 | 1.0378 | 2.8451 |
| O(35) | -0.0506 | -0.7836 | 1.6801 |
| H(36) | -0.4165 | -1.6781 | 1.2514 |
| O(37) | -1.3782 | 0.8428 | 0.2160 |
| P(38) | -2.5746 | 0.0775 | -0.5468 |
| O(39) | -2.7698 | -1.3231 | -0.1149 |
| O(40) | -2.2132 | 0.3009 | -2.0691 |
| H(41) | -1.4651 | -0.3111 | -2.2960 |
| O(42) | -3.7816 | 1.0714 | -0.2894 |
| C(43) | -5.0984 | 0.6639 | -0.7426 |
| C(44) | -6.0751 | 1.7375 | -0.3244 |
| H(45) | -5.3368 | -0.3016 | -0.2886 |
| H(46) | -5.0694 | 0.5495 | -1.8304 |
| H(47) | -6.0774 | 1.8485 | 0.7619 |
| H(48) | -5.8095 | 2.6944 | -0.7779 |
| H(49) | -7.0809 | 1.4637 | -0.6498 |

**TS1**

[SMD(DMF)/M062X/6-31G(d,p)] G: -1999.490729 a.u.

| | | | |
|---|---|---|---|
| C(1) | 5.2996 | -1.6261 | 1.4143 |
| C(2) | 4.2479 | -0.7936 | 1.8002 |
| C(3) | 3.2000 | -0.5376 | 0.9230 |
| C(4) | 3.2125 | -1.1334 | -0.3564 |
| C(5) | 4.2475 | -1.9913 | -0.7154 |
| C(6) | 5.3029 | -2.2305 | 0.1623 |
| H(7) | 6.1138 | -1.8068 | 2.1081 |
| H(8) | 4.2387 | -0.3474 | 2.7895 |

| | | | |
|---|---|---|---|
| H(9) | 4.2233 | -2.4613 | -1.6933 |
| H(10) | 6.1143 | -2.8876 | -0.1286 |
| C(11) | 2.0558 | -0.9100 | -1.2598 |
| O(12) | 1.5669 | -1.9924 | -1.8020 |
| C(13) | 1.4744 | 0.3356 | -1.3699 |
| C(14) | 2.0194 | 1.4075 | -0.6334 |
| H(15) | 0.5129 | 0.4372 | -1.8586 |
| H(16) | 3.1002 | 1.4781 | -0.5359 |
| C(17) | 1.2717 | 2.6336 | -0.3878 |
| C(18) | 1.9542 | 3.8446 | -0.2076 |
| C(19) | -0.1280 | 2.6057 | -0.3025 |
| C(20) | 1.2403 | 5.0161 | 0.0140 |
| H(21) | 3.0389 | 3.8621 | -0.2564 |
| C(22) | -0.8393 | 3.7720 | -0.0606 |
| H(23) | -0.6417 | 1.6543 | -0.3855 |
| C(24) | -0.1526 | 4.9792 | 0.0867 |
| H(25) | 1.7667 | 5.9562 | 0.1374 |
| H(26) | -1.9217 | 3.7304 | 0.0128 |
| H(27) | -0.7056 | 5.8948 | 0.2692 |
| N(28) | 2.1480 | 0.3614 | 1.2568 |
| H(29) | 1.2171 | -0.0650 | 1.2119 |
| H(30) | 2.2931 | 0.8329 | 2.1448 |
| P(31) | -3.3142 | 0.7927 | 0.3213 |
| O(32) | -3.8254 | 2.1037 | -0.1031 |
| O(33) | -4.4780 | -0.2372 | 0.6636 |
| H(34) | -4.1731 | -1.1632 | 0.6252 |
| O(35) | -2.3112 | 0.8333 | 1.5441 |
| H(36) | -1.5487 | 0.1966 | 1.4294 |
| O(37) | -2.4669 | 0.0345 | -0.8445 |
| P(38) | -1.4897 | -1.2576 | -0.5695 |
| O(39) | -0.5887 | -0.9080 | 0.5901 |
| O(40) | -0.9085 | -1.6679 | -1.8921 |
| H(41) | 0.5692 | -1.8594 | -2.0148 |
| O(42) | -2.6450 | -2.2816 | -0.0831 |
| C(43) | -2.2104 | -3.5470 | 0.4732 |
| C(44) | -3.4429 | -4.3349 | 0.8512 |
| H(45) | -1.5758 | -3.3425 | 1.3401 |
| H(46) | -1.6200 | -4.0695 | -0.2858 |
| H(47) | -4.0249 | -3.8006 | 1.6061 |
| H(48) | -4.0709 | -4.5071 | -0.0252 |
| H(49) | -3.1477 | -5.3015 | 1.2647 |

**13**

[SMD(DMF)/M062X/6-31G(d,p)] G: -709.121200947 a.u.

| | | | |
|---|---|---|---|
| C(1) | 2.8499 | -2.2486 | 0.0360 |
| C(2) | 1.7861 | -1.8939 | 0.8588 |
| C(3) | 1.2589 | -0.5982 | 0.8134 |
| C(4) | 1.8316 | 0.3442 | -0.0693 |
| C(5) | 2.8833 | -0.0377 | -0.9028 |
| C(6) | 3.4009 | -1.3287 | -0.8552 |
| H(7) | 3.2423 | -3.2595 | 0.0843 |
| H(8) | 1.3533 | -2.6196 | 1.5412 |
| H(9) | 3.2900 | 0.6766 | -1.6133 |
| H(10) | 4.2175 | -1.6143 | -1.5083 |
| C(11) | 1.2664 | 1.6998 | -0.0573 |
| O(12) | 1.9292 | 2.6907 | -0.7252 |
| C(13) | 0.1059 | 1.9601 | 0.5565 |
| C(14) | -0.6850 | 0.8634 | 1.2212 |
| H(15) | -0.3043 | 2.9636 | 0.5432 |
| H(16) | -1.1430 | 1.2661 | 2.1316 |
| C(17) | -1.8169 | 0.3108 | 0.3557 |
| C(18) | -1.6353 | 0.0613 | -1.0078 |
| C(19) | -3.0395 | -0.0153 | 0.9457 |
| C(20) | -2.6560 | -0.5112 | -1.7619 |
| H(21) | -0.6927 | 0.3246 | -1.4791 |
| C(22) | -4.0658 | -0.5818 | 0.1921 |
| H(23) | -3.1889 | 0.1792 | 2.0052 |
| C(24) | -3.8744 | -0.8326 | -1.1644 |
| H(25) | -2.5038 | -0.7009 | -2.8198 |
| H(26) | -5.0135 | -0.8234 | 0.6629 |
| H(27) | -4.6715 | -1.2722 | -1.7552 |
| N(28) | 0.2386 | -0.1934 | 1.6581 |
| H(29) | 2.8787 | 2.5153 | -0.6935 |
| H(30) | -0.2289 | -0.9570 | 2.1321 |

## 8. References

[1] Zhao, Y.; Truhlar, D.G. The M06 suite of density functionals for main group thermochemistry, thermochemical kinetics, noncovalent interactions, excited states, and transition elements: two new functionals and systematic testing of four M06-class functionals and 12 other functionals. *Theor. Chem. Account.* **2008**, *120*, 215-241.

[2] Ochterski, J.W.; Petersson, G.A.; Montgomery Jr, J.A. A complete basis set model chemistry. V. Extensions to six or more heavy atoms. *J. Chem. Phys.* **1996**, *104*, 2598-2619.

[3] Grisafi, A.; Wilkins, D.M.; Csányi, G.; Ceriotti, M. Symmetry-adapted machine learning for tensorial properties of atomistic systems. *Phys. Rev. Lett.*, **2018**, *120*, 036002.

[4] Lu, T.; Chen, Q. van der Waals potential: an important complement to molecular electrostatic potential in studying intermolecular interactions. *J. Mol. Model.* **2020**, *26*, 315.

[5] Peterson, K.I.; Pullman, D.P. Determining the structure of oxalate anion using infrared and Raman spectroscopy coupled with Gaussian calculations. *J. Chem. Educ.* **2016**, *93*, 1130-1133.
